# Glycoproteomics of a Single Protein: Revealing Tens of Thousands of Myozyme Glycoforms by Hybrid HPLC-MS Approaches

**DOI:** 10.1016/j.mcpro.2023.100622

**Published:** 2023-07-20

**Authors:** Fiammetta Di Marco, Constantin Blöchl, Wolfgang Esser-Skala, Veronika Schäpertöns, Tao Zhang, Manfred Wuhrer, Koen Sandra, Therese Wohlschlager, Christian G. Huber

**Affiliations:** 1 Department of Biosciences and Medical Biology, Bioanalytical Research Labs, University of Salzburg, Salzburg, Austria; 2 Christian Doppler Laboratory for Innovative Tools for Biosimilar Characterization, University of Salzburg, Salzburg, Austria; 3 Center for Proteomics and Metabolomics, Leiden University Medical Center, Leiden, The Netherlands; 4 Department of Biosciences and Medical Biology, Computational Systems Biology Group, University of Salzburg, Salzburg, Austria; 5 Research Institute for Chromatography (RIC), Kortrijk, Belgium

**Keywords:** recombinant human acid alpha-glucosidase, Myozyme, hybrid HPLC-MS, SAX-HPLC-MS, glycosylation, glycoforms, glycoproteomics, intact protein, mannose-6-phosphate, phosphorylated glycoforms, enzymatic dissection, data integration, MoFi, annotations

## Abstract

Characterization of highly glycosylated biopharma-ceuticals by mass spectrometry is challenging because of the huge chemical space of coexistent glycoforms present. Here, we report the use of an array of HPLC-mass spectrometry–based approaches at different structural levels of released glycan, glycopeptide, and hitherto unexplored intact glycoforms to scrutinize the biopharmaceutical Myozyme, containing the highly complex lysosomal enzyme recombinant acid α-glucosidase. The intrinsic heterogeneity of recombinant acid α-glucosidase glycoforms was unraveled using a novel strong anion exchange HPLC-mass spectrometry approach involving a pH-gradient of volatile buffers to facilitate chromatographic separation of glycoforms based on their degree of sialylation, followed by the acquisition of native mass spectra in an Orbitrap mass spectrometer. Upon considering the structures of 60 different glycans attached to seven glycosylation sites in the intact protein, the large set of interdependent data acquired at different structural levels was integrated using a set of bioinformatic tools and allowed the annotation of intact glycoforms unraveling more than 1,000,000 putative intact glycoforms. Detectable isoforms also included several mannose-6-phosphate variants, which are essential for directing the drug toward its target, the lysosomes. Finally, for the first time, we sought to validate the intact glycoform annotations by integrating experimental data on the enzymatically dissected proteoforms, which reduced the number of glycoforms supported by experimental evidence to 42,104. The latter verification clearly revealed the strengths but also intrinsic limitations of this approach for fully characterizing such highly complex glycoproteins by mass spectrometry.

## Highlights

• Hybrid mass spectrometry approach to characterize Myozyme at the different structural levels.
• Intact Myozyme analysis by a novel strong anion exchange-HPLC-mass spectrometry approach enabling the separation of proteoforms based on their sialylation degree and acquisition of native mass spectra in a semi-automated fashion.
• Enzymatic dissection with peptide:N-glycosidase F and sialidase to reduce spectral complexity at intact protein level.
• Multilevel data integration using the software MoFi to annotate intact protein mass spectra.
• Application of the approach to calculate the percentage of the biologically relevant phosphorylated glycoforms.

## Graphical Abstract

**Figure:**
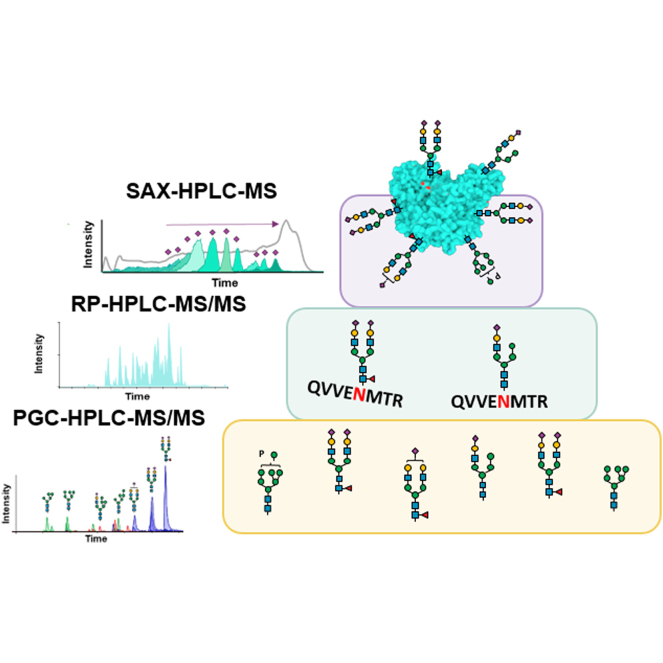

## In Brief

Compared to the total number of mammalian genes, the structural diversity of glycoproteins as a consequence of posttranslational glycosylation is enormous. We propose an integrated HPLC-mass spectrometry approach to explore the highly glycosylated protein recombinant acid alpha-glucosidase (Myozyme). The glycosylation complexity of this protein is reflected in a huge chemical space of more than 40,000 glycoforms, which were experimentally revealed through HPLC-mass spectrometry analyses and bioinformatic data integration at different structural levels of released glycans, glycopeptides, and intact glycoforms.

A draft of the human proteome assembled from more than 16,000 proteome analyses provided protein evidence for more than 92% of approximately 20,000 human genes annotated in Swiss-Prot (1). Contrarily, recent estimations of the entire complexity of the human proteome predict a total number of individual proteoforms exceeding several millions (2). The substantial difference between the number of protein-encoding genes and the number of different proteoforms is due to several sources of protein structural variation such as sequence polymorphisms, alternative splicing, or post-translational modifications (PTMs). It is thus not surprising that this biological complexity is also found in biopharmaceutical proteins expressed in biological systems. In fact, recent studies provided experimental proof for the presence of hundreds to thousands of glycoforms of therapeutic proteins expressed in mammalian host cells (3, 4, 5).

The vast chemical space of pharmaceutical glycoprotein structures exerts a great impact on the efficacy and safety of a drug product. The coexistence of a plethora of these distinct glycoforms also renders their analytical characterization extremely challenging (6). To scrutinize these complex systems, we and others have previously demonstrated that a hybrid mass spectrometry (MS) approach involving the characterization of the glycoprotein at different levels of structural complexity is the key to unravel the intrinsic glycoform heterogeneity (5, 7, 8, 9, 10). Conventional methods, such as released glycan (11, 12) and glycopeptide (13) analysis provide information on the glycan structure and on the site of occupancy. However, these approaches are not sufficient to accomplish characterization of intact glycoforms, that is, the biologically relevant compound.

In the past decade, native MS has become the method of choice to study intact proteins while maintaining their quasi-native conformation (14, 15, 16, 17, 18, 19). A benefit of this technique is the preservation of protein higher order structure, resulting in reduced solvent accessibility of residues and hence in lower charge states upon electrospray ionization (ESI). With respect to mass spectra, this translates into a decreased overlap of *m/z* signals that spread over a larger *m/z* range in the raw mass spectrum, increasing the spatial resolution than the MS of proteins under denaturing conditions (4). Furthermore, mass spectral complexity can also be reduced by glycosidase digestion of the intact glycoprotein to facilitate the annotation of intact glycoforms (4, 10). As we demonstrated in previous studies, bioinformatic data integration of the different structural levels is a crucial aspect to assign the glycoforms of a complex glycoprotein (5, 20).

Recently, novel semi-automated approaches applying native separation techniques such as strong cation exchange (SCX) HPLC coupled to MS have been employed for the characterization of biopharmaceutical proteoforms (21, 22, 23, 24, 25, 26). In contrast to established SCX methods using a gradient of nonvolatile salt in the mobile phase, a pH-gradient based on volatile buffering components is compatible with MS. Separation of distinct proteoforms is predominantly based on differences in pIs, resulting in the elution of the protein variant at a pH close to its pI. Hitherto, this approach has been used to study intact mAb charge variants (21, 22, 23, 24, 25, 26). However, for proteins exhibiting an acidic pI, strong anion exchange (SAX) HPLC-MS is better suited to separate glycoforms on the basis of negative charge, for example, the number of sialic acid residues. To date, only three studies in the literature report the use of SAX-HPLC-MS to separate and detect proteoforms (27, 28, 29), one of which deals with the characterization of the biopharmaceutical erythropoietin (28).

Myozyme is an orphan drug containing the recombinant lysosomal enzyme human acid α-glucosidase (r-hGAA) expressed in Chinese hamster ovary cells. It is used as enzymatic replacement therapy for the treatment of Pompe disease (30). r-hGAA consists of a ≈99.5 kDa amino acid chain with seven *N*-glycosylation sites (N84, N177, N334, N414, N596, N826, and N869) and associated glycans, resulting in a total molecular mass of approximately 110 kDa (31). The therapeutic protein is delivered to the lysosome *via* the cation-independent and the cation-dependent mannose-6-phosphate receptors, where it fulfills its intended glycogenolytic function. Therefore, the presence of mannose-6-phosphate groups on oligomannose and hybrid type *N*-glycans attached to r-hGAA is of crucial importance for the lysosomal uptake of the protein (32, 33).

However, the level of mannose-6-phosphate in r-hGAA is relatively low, and a high drug dosage is necessary to reach adequate clearance of the lysosomal glycogen (32). To improve protein targeting, glycoengineered GAAs were developed with an increased level of mannose-6-phosphate either by chemical conjugation (34, 35), by production from transgenic animal milk for example, rabbit (36) and mice (37) or by expression in different organisms such as yeast (38, 39).

The structural characterization of r-hGAA poses a challenge due to the exceptional heterogeneity of the glycan structures (hybrid, complex, and oligomannose type, which may be additionally phosphorylated or acetylated), the different glycan abundances, and the high number of r-hGAA glycosylation sites. Previous studies reported a partial characterization of r-hGAA at the level of released glycans and glycopeptides (40, 41, 42, 43). In these studies, oligomannose, hybrid-, and complex-type glycan structures were reported also embedding *O*-acetylation of sialic acids (41) and phosphorylation of oligomannose structures (40, 41, 42, 43). Moreover, pyroglutamate formation from cyclization of *N*-terminal glutamine in r-hGAA was reported in the 2007 Japanese report of the deliberation results by the Pharmaceutical Medical Devices Agency (44). Hitherto, however, no intact mass spectral data of r-hGAA was reported. Our strategy is based on r-hGAA characterization at intact protein level using a novel native SAX-HPLC-MS method, which aims at separating intact r-hGAA glycoforms based on their degree of sialylation and at acquiring native mass spectra upon hyphenation with a Q Exactive Plus Hybrid Quadrupole-Orbitrap mass spectrometer. To unravel the spectral complexity obtained for this protein, a set of bioinformatics tools is employed to integrate the data of released glycans and glycopeptides up to the intact glycoform level in a stepwise approach. Finally, the annotations of intact glycoforms are filtered *in silico*, based on the masses of the experimentally desialylated protein species.

## Experimental Procedures

### Materials

Residual vials of Myozyme (Genzyme Europe B.V., batch 9W0864, expiration date 11/2021) were supplied by a local hospital. DTT, guanidine hydrochloride (Gnd-HCl), ammonium acetate (AmAc), ammonium bicarbonate (AmBi), ammonium formate (AmF), iodoacetamide, acetic acid (HOAc), and formic acid (FA) were purchased from Sigma-Aldrich. Sodium acetate was purchased from Fluka Analytical. Trypsin was purchased from Promega. Sialidase (neuraminidase from *Arthrobacter ureafaciens*) was purchased from Roche Diagnostics GmbH and Rapid peptide:N-glycosidase F (PNGase F) (nonreducing format) from New England Biolabs. LC-MS grade acetonitrile (ACN) was purchased from VWR chemicals. Water (H_2_O) was purified in-house by a MilliQ Integral 3 system from Merck Millipore.

### Sample Preparation and Enzymatic Dissection

*N*-glycans were released and analyzed according to an established polyvinylidene difluoride (Millipore) membrane–based glycan release workflow using a 96-well plate format (12, 45). Briefly, 20 μg of r-hGAA were dot-blotted on the polyvinylidene difluoride membrane, denatured with 5.8 mol L^−1^ Gnd-HCl (Thermo Fisher Scientific), and reduced using 5.0 mmol L^−1^ DTT (Sigma-Aldrich) by incubation at 60 °C for 30 min. After washing with water, 2.0 U of PNGase F (Roche Diagnostics) were added and incubated at 37 °C overnight together with the internal standard (10 ng maltoheptaose DP7; Elicityl). After collection of released *N*-glycans, an acidification step in approximately 6.0 mmol L^−1^ AmAc (pH 5.0; Sigma-Aldrich) for 1.0 h at room temperature was carried out, and samples were subsequently dried by vacuum centrifugation. Afterwards, *N*-glycans were transformed into their alditol forms in a reduction step upon resuspension in 20 μl of 50 mmol L^−1^ KOH (Honeywell Fluka, Thermo Fisher Scientific) and 1.0 mol L^−1^ NaBH_4_ (Sigma-Aldrich) at 60 °C for 3.0 h. Desalting of *N*-glycans was performed on a SCX resin (Dowex 50 W X8; Merck) self-packed into 96-well filter plates (Orochem Technologies). The H_3_BO_3_ formed during the reaction was co-evaporated with methanol in a vacuum centrifuge. A further purification step was conducted on Carbograph material (Grace Discovery Sciences) self-packed into 96-well filter plates. After drying in a vacuum centrifuge, purified released glycans were resuspended in 10 μl of H_2_O.

To obtain tryptic glycopeptides, 30 μg of protein (5.0 μg/μl) was denatured and reduced for 1 h at 50 °C under shaking (900 rpm) in 3.0 mol L^−1^ Gnd-HCl and 50 mmol L^−1^ DTT for a total volume of 40 μl solution containing 125 mmol L^−1^ AmBi. Alkylation was performed in 50 mmol L^−1^ iodoacetamide for 1.0 h at 22 °C in the dark while shaking (900 rpm). The alkylated protein was buffer exchanged using Micro Bio-Spin P-30 column (Bio-Rad Laboratories) in 20 mmol L^−1^ AmAc (pH 6.8) for a final volume of 60 μl. Tryptic digestion was carried out overnight at 37 °C adding 0.5 μg of trypsin (ratio 1:60 w:w).

r-hGAA was enzymatically de*-N*-glycosylated using rapid PNGase F (nonreducing format). Five micrograms of protein (5.0 μg/μl) was diluted with 1.0 μl 5X buffer (New England Biolabs) and 8.0 μl of H_2_O for a final volume of 10 μl and incubated for 5.0 min at 75 °C. Subsequently, 0.50 μl of rapid PNGase F was added, and the solution was incubated for 15 min at 50 °C for complete removal of *N*-glycans.

In addition, a reducing de-*N*-glycosylation protocol was used. Four microliters of 5X buffer (New England Biolabs) and 10 μl of 100 mmol L^−1^ DTT in H_2_O were added to 15 μg of protein (5.0 μg/μl) and incubated at 80 °C for 2.0 min. After cooling down, 1.0 μl of rapid PNGase F (nonreducing format) was added and incubated at 50 °C for 15 min. Despite the “nonreducing format,” the rapid PNGase F worked also in the presence of DTT.

For disulfide mapping, 5.0 μg of PNGase F-deglycosylated r-hGAA (0.50 μg/μl) were buffer exchanged using Micro Bio-Spin P-30 column (Bio-Rad Laboratories) in 20 mmol L^−1^ AmAc (pH 6.8) and digested overnight at 37 °C under shaking (900 rpm) with 0.25 μg trypsin (ratio 1:20 w:w).

Desialylation of intact r-hGAA was performed using sialidase. Seven hundred fifty micrograms of protein (5.0 μg/μl) was buffer exchanged with Micro Bio-Spin P-30 column into 40 mmol L^−1^ sodium acetate. Digestion was carried out with 75 mU sialidase (10 mU/μl) overnight at 37 °C while shaking (900 rpm).

Prior to SAX-HPLC-MS analysis, untreated and desialylated r-hGAA were buffer exchanged with Micro Bio-Spin P-30 column (Bio-Rad Laboratories) into 20 mmol L^−1^ AmAc (pH 6.8).

### Nano-PGC-HPLC-MS/MS Analysis of Released *N*-Glycans

Released *N*-glycan alditols were chromatographically separated using a Thermo Fisher Scientific Ultimate 3000 RSLCnano UHPLC system (Thermo Fisher Scientific) equipped with a self-packed trap column (5 μm particle diameter, 30 mm × 0.32 mm inner diameter [i.d.]) and a column (3 μm particle diameter, 100 mm × 0.075 mm i.d.) self-packed with Thermo Fisher Scientific Hypercarb KAPPA column packing material (Thermo Fisher Scientific). A column oven temperature of 45 °C was used for the separation, and the injection volume was 1.0 μL. Elution was carried out with mobile phase solution A (H_2_O supplemented with 10 mM AmBi) and mobile phase B (60% ACN supplemented with 10 mM AmBi). Glycans were injected into the trap at a flow rate of 6.0 μL min^−1^ and a mobile phase composition of 1.0% B that was held for 5 min. Subsequently, glycans were eluted by the nano-pump employing a multistep gradient of the following: 2 to 9% B in 1.0 min, 9.0 to 54% B in 100 min, 54 to 95% B in 9.0 min, 95% B for 8.0 min, 95 to 2% B in 5.0 min, and 2.0% B for 17 min at a flow rate of 500 nL min^−1^. The nano-HPLC system was coupled to an amaZon ETD speed ion trap mass spectrometer equipped with a CaptiveSpray nanoESI source (Bruker Daltonics) and isopropanol as dopant solvent. Mass spectrometric parameters are described in Zhang *et al*. (45) in detail. Glycans were analyzed by tandem mass spectrometry (MS/MS) using negative-mode ESI and collision-induced dissociation (CID), enabling structural elucidation of glycan species including many compositional isomers.

### Nano-RP-HPLC-MS/MS Analysis of Peptides

Glycopeptide and disulfide bridge analyses were performed on a Thermo Fisher Scientific Ultimate 3000 RSLCnano UHPLC system (Thermo Fisher Scientific) coupled with a Thermo Fisher Scientific Q Exactive Plus Hybrid Quadrupole-Orbitrap mass spectrometer (Thermo Fisher Scientific) where a Thermo Fisher Scientific Nanospray Flex Ion Source (Thermo Fisher Scientific) was installed. The source was equipped with a nanospray-fused silica emitter with pulled tip, outer diameter 360 μm, i.d. 20 μm, Tip i.d. 10 μm, length 12 cm (TIP1002010-12, CoAnn Technologies, LLC, MS Wil).

Chromatographic separation of glycopeptides was achieved using a Halo ES-C18 nano HPLC column (75 μm × 150 mm, 2.7 μm particle diameter, 160 Å) operated at 50 °C and a flow rate of 300 nL min^−1^. Eluent A comprised water with 0.10% FA, and eluent B comprised ACN with 0.10% FA. Initially, 1% B was held for 5 min, followed by a gradient from 1.0 to 30% B over 30 min with a sequential increase from 30 to 99% B in 25 min. 99% B was held for 5.0 min, and equilibration was carried out at 1% B for 10 min for a total run time of 65 min. The injection volume was 1.0 μL for an amount of protein injected of ≈ 500 ng.

Disulfide mapping was performed with a Thermo Fisher Scientific Acclaim PepMap RSLC (300 μm × 100 mm, 2 μm particle diameter, 100 Å C18 column, Thermo Fisher Scientific) operated at 50 °C at a flow rate of 1.2 μL min^−1^. Eluent A comprised water with 0.10% FA, and eluent B comprised ACN with 0.10% FA. Initially, 1.0% B was held for 5 min, followed by a gradient from 1.0 to 30% B over 30 min with a sequential increase from 30 to 99% B in 25 min. 99% B was held for 5.0 min, and equilibration was carried out at 1.0% B for 10 min for a total run time of 65.0 min. The injection volume was 2.0 μL for an amount of protein injected of ≈ 2.0 μg.

In both experiments, the ion-source spray voltage was set to 1.5 kV, capillary temperature to 250 °C, S-lens RF level to 60, and all source gases to 0. For MS^1^, the Orbitrap mass analyzer *m/z* range was set to *m/z* 400 to 3000 with a resolution setting of 70,000 at *m/z* 200 and one microscan, in-source CID to 0, positive polarity, and the automatic gain control (AGC) target was 3 × 10^6^ with a maximum injection time (IT) of 100 ms. For MS/MS a data-dependent acquisition mode was selected with a scan range *m/z* 200 to 2000 loop count of 10, the resolution setting of 17,500 at *m/z* 200, AGC target value was 1.0 × 10^5^. The maximum IT was set to 50 ms, microscans at 1, and spectral multiplexing count at 1. Isolation window was set at 2 *m/z* and normalized collision energy was 28. The dynamic exclusion was set at 10 s.

### RP-HPLC-MS Analysis of Deglycosylated Recombinant Acid α-Glucosidase

Deglycosylated r-hGAA analyses were carried out on an Ultimate 3000 UHPLC system (Thermo Fisher Scientific) coupled with a Q Exactive Plus Hybrid Quadrupole-Orbitrap mass spectrometer.

To separate the sialidase from r-hGAA, a C4 RP column Xbridge Protein BEH, 300, C4 300 Å, 3.5 μm particle diameter, 2.1 mm x 250 mm (Waters) was chosen. Mobile phase A comprised water with 0.10% FA, and mobile phase B comprised ACN with 0.10% FA. The column was held at 20% B for 5.0 min, followed by a gradient from 20% to 70% B in 45 min. Subsequently, 99% B was held for 5 min, and a column equilibration at 1% B was carried out for 15 min for a total run time of 70 min. Temperature was set at 50 °C and flow rate at 100 μL min^-1^. 2.5 μg of protein were injected per run (5 μL injection).

The Q Exactive Plus mass spectrometer was set to acquire data in standard pressure mode with the full MS^1^ detection scan range from *m/z* 500 to 3000, in-source CID at 50.0 eV, positive polarity, resolution settings of 17,500 at *m/z* 200, microscans set to 10, AGC target at 3e6, maximum IT at 200 ms. The heated ESI source spray voltage was set at 4 kV, sheath gas at 10 (arbitrary units), auxiliary gas at 5, S-Lens RF level at 100, probe heater temperature at 80 °C, and capillary temperature at 250 °C.

### SAX-HPLC-MS Analysis of Intact Recombinant Acid α-Glucosidase

Intact and desialylated r-hGAA analyses were carried out on an Ultimate 3000 UHPLC system (Thermo Fisher Scientific) coupled with a Q Exactive Plus Hybrid Quadrupole-Orbitrap mass spectrometer. A ProPac SAX-10 anion exchange guard column 10 μm particle diameter, 2.0 mm x 50 mm, nonporous (Thermo Fisher Scientific) comprising quaternary ammonium groups as functional groups was used to separate r-hGAA proteoforms. Mobile phase A consisted of 10 mmol L^−1^ of AmF and 10 mmol L^−1^ of AmAc (pH 6.8), and mobile phase B consisted of 10 mmol L^−1^ of HOAc and 10 mmol L^−1^ of FA (pH 2.9). The column was operated at 200 μL min^-1^ and at 30 °C.

For intact r-hGAA, the column was held at 10% B for 5.0 min, followed by an increase from 10% to 20% B in 1.5 min. A gradient from 20% to 90% B from 6.5 to 26.5 min was carried out, followed by a flushing step at 99% B for 4.5 min and an equilibration step at 10% B for 15 min. Total run time was 45 min. A relatively high amount of 150 μg protein (50 μL injection volume) needed to be injected in order to compensate for the low-ionization efficiency.

For desialylated r-hGAA, the gradient was adapted to take into account the different pI values of proteoforms after removal of sialic acids. Initially, 1.0% B was held for 5.0 min, followed by a gradient from 1.0% to 90% B in 30 min. A flushing step at 99% B for 5 min and an equilibration step at 1.0% B for 15 min were carried out for a total run time of 50 min.

The Q Exactive Plus mass spectrometer equipped with the BioPharma Option was used in high mass range mode with a trapping gas pressure setting of 1.5 and no spectrum averaging. Spray voltage was set at 3.6 kV, sheath gas at 20, auxiliary gas at 5, S-Lens RF level at 200, probe heater temperature at 200 °C, and capillary temperature at 200 °C. The scan range was from 2500 to 8000 *m/z*, the in-source CID was set at 100.0 eV, polarity was set positive, resolution setting of 17,500, ten microscans were averaged, AGC target was set at 3e6, and maximum IT at 200 ms.

### MS Data Processing and Stepwise Data Integration Across the Different Structural Levels

Identification of released *N*-glycan structures was based on the detected MS^1^ mass and corresponding MS^2^ spectra. Manual annotation of fragments in the MS^2^ spectrum was performed employing GlycoWorkbench 2.1 (46), taking into account the theoretical knowledge of glycan fragmentation patterns in negative ion mode when using CID (47, 48) and common knowledge of *N*-glycan biosynthesis. Reference MS^2^ fragment spectra (when available) were obtained from UniCarb-DB (49). A list of glycan structures identified is reported in supplemental Table S1 and the corresponding MS evidence is presented in supplemental information 2, glycan structural elucidation. Since these released *N*-glycans served as an exploratory library to interpret the glycopeptide data, all detected glycans were included. This library therefore also contains seven glycans that showed strong signal at the MS^1^ level but lack a corresponding MS^2^ spectrum. Semiquantitation of *N*-glycans was performed using Skyline (v20.2.0.343, MacCoss Lab, Department of Genome Science, University of Washington) with the small molecule interface (50).

Peptide identification based on MS^2^ spectra was performed using PMI-Byonic (v3.11.3, Protein Metrics Inc). Parameters were set as follows: cleavage residues RK, cleavage side C terminal, digestion specificity fully specific, missed cleavages 0, precursor and fragment mass tolerance 50 ppm, and fragmentation-type CID low energy. Modifications were customized with carbamidomethylated C fixed, Gln→pyro-Glu NTerm common1, deamidated N rare1, oxidized M, and W rare2. The library of *N*-glycans added for identification is reported in supplemental Table S1 and was built based on released *N*-glycan data. The modification was set at common 1. Total common and total rare max modification were both set at 2.

Glycopeptide relative quantification based on extracted ion chromatograms (XICs) of MS^1^ ions was carried out using Skyline (51). A list of glycopeptides with the corresponding *N*-glycan structures identified in PMI-Byonic was added in Skyline, and peak identification and integration were manually validated based on the isotopic pattern matching, a mass error below 25 ppm, and the retention time. Skyline transition settings were set as follows: filter peptide precursor charges 1, 2, 3, 4, 5, and 6; ion charges 1; ion types p; instrument *m/z* range min 50 and max 2500; method match tolerance *m/z* 0.055; full-scan MS^1^ filtering isotope peaks included percent; precursor mass analyzer Orbitrap; min % of base peak 5%; and resolving power 70,000 at *m/z* 200. From the glycopeptide data evaluation results, a site-specific library of *N*-glycans was built, reporting the structures with their relative abundances and the site of the modification.

Deconvolution of raw mass spectra of intact r-hGAA to zero-charge spectra was accomplished using the ReSpect algorithm embedded in Thermo Fisher Scientific BioPharma Finder software v. 3.0 with the sliding window deconvolution feature (Thermo Fisher Scientific).

For the assignment of the peaks in the deconvoluted mass spectra, the annotation tool MoFi was used (20). This software assigns glycoform composition for each peak by application of a two-stage search algorithm that finds the glycoforms fitting with the experimental masses and compiles a hierarchical list of them based on the relative abundance of the glycopeptide. The mass tolerance between the theoretical and experimental mass was set at 5 Da.

### Experimental Design and Statistical Rationale

We attempted the characterization of recombinant acid α-glucosidase glycoforms upon collecting information at different structural levels of released glycans, glycopeptides, and intact glycoforms. First, the structures of the individual glycans present in the glycoprotein were qualitatively elucidated by nano-porous graphitized carbon (PGC)-HPLC-MS/MS based on the MS^1^ and MS^2^ glycan spectra acquired in a single run. Second, the attachment of individual glycans to the different glycosylation sites in the protein were revealed based on glycopeptide analysis by nano-reversed-phase (RP)-HPLC-MS/MS in technical triplicates. The Byonic site-specific glycan identification was manually validated using Skyline, considering the matching between the theoretical and experimental isotopic pattern, a mass error below 25 ppm, and the retention time of the glycopeptide. Skyline was also used for semiquantitation of glycopeptides based on the integration of XICs of MS^1^ spectra of the glycopeptide in technical triplicates. Third, the combination of the individual glycans and glycosylation sites to form a discrete protein glycoform was unraveled with the annotation tool MoFi for a single SAX-HPLC-MS run of intact glycoforms. Moreover, enzymatic dissection of intact glycoforms using PNGase F or sialidase was performed in a single run to qualitatively validate the annotations of the intact glycoforms. An outline of how different analytical approaches were implemented in order to obtain the structural information necessary at the three levels as well as the bioinformatic tools employed to evaluate and interconnect the data is provided in supplemental Fig. S1 and discussed in the MS data processing paragraph and in the result session below.

## Results

### Revealing the Heterogeneous Glycoprofile of Recombinant Acid α-Glucosidase by Nano-PGC-HPLC-MS/MS

To investigate *N*-glycan heterogeneity of r-hGAA (supplemental Fig. S2), *N*-glycans were enzymatically released with PNGase F, isolated, chemically reduced to the alditol forms, purified, and analyzed by nano-PGC-HPLC-MS/MS. Due to the high selectivity of PGC, efficient separation of glycan structural and linkage isomers was achieved (52). To increase the sensitivity of the method, ESI was conducted with the use of isopropanol as dopant solvent (53). The acquisition of MS^2^ spectra in negative ionization mode was crucial for the elucidation of *N*-glycan structures based on diagnostic fragments.

**Fig. 1:**
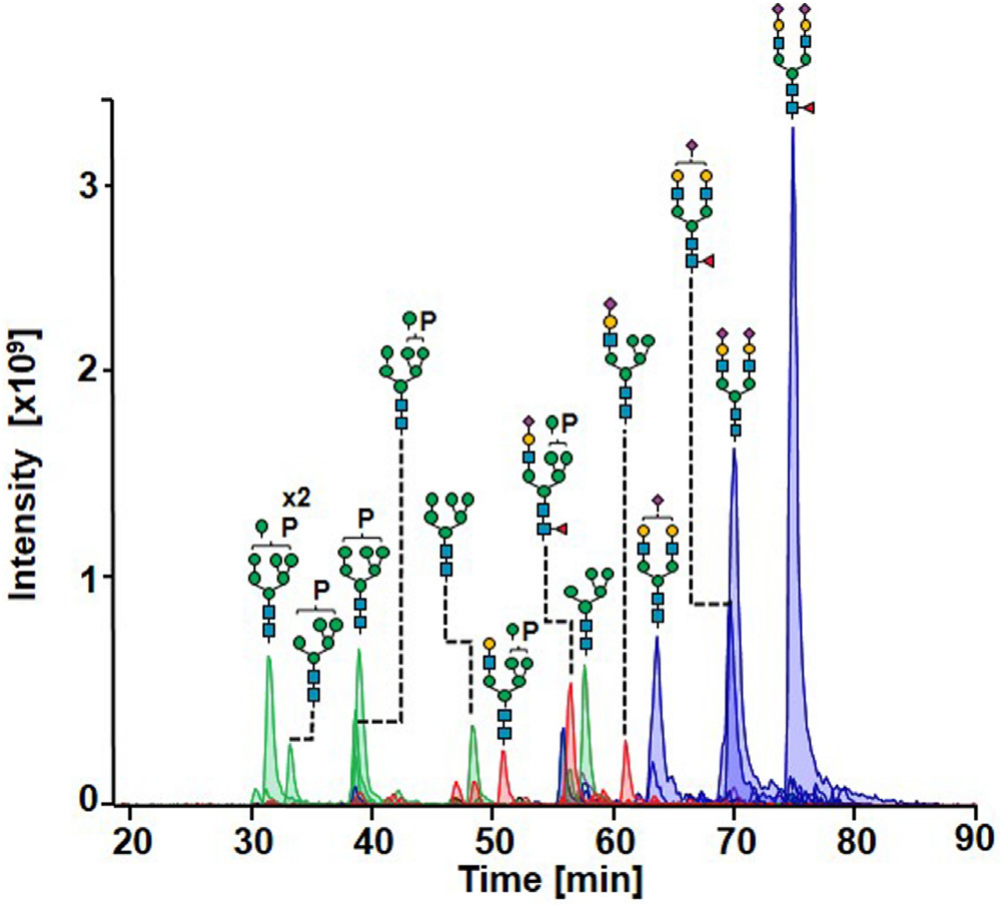
**Released *N*-glycan analysis of r-hGAA by PGC-HPLC-MS/MS.** Extracted ion chromatograms (XICs) of the most abundant glycan structures involving different classes are as follows: oligomannose (*green*), hybrid (*red*), and complex (*blue*) type. P indicates the presence of a phosphoryl group on a mannose residue. MS/MS, tandem mass spectrometry; PGC, porous graphitized carbon; r-hGAA, recombinant human acid alpha-glucosidase.

The glycoprofile of r-hGAA exhibited all four *N*-glycan classes of paucimannose, oligomannose, hybrid, and complex types (Fig. 1). Of note, phosphorylated hybrid and oligomannose *N*-glycans as well as complex glycans carrying *O*-acetylated sialic acids were also present. While phosphorylation on the glycans is a stable modification, acetylation on sialic acids is rather labile and may be lost during sample preparation due to the basic conditions used when reducing the released *N*-glycans to alditols (12). Thus, using this method, acetylated structures can be underestimated. In total, 49 different *N*-glycan compositions were identified (60 structures including isomers, see Supplementary Information 2, N-glycan structure elucidation) and semiquantified *via* XIC integration using Skyline (supplemental Fig. S3). Complex-type *N*-glycans were the most prominent (72.1%), followed by oligomannose type (16.4%), hybrid (11.5%), and paucimannose type (0.5%). Released *N*-glycan analyses revealed 19.8% of total glycans to carry one or two phosphoryl groups, of which 7.4% were oligomannose with one phosphoryl group, 4.0% oligomannose with two phosphoryl groups, and 8.4% hybrid glycans with one phosphoryl group. Oligomannose *N*-glycans spanned from a minimum of four to a maximum of eight mannose residues (M4–M8). Complex *N*-glycans were mainly biantennary (65.5%) but also monoantennary (1.1%), triantennary (4.7%), and tetra-antennary (0.8%). The core-fucosylated complex *N*-glycans were 43.2% of the total abundance against the 28.9% afucosylated. Most of the complex *N*-glycans were partially or completely sialylated (68.9%). *N*-glycans comprising *O*-acetylated sialic acids were also present at 2.9% of abundance. Of note, complex *N*-glycans carrying *N*-glycolylneuraminic acids were found at low abundance (2.1%). Twelve hybrid-type *N*-glycans were identified with different degree of core-fucosylation, sialylation, and phosphorylation.

All identified *N*-glycan structures were collected in a qualitative glycan structure library that was later used for glycopeptide identification in Byonic (supplemental Table S1). Moreover, to take into account the possible loss of acetyl groups in acetylated *N*-glycans due to sample preparation, all 17 acetylated *N*-glycan structures identified by Park *et al.* in a previous study (41) were considered for glycopeptide identification and inserted in the glycan structure library.

### Mapping Glycopeptides by Site-Specific Semiquantitative Analysis Using Nano-RP-HPLC-MS/MS

**Fig. 2:**
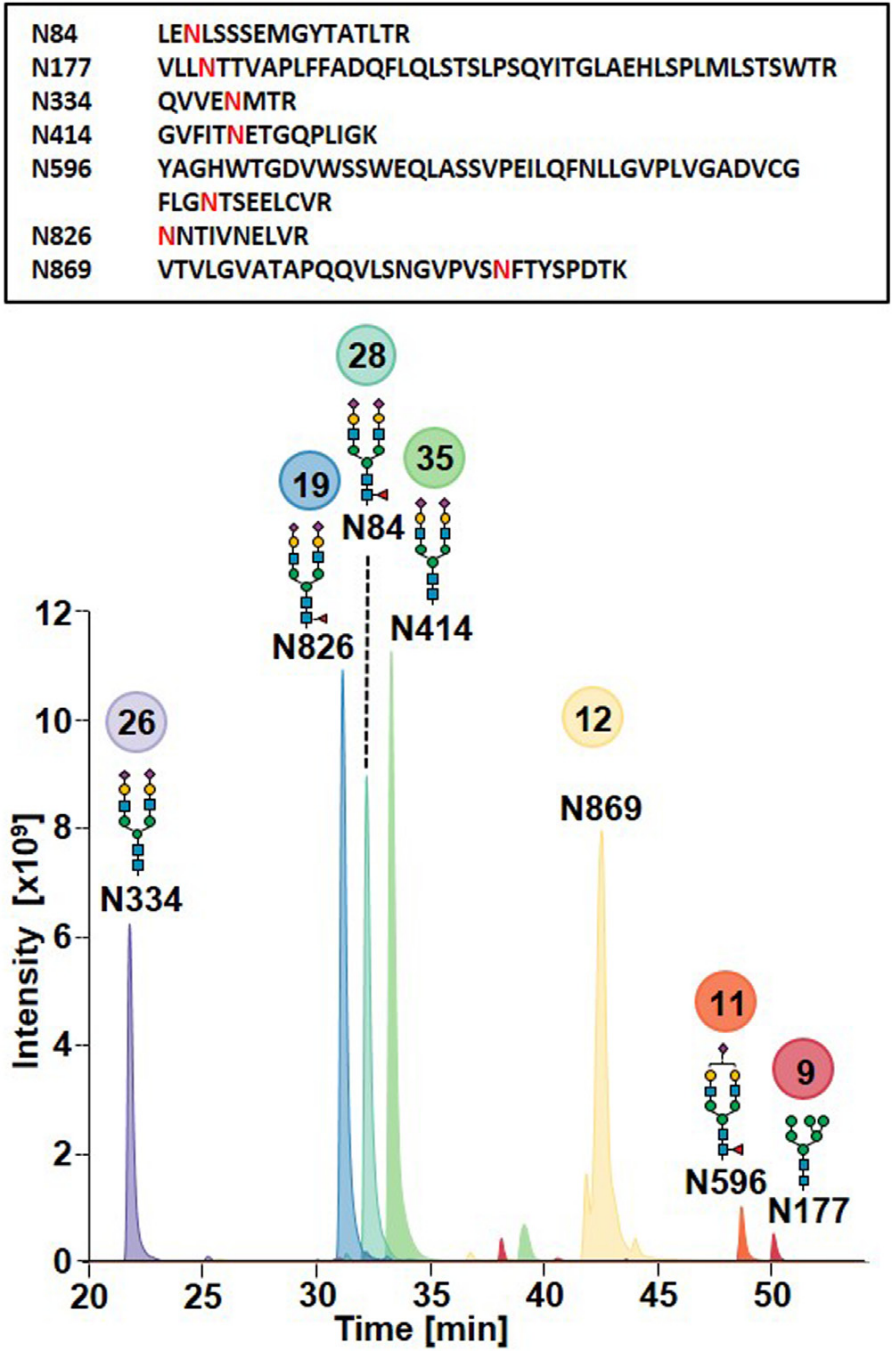
**Tryptic glycopeptide analysis by nano-RP-HPLC-MS/MS.** Extracted ion chromatograms (XICs) of the most abundant *N*-glycan structures for each glycosylation site retrieved from Skyline are reported. Site N869 is mainly deglycosylated. The number of glycovariants identified for each site is indicated in *colored circles*. In the *upper box*, the peptide sequences with glycosylation sites highlighted in *red* can be found. MS/MS, tandem mass spectrometry; RP, reversed-phase; XIC, extracted ion chromatogram.

Glycopeptides were obtained upon tryptic digestion and analyzed by nano-RP-HPLC-MS/MS. Using this method, the seven r-hGAA peptides carrying *N*-glycosylation sites (N84, N177, N334, N414, N596, N826, and N869) were separated based on their hydrophobicity (Fig. 2). The glycovariants of the same peptide eluted closely together independently from the different glycan structures attached while the corresponding unmodified peptide eluted approximately 1 to 2 min later. Byonic was used to identify glycan compositions present on each site using the qualitative glycan structure library built from released *N*-glycan data (supplemental Table S1). Subsequently, site-specific semiquantitation of glycopeptides was carried out using the open-source software Skyline based on XIC integration at MS^1^ level.

A list of glycopeptides identified in Byonic was built for Skyline, and the entries were validated and considered for semiquantitation only when matching the expected retention time, a mass error below 25 ppm, and the expected isotopic pattern. In the supplementary material, XICs of the different glycovariants for each glycosylation site retrieved from Skyline (supplemental Fig. S4, *A*–*G*) and bar charts of the relative abundances of the different glycan compositions per site (supplemental Fig. S5, *A*–*G*) are reported.

The sites N84, N596, and N826 showed a predominance of core-fucosylated and sialylated biantennary complex *N*-glycans, while sites N334 and N596 carried afucosylated and sialylated biantennary complex *N*-glycans. Site N869 was mainly unmodified and site N177 carried predominantly oligomannose and hybrid *N*-glycans (Fig. 2). Sites N84 and N414, and in a very low–amount site N177, were the only ones carrying mannose-6-phosphate oligomannose or hybrid *N*-glycans, with 30% of N84 and 33.2% N414 glycans carrying phosphoryl groups. Altogether, upon averaging across the seven glycosylation sites, 9.4% of glycopeptides were embedding mannose-6-phosphate groups in oligomannose or hybrid *N*-glycans.

This percentage is different from the one calculated based on released *N*-glycan data because at glycopeptide level also unmodified peptides are considered, lowering the total percentage of mannose-6-phosphate groups. However, when comparing released glycans and the global glycopeptide data (averaged for the seven glycosylation sites) omitting the unmodified peptides, the data are overall in accordance, and all the glycan structures identified at released glycan level were confirmed at glycopeptide level (supplemental Fig. S6). Moreover, a good match between the trends of the fractional abundances of the different glycan structures in released glycans and glycopeptide data was observed notwithstanding minor differences in individual glycans (Supplement Fig. S6). Additionally, nine acetylated glycan structures reported by Park *et al.* in a previous study (41), not identified at released glycan level because of the labile nature of acetyl groups (see previous section “Revealing the Heterogenous Glycoprofile…”), were detected at low abundances (supplemental Fig. S6). The numbers of different glycan structures identified per peptide are reported in Figure 2 (circles, Fig. 2). These structures can combine at intact glycoform level giving rise to a possible number of 10^9^ different glycoforms. To transfer this information to the next level of structural complexity, a site-specific semiquantitative *N*-glycan library reporting the glycan structures and fractional abundance per site was compiled and subsequently used to annotate intact glycoforms (Supplement xlsx file, Site_specific semi_quantitative glycan library cut-off 1%).

### Acquiring Native Mass Spectra of Recombinant Acid α-Glucosidase Intact Glycoforms by SAX-HPLC-MS

**Fig. 3:**
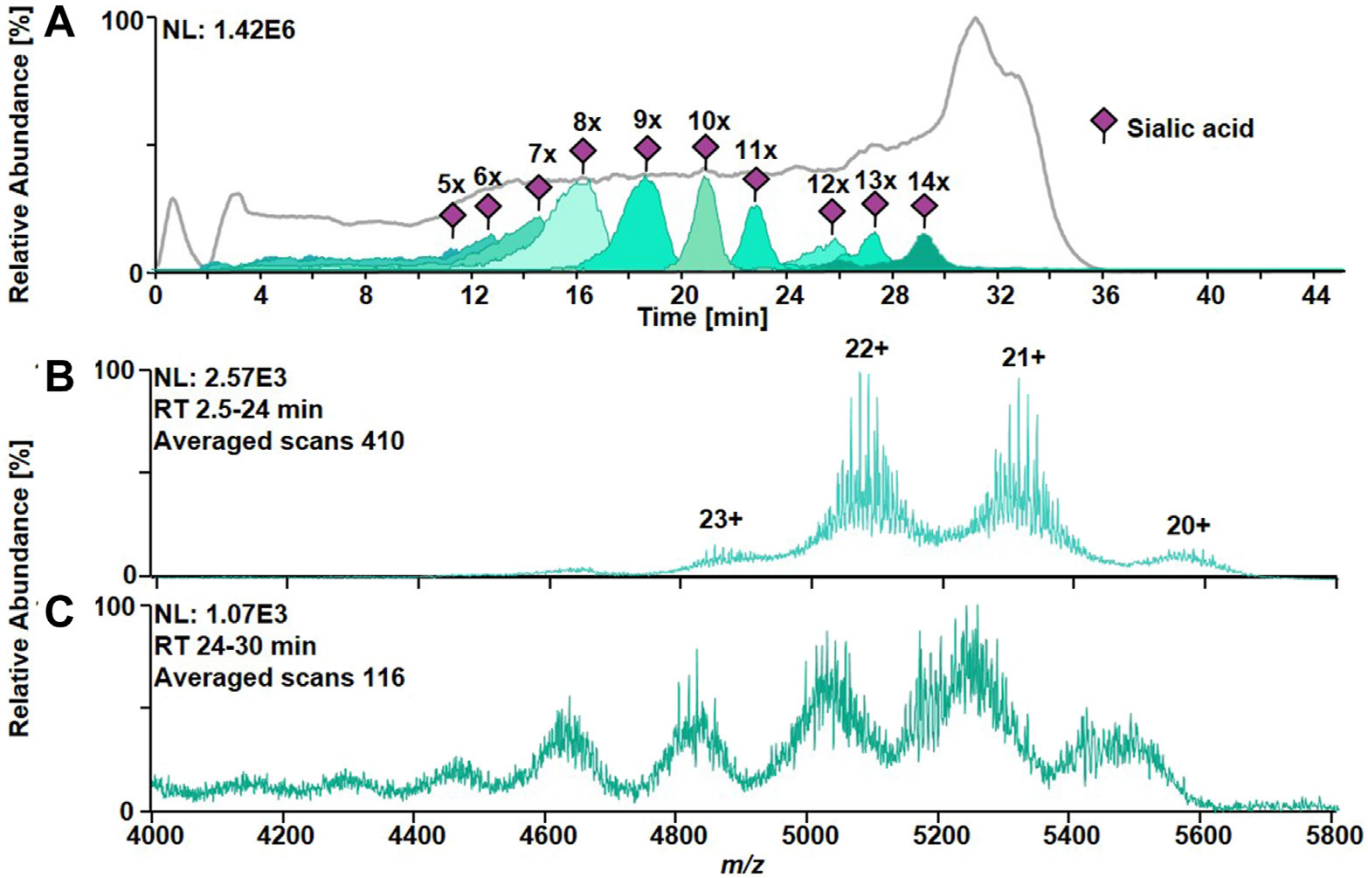
**SAX-HPLC-MS analysis of intact glycoforms of Myozyme.***A*, in *gray*, the total ion current chromatogram (TICC) obtained upon elution of glycoforms in a range of approximately 30 min is reported. The extracted ion chromatograms (XICs) of different ions of r-hGAA glycoforms carrying an increasing number of sialic acids are represented in *green shading*. Increased chromatographic retention is correlated with a higher degree of sialylation of glycoforms. *B*, raw mass spectrum associated to the retention time window from 2.5 to 24 min (410 averaged scans). A charge state distribution from 20 to 23 positive charges indicates the preservation of a quasi-native state of the glycoforms during the chromatographic separation. *C*, raw mass spectrum associated to the retention time window from 24 to 30 min (116 averaged scans). Despite the acidic conditions of the mobile phases (≈ pH 4–3) in this range of the gradient, only slight denaturation of the protein was observed as indicated by an increased number of charge states. MS, mass spectrometry; r-hGAA, recombinant human acid alpha-glucosidase; SAX, strong anion exchange.

To analyze hitherto unexplored intact r-hGAA glycoforms, an analytical approach involving SAX-HPLC-MS was optimized using a chromatographic column embedding a quaternary-ammonium–based stationary phase. r-hGAA exhibits an acidic theoretical pI of 5.5 of the protein backbone but, due to its glycosylation state, the actual pI values of the different proteoforms range from 5.5 to 3.5, making it a perfect candidate to be analyzed by SAX-HPLC-MS. Since typically salt gradients are employed, SAX-HPLC is conventionally considered incompatible with MS. However, the use of volatile buffer in the mobile phases (AmF and AmAc, FA, and HOAc) facilitates ionization of proteins by means of ESI and subsequent acquisition of mass spectra. The use of a pH-gradient of buffers (pH from 6.4 to 3.3) allows the separation of proteoforms under native conditions based on the charges on the surface of the three-dimensional proteoform structure and on their different pI values. The latter depend on the degree of sialylation and phosphorylation of the respective glycoform. In Figure 3*A*, the total ion current chromatogram of intact r-hGAA analyzed by SAX-HPLC-MS is reported in gray. The extraction of glycovariant XICs with an increasing degree of sialylation enables visualization of the separation power of this chromatographic method (Fig. 3*A*). Moreover, we think that the retention of the different proteoforms is affected not only by sialylation but also by the number of phosphoryl groups present. When looking at the main peak embedding nine sialic acids in Figure 3*A*, only a single broad chromatographic peak spanning from 13 to 20 min can be observed, which is a consequence of the need to overload the column with 150 μg sample in order to obtain a good mass spectrum of this protein. In other words, we think that the chromatographic separation of phosphorylated variants was sacrificed to obtain decent MS data. All proteoforms of r-hGAA eluted within approximately 30 min of the chromatographic run and elution was based on the number of sialic acids present in the glycan structures, ranging from 5 to 14. This sequential elution of proteoforms (supplemental Fig. S7) allowed the acquisition of native mass spectra without the necessity of spectrum averaging during acquisition, as required for standard direct infusion native MS using a static nano-ESI source where the proteoforms are not separated and thus simultaneously ionized. The mass spectrum of r-hGAA averaged over the whole retention range showed a charge state envelope ranging from 20 to 23 charges (Fig. 3, *B* and *C*), suggesting the preservation of proteoform quasi-native conformation (supplemental Figs. S7 and S8) during the chromatographic separation despite the slightly acidic pH of the mobile phases.

### Deciphering Recombinant Acid α-Glucosidase Glycoform Composition by Stepwise Data Integration Across the Different Structural Levels

**Fig. 4:**
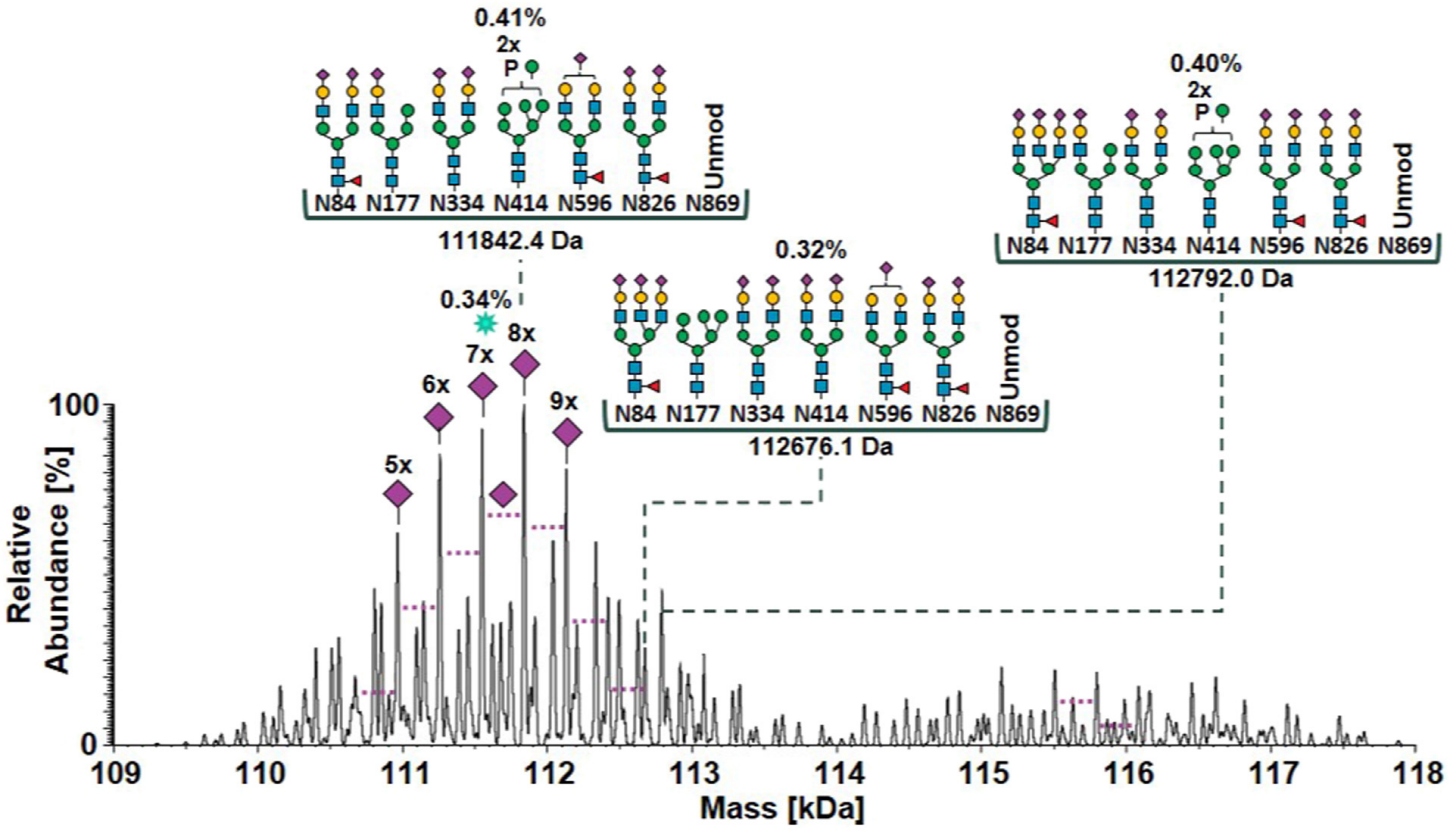
**Deconvoluted mass spectrum obtained by SAX-HPLC-MS analysis of intact glycoforms of Myozyme.** The four most abundant glycoforms annotated by MoFi are reported (see Supplement csv file, Intact Myozyme annotations filtered 0.01cut-off, for the complete annotation lists). The percentage indicates the fractional abundance carried by the glycoform. The third most abundant glycoform is indicated by *green asterisks*, and it carries the same structures of the glycoform of the mass 111842.4 Da with one sialic acid less on site N826 (sixth glycan). The second distribution of peaks (from 114 kDa to 118 kDa) is due to glycoforms where the site N869 is mainly glycosylated. MS, mass spectrometry; SAX, strong anion exchange.

To obtain a zero-charge spectrum, deconvolution of the mass spectra in the entire chromatographic range of proteoform elution was performed using the sliding window deconvolution feature embedded in BioPharma Finder software (54). This feature allowed the deconvolution of mass spectra associated to subsequent windows of retention time that are then summed to eventually obtain a single deconvoluted spectrum associated to all the chromatographic run windows. The deconvoluted mass spectrum of intact r-hGAA (Fig. 4) showed approximately 100 different masses spanning a range from 109 to 118 kDa with the most abundant signal corresponding to 111842.4 Da.

Before bioinformatic annotation of intact r-hGAA glycoforms, a PNGase F digest was performed on intact r-hGAA to de-*N*-glycosylate the protein and reveal possible PTMs present in the protein backbone. The enzymatically dissected protein was analyzed by RP-HPLC-MS. The chromatogram and the raw mass spectrum obtained together with the deconvoluted mass spectrum of PNGase F– treated r-hGAA are reported in supplemental Fig. S9.

Already at the glycopeptide level, cyclization of *N*-terminal glutamine to pyro-glutamate was observed (supplemental Fig. S10). When treated with PNGase F under nonreducing conditions, the theoretical mass of r-hGAA was expected to correspond to 99349.8 Da, considering six disulfide bridges (−12.1 Da), the conversion by PNGase F of glycosylated asparagines into deglycosylated aspartic acids (+7.1 Da), and the pyro-glutamate formation (−17 Da) (see supplemental Fig. S2). However, the experimental mass of nonreduced deglycosylated r-hGAA corresponded to 99464.9 Da (supplemental Fig. S10).

Since r-hGAA contains an odd number of cysteine residues, this mass shift was attributed to cysteinylation of the unpaired cysteine (C318). A PNGase F digest under reducing conditions resulted in an experimental mass of de-*N*-glycosylated r-hGAA of 99359.5 Da, confirming the loss of a cysteine after reduction (−119 Da of the cysteinylation +13 Da for cysteine reduction) (supplemental Fig. S11). Cysteinylation was further confirmed by disulfide bridge mapping in the analysis of a tryptic digest by capillary RP-HPLC-MS/MS (supplemental Fig. S12).

Once the amino acid sequence together with its PTMs present was retrieved, the bioinformatic tool MoFi (20) was used for annotation of the deconvoluted mass spectrum of intact r-hGAA obtained by SAX-HPLC-MS analysis. Cysteinylation and pyro-glutamate formation were set as fixed modifications and only glycopeptides with a fractional abundance equal or higher than 1% were considered for the site-specific semiquantitative glycan library to avoid explosion of the combinatorial search space with more entries. A mass tolerance of 5 Da was also set to consider the experimental mass uncertainty. Using this bioinformatics approach, we were able to annotate 1,190,724 putative intact glycoform structures based on the purely combinatorial model of MoFi. MoFi results in a list of annotated intact glycoforms (hits) that are weighed for the fractional abundance of the glycans attached. The contribution of a single glycoform to the intensity of the associated mass peak is scored from 0 to 1 (hit score) (Supplement csv file, intact Myozyme annotations).

From these annotations, a few questions arose: instead of the stochastic model underlying MoFi, could glycans combine following a chemical or rather biological logic, resulting in a smaller number of actual *N*-glycan structures? How could this experimentally be proven, and how could annotations be filtered accordingly? We addressed these questions taking into account the data of the enzymatically dissected protein.

### Filtering Recombinant Acid α-Glucosidase Intact Glycoform Annotations by Merging the Data of Experimentally and *In Silico* Desialylated Protein

Experimental desialylation of intact r-hGAA was conducted using a neuraminidase from *A. ureafaciens*, and the desialylated protein was analyzed by the SAX-HPLC-MS approach employed for the intact glycoform analysis, using a gradient optimized for the desialylated protein. The corresponding chromatogram and raw mass spectra are reported in supplemental Fig. S13, while the mirror plot of the deconvoluted spectra of desialylated and intact r-hGAA is reported in supplemental Fig. S14. The desialylated r-hGAA spectrum showed ≈ 60 signals in a mass range from 108 to 113.5 kDa. The shift to lower masses than the intact masses of r-hGAA glycoforms (109–118 kDa), together with the absence of Δm of 291 (the mass increment of a sialic acid residue) between mass peaks, indicated the completeness of the enzymatic digestion.

**Fig. 5:**
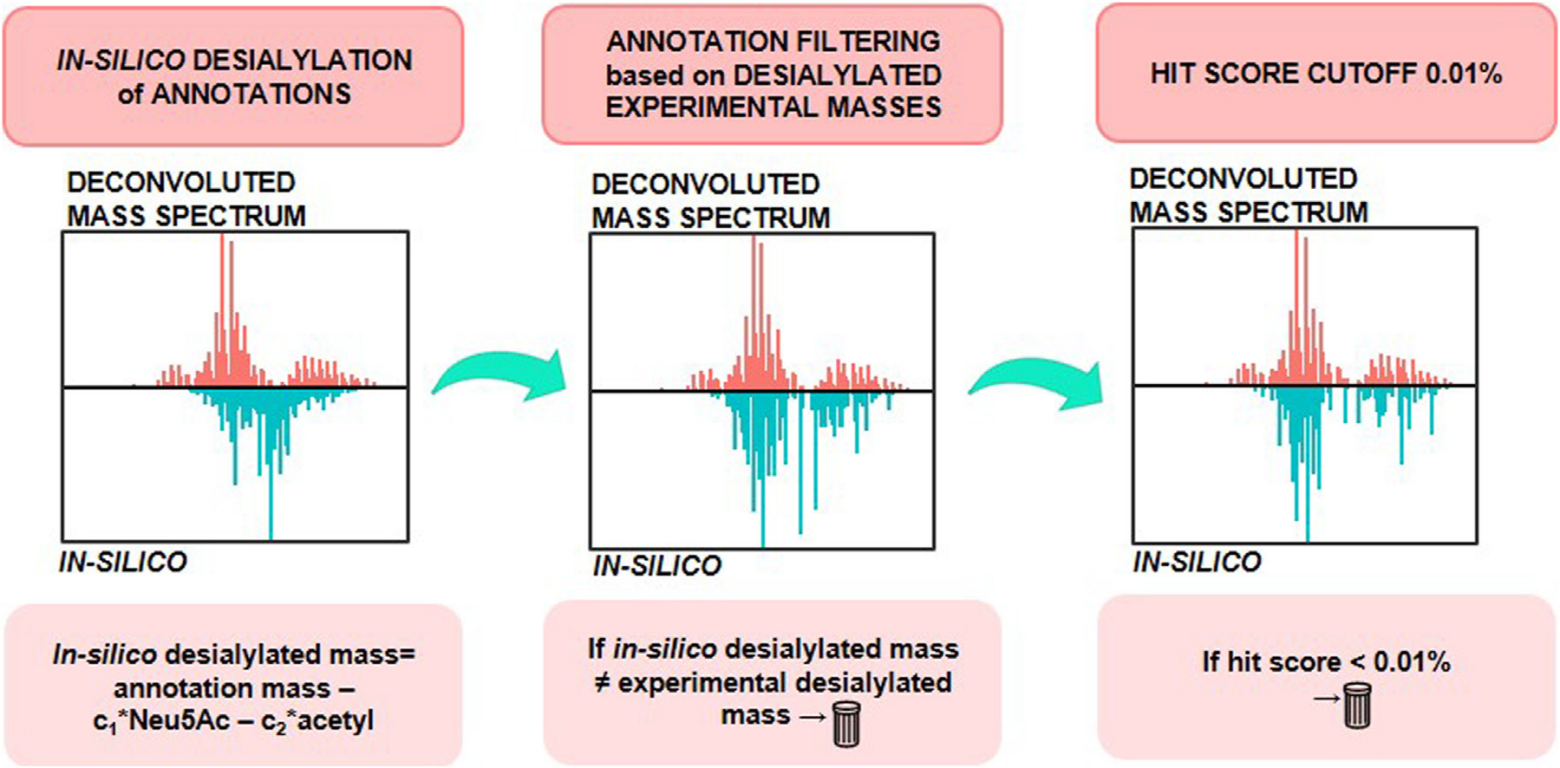
**Schematic workflow of *in silico* filtering of intact glycoform annotations based on experimentally desialylated protein data.** The *upper panels* show the deconvoluted experimental mass spectrum of r-hGAA after the neuraminidase digestion, while the *lower panels* show the simulated spectrum created upon *in silico* removal of sialic acids from all the potential MoFi annotations. Annotations were filtered based on a maximum mass error between the calculated and measured desialylated masses of 5 Da and subsequentially based on a hit score >0.01%. Magnified figures of the simulated spectra *versus* the experimentally desialylated spectrum are reported in supplemental Fig. S15. r-hGAA, recombinant human acid alpha-glucosidase.

Finally, we attempted to integrate the information embedded in an experimentally desialylated protein mass spectrum with the intact glycoform annotations. To our knowledge, this is the first report in literature of such an approach. We proceeded following two simple assumptions: if we removed *in silico* the sialic acids and the acetyl groups from MoFi intact annotations, the masses so calculated should fit with the experimentally desialylated spectrum, since these should be the residues removed enzymatically by the sialidase. Secondly, the distribution of the abundances of *in silico* desialylated glycoforms calculated should fit approximately with the glycoform abundance distribution in the spectrum of the experimentally desialylated protein. Based on these two assumptions, we corrected the annotations of the intact r-hGAA glycoforms in multiple steps: *i.* we computationally desialylated the intact glycoform annotations to obtain an *in silico* spectrum, *ii.* we computationally filtered desialylated masses based on the fitting with experimentally desialylated masses, *iii.* we normalized the glycoform annotation fractional abundances to 100% after filtering, *iv.* we attempted to fit glycoform distribution of *in silico* and experimental spectrum by removing the glycoforms with a hit score lower than 0.01%, *v.* Finally, we performed a second normalization to 100% of fractional abundances of the filtered annotations (Fig. 5 and supplemental Fig. S15 for zoom). The hit score cut-off criterion was considered to avoid that a multitude of low abundant glycoforms (in the order of hundreds of thousands), less probable from a combinatorial perspective, could contribute all together to a very high intense peak in the *in silico* desialylated spectrum, introducing a bias in the *in silico* desialylated glycoform distribution.

After the computational filtering of r-hGAA intact glycoform annotations based on desialylated masses and hit score cut-off of 0.01%, a total of 42,104 different glycoform structures were unraveled (Supplement csv file, Intact Myozyme annotations filtered 0.01cut-off). Of that, the most abundant structure (0.41% of the total fractional abundance) was annotated with A2S2F/A1S1-M4/A2S2/M7P2/A2G1S1F/A2S2F/Unmod and corresponded to the most abundant mass of 111842.4 Da in the deconvoluted mass spectrum (Fig. 4). For this peak, 224 alternative glycoforms were found by MoFi (Supplement csv file, intact Myozyme annotations filtered 0.01cut-off). The two series of signals observed in the mass ranges of 109 to 114 kDa and 114 to 118 kDa, respectively, originated from glycoforms showing the site N869 mainly unglycosylated or glycosylated, respectively. The fractional abundance of the glycoforms carrying at least one phosphoryl group (degree of phosphorylation), thus the biologically relevant glycoforms recognized by the mannose-6-phosphate receptor, was calculated from these filtered annotations and resulted in a portion of 67% of the intact glycoforms, with the majority of glycoforms containing one (30%), two (27% of which 14% with one site modified and 13% with two sites modified), or three (9%) phosphoryl groups.

## Discussion

In this study, we aimed at pushing the limits of complex glycoprotein characterization by HPLC-MS, focusing on the in-depth investigation of the biopharmaceutical Myozyme by a hybrid HPLC-MS approach at different structural levels. Released *N*-glycan analysis revealed a manifold glycoprofile comprising 49 different *N*-glycan structures of paucimannose, oligomannose, complex, and hybrid types. Phosphorylation on oligomannose and hybrid *N*-glycans was also readily detected. Integration of released glycan into glycopeptide data confirmed the high complexity of this protein, unraveling between 9 and 35 distinct glycan structures linked to the seven *N*-glycosylation sites of the protein (Fig. 2). We calculated that this could give rise to a possible number of combinations of intact glycoforms in the order of 10^9^.

The acquisition of intact mass spectra of r-hGAA glycoforms by a SAX-HPLC-MS approach enabled the separation of the proteoforms under quasi-native conditions, depending on their degree of sialylation (Fig. 3). The so-obtained raw mass spectra were deconvoluted and summed, and the resulting zero-charge mass spectrum showed ≈ 100 different signals (Fig. 4). A semiquantitative site-specific glycan library could be built from glycopeptide data to be used in MoFi (20) for annotation of the intact glycoform spectrum. Following a combinatorial model, MoFi was able to annotate ≈ 1,190,000 different glycoforms (Supplement csv file, intact Myozyme annotations).

Many intact glycoforms are isobaric and impossible to distinguish by MS at intact level, thus the only way to annotate the possible glycoforms present was using bioinformatic integration of information obtained at glycopeptide level. Due to the inherent inability of MS to resolve (almost) isobaric glycoforms, we relied on the quantitative information gained at the glycopeptide level, namely which glycans are attached to which glycosylation site in the protein. We are claiming that the presence of glycopeptides carrying different glycans represents the experimental evidence for the real existence of the protein glycoforms that we annotated. Subsequently, we distributed the observed intensity of certain glycans among all different glycoforms having glycan profiles that fit the total protein mass that we observed in the spectrum of the intact molecule. The distribution of intensities was based on the principal assumption that the presence of a certain glycan at one glycosylation site does not influence the attachment of glycans to other glycosylation sites.

In order to reduce the high number of combinatorially possible glycoforms, we followed an approach of experimental validation of actually present glycoforms through enzymatic dissection. This was based on the assumption that the computational removal of sialic acid(s) from all glycoforms containing sialic acid must yield a glycan profile, which can be verified in an experimental spectrum of r-hGAA treated with sialidase (see Fig. 5 and supplemental Figs. S14 and S15). Moreover, the large space of combinatorially possible glycoforms could be reduced by eliminating glycan profiles that only marginally contribute to the total signal intensity observable in the mass spectrum of intact r-hGAA. Thus, filtering for matching pairs of computationally and experimentally desialylated glycoforms and elimination of glycoforms having a hit score less than 0.01% yielded a set of 42,104 glycoforms, for which our analysis provided experimental evidence (Supplement csv file, intact Myozyme annotations filtered 0.01cut-off). We therefore think that we were able to resolve the complexity of the glycoprotein that is represented in the glycopeptides. Nevertheless, some glycopeptides could remain undetected due to their low abundances, and we also needed to filter very low–abundant signals of the intact glycoforms. In consequence, we assume that we annotated the first 42,000 most abundant glycoforms, but there will be more low-abundant glycoforms

Given the extreme structural diversity of glycoforms, the matching between *in silico* and experimentally desialylated data was quite decent and clearly corroborates our approach of deriving intact glycoform patterns upon integration of released glycan and glycopeptide data. The limitation of incomplete congruence between different structural levels of MS-based data was already highlighted with mathematical rigor by Compton *et al.*, who discussed in a recent paper the impossibility to fully estimate a modform (a proteoform where co-occurring PTMs are present in a specific combinatorial pattern) distribution from peptide or top-down MS data for a binary modification (*i.e.*, either absent or present at 100% in the modification site) (55). Moreover, the authors also demonstrated that the modform distribution estimation exponentially worsens as the number of modification sites increases.

Thus, it is not surprising that what proved to be impossible by Compton *et al*. for a relatively simple system (55) is even more critical when attempting to merge the experimentally and *in silico* desialylated spectra of such a complex system as the chemical space of r-hGAA glycoforms. In fact, we aimed to characterize at intact glycoform level a very complex glycoprotein containing seven glycosylation sites, focusing our attention on glycosylation that is not a binary modification but a complex one, since numerous different glycan structures may be present per site. Moreover, in the case of r-hGAA, the number of isobaric glycoforms is particularly high because of the extremely complex glycoprofile arising from the numerous different *N*-glycan structures identified comprising hybrid, complex, and oligomannose type also modified with phosphorylation or acetylation. Given all those complexities, our approach of *in silico* desialylation yields a spectrum that adequately resembles the experimental spectrum (supplemental Fig. S15).

From these results, we could shed light on the biologically relevant glycoforms of r-hGAA. Phosphorylation of oligomannose and hybrid *N*-glycans has been demonstrated to be crucial for the lysosomal uptake of the drug *via* the mannose-6-phosphate receptor. Based on the filtered glycoform annotations, the percentage of glycoforms carrying at least one phosphoryl group on the glycan structures was estimated as 67% of the total intact glycoforms. Biologically, this translates into the fact that only a maximum of two-thirds of the total glycoforms would be potentially targeted by the mannose-6-phosphate receptor into the lysosomes and execute their glycogenolytic function. This is a simplified assumption compared to the actual targeting of this enzyme. *De facto*, only a tiny fraction (≈1%) of the exogenous protein will reach, from the systemic circulation, the interstitial space where the cellular targeting *via* the mannose-6-phosphate in the skeletal muscle happens (56). Moreover, the affinity of the cation-independent mannose-6-phosphate receptor is higher for doubly phosphorylated glycoforms than the monophosphorylated ones and is highly dependent on the glycan structure and on the branch at which the phosphoryl group is attached. In fact, this could lead to a higher steric hindrance of the glycan structure and negatively impact the binding with the receptor domains (32). From released glycan data, we derived that 19.8% of all released glycans carry at least one phosphoryl group. Nonetheless, for docking to the mannose-6-phosphate receptor, a minimum of one of the seven glycosylation sites need to carry a phosphoryl group on the glycan structure, corresponding to 14.3% of the glycan abundances. Therefore, the presence of glycoforms that bind to the receptor needs to be estimated using a combinatorial approach at the glycopeptide or intact protein level. The phosphorylation degree at intact level (67%, see above) was corroborated quite well by the one calculated at glycopeptide level. Considering the intrinsic limitation of comparing lower with higher structural levels of glycosylation, the degree of phosphorylation at intact level turned out to be just slightly higher than expected from glycopeptide data, since 30.0% of site N84, 33.2% of site N414, and 2.7% of site N177 contained phosphorylated *N*-glycans leading to a maximum possible phosphorylation degree of ≈55% based on a purely combinatorial calculation (see supplemental Fig. S5, *A*–*G*).

It is important to mention that it is reasonable to expect differences between released glycan, glycopeptide, and intact data in terms of quantitative glycoform distribution (55). We think that these intrinsic limitations derive from different sources. First, it is experimentally impossible to acquire unbiased data by MS. As an example, there are different ionization efficiencies among the different structural levels; the ionization of released glycans is very much dependent on the glycan structure and the presence of negatively charged groups attached (phosphoryl and sialic acids) but is less critical for glycopeptides and should be neglectable for intact glycoforms. Moreover, intact analysis brings other experimental challenges, for example, the fact that for very complex glycoproteins some variants may be lost in the acquisition due to the overlapping of *m/z* signals or due to the low intensity of the peaks. This aspect is less critical for the acquisition of lower structural level data. Secondly, another bias may be introduced by processing of the raw data, in particular through the deconvolution step, as isotopically resolved mass spectra of such complex and large glycoproteins cannot be acquired. Deconvolution brings an additional level of uncertainty, particularly in the deconvoluted experimental mass error and in the relative intensity of the mass peaks that will never fit exactly the one in the raw mass spectrum.

Third, another bias comes from the data treatment. Similar or even identical masses of different glycoforms impede their discrimination by MS, thus a single mass peak is generally due to the contribution of numerous different (almost) isobaric glycoforms. The isobaricity exponentially increases with the number and the heterogeneity of the glycan structures attached to the intact glycoform. This means that the only way to discriminate the glycoforms can be achieved by bioinformatic tools. This implies that we rely on a purely combinatorial model for the annotations and, even if we filtered the annotations of the intact glycoforms *in silico* integrating the desialylation information into the workflow, we think that we did not fully resolve the complexity of the glycoprotein but we attempted to unravel as many glycoforms as possible based on experimental data (released glycans, glycopeptides, and enzymatically dissected intact glycoforms).

We think that some of these limitations will be overcome in the future with new instrumental technology allowing the discrimination of the isobaric glycoproteins (*e.g.*, separation *via* ion mobility or chromatography) and the acquisition of isotopically resolved mass spectra. On the other hand, new, more sophisticated bioinformatic tools incorporating chemical and/or biological information could help in the treatment of this complex dataset. To date, bioinformatic data integration among the different structural levels is the best approach to reduce these intrinsic limitations and characterize the chemical space of complex glycoproteins.

## Conclusions

The in-depth characterization of the exceptionally complex glycoprotein r-hGAA was attempted up to the intact glycoform level for the first time with a SAX-HPLC-MS approach. The investigation unraveled a huge chemical space of 42,104 glycoforms of which 67% are carrying phosphorylated *N*-glycans that can fulfill their biological function because they are potentially targeted by the mannose-6-phosphate receptor. From a methodological standpoint, the approach puts the basis to study highly glycosylated and/or phosphorylated proteins. Furthermore, the availability of a relatively fast and automatable SAX-HPLC-MS method for intact glycoform characterization is expected to provide another highly useful analytical tool for biopharmaceutical quality control in an industrial, GMP-regulated environment.

From a proteomic point-of-view, our study also suggests that the “structural diversity” of protein glycoforms expressed from a single gene in a mammalian organism significantly exceeds the “genetic diversity” of different protein sequences encoded in the mammalian genome. As the functional differences between different glycoforms of the same protein sequence can be expected to be very small relative to the functional differences of proteins of different sequence, the existence of this huge space of glycoforms is another indicator of the very delicate regulation and fine-tuning of protein functions and activities in the mammalian proteome.

## Data Availability

Raw files and byonic search results are available from Zenodo. (https://doi.org/10.5281/zenodo.7458010). All input files and data analysis scripts used in this study are freely available from GitHub (https://github.com/cdl-biosimilars/desialylation).

## Supplemental data

This article contains supplemental data.

## Conflict of interest

The authors declare the following competing financial interest(s): 10.13039/100004336Novartis AG/Sandoz GmbH as well as Thermo Fisher Scientific provided financial support for the Christian Doppler Laboratory for Innovative Tools for Biosimilar Characterization. The salaries of W. E.-S. and T. W. were fully funded; C. G. H. salary was partly funded by the Christian Doppler Laboratory for Biosimilar Characterization. The authors declare no other competing financial interest.

## Supplemental Data

140523_Myozyme supplement_revision_cleaned

20221212_N-Glycan_Structure_elucidation

290523_141222_Intact Myozyme annotations

290523_Intact Myozyme annotations filtered 0.01cutoff

Site_specific semi_quantitative glycan library cutoff 1%
