# Supplementary material for: Glycoproteomics of a Single Protein: Revealing Tens of Thousands of Myozyme Glycoforms by Hybrid HPLC-MS Approaches
Source: Mol Cell Proteomics. 2023 Jul 20;22(9):100622. doi: 10.1016/j.mcpro.2023.100622 (PMC10470421; doi:10.1016/j.mcpro.2023.100622)
Supplement: 140523_Myozyme supplement_revision_cleaned [file mmc1.docx]

**Supplementary information 1**

**Glycoproteomics of a single protein: revealing tens of thousands of Myozyme® glycoforms by hybrid MS approaches**

Fiammetta Di Marco^a,b^, Constantin Blöchl^c^, Wolfgang Esser-Skala^b,d^, Veronika Schäpertöns^a^, Tao Zhang^c^, Manfred Wuhrer^c^, Koen Sandra^e^ , Therese Wohlschlager^a,b^, Christian G. Huber^a,b^

a. Department of Biosciences and Medical Biology, Bioanalytical Research Labs, University of Salzburg, Hellbrunner Straße 34, 5020 Salzburg, Austria

b. Christian Doppler Laboratory for Innovative Tools for Biosimilar Characterization, University of Salzburg, Hellbrunner Straße 34, 5020 Salzburg, Austria

c. Center for Proteomics and Metabolomics, Leiden University Medical Center, Albinusdreef 2, 2333 ZA Leiden, The Netherlands

d. Department of Biosciences and Medical Biology, Computational Systems Biology Group, University of Salzburg, Hellbrunner Straße 34, 5020 Salzburg, Austria

e. Research Institute for Chromatography (RIC), President Kennedypark 26, 8500 Kortrijk, Belgium

**Table S1** Structures of Myozyme® N-glycans analysed by nano-PGC-HPLC-MS/MS. The glycan name together with the monosaccharide composition and the monoisotopic and average masses are reported. In the column MS/MS, glycans identified based on MS/MS spectra are indicated with a “yes”, while the ones lacking MS/MS spectra are indicated with “only MS1”. Highlighted in yellow, the acetylated N-glycan structures that were not identified in our analysis but that were reported in a previous publication^1^ are indicated and considered for glycopeptide identification (added manually). Annotated MS/MS spectra based on released glycan analysis can be found in supplementary information 2, N-glycan structure elucidation.

| Glycan | monosaccharide composition | Monoisotopic mass | Average mass | MS/MS |
| --- | --- | --- | --- | --- |
| M2F | HexNac(2)Hex(2)NeuAc(0)Fuc(1) | 876.32172 | 876.8052 | yes |
| M3 | HexNac(2)Hex(3)NeuAc(0)Fuc(0) | 892.31662 | 892.8046 | yes |
| M3F | HexNac(2)Hex(3)NeuAc(0)Fuc(1) | 1038.37442 | 1038.9454 | yes |
| M4 | HexNac(2)Hex(4)NeuAc(0)Fuc(0) | 1054.36932 | 1054.9448 | yes |
| A1G0 | HexNac(3)Hex(3)NeuAc(0)Fuc(0) | 1095.39588 | 1095.9966 | yes |
| M5 | HexNac(2)Hex(5)NeuAc(0)Fuc(0) | 1216.42202 | 1217.085 | yes |
| A1G0F | HexNac(3)Hex(3)NeuAc(0)Fuc(1) | 1241.45368 | 1242.1374 | only MS1 |
| A1G1 or A1G0-M4 | HexNac(3)Hex(4)NeuAc(0)Fuc(0) | 1257.44858 | 1258.1368 | yes |
| M5P | HexNac(2)Hex(5)NeuAc(0)Fuc(0)Phospho(1) | 1296.38834 | 1297.0649 | yes |
| M6 | HexNac(2)Hex(6)NeuAc(0)Fuc(0) | 1378.47472 | 1379.2252 | yes |
| M6P | HexNac(2)Hex(6)NeuAc(0)Fuc(0)Phospho(1) | 1458.44104 | 1459.2051 | yes |
| M6P2 | HexNac(2)Hex(6)NeuAc(0)Fuc(0)Phospho(2) | 1538.40736 | 1539.185 | yes |
| M7 | HexNac(2)Hex(7)NeuAc(0)Fuc(0) | 1540.52742 | 1541.3654 | yes |
| A1S1 | HexNac(3)Hex(4)NeuAc(1)Fuc(0) | 1548.54382 | 1549.3907 | yes |
| A1G1-M5 | HexNac(3)Hex(6)NeuAc(0)Fuc(0) | 1581.55398 | 1582.4172 | yes |
| A1S1Ac1 | HexNac(3)Hex(4)NeuAc(1)Fuc(0)Acetyl(1) | 1590.55436 | 1591.4273 | added manually |
| A2G1F | HexNac(4)Hex(4)NeuAc(0)Fuc(1) | 1606.58564 | 1607.4696 | yes |
| M7P | HexNac(2)Hex(7)NeuAc(0)Fuc(0)Phospho(1) | 1620.49374 | 1621.3453 | yes |
| A2G2 | HexNac(4)Hex(5)NeuAc(0)Fuc(0) | 1622.58054 | 1623.469 | yes |
| A1S1Ac2 | HexNac(3)Hex(4)NeuAc(1)Fuc(0)Acetyl(2) | 1632.5649 | 1633.4639 | added manually |
| A1G1-M5P or M6PGlcNAc | HexNac(3)Hex(6)NeuAc(0)Fuc(0)Phospho(1) | 1661.5203 | 1662.3971 | only MS1 for A1G1-M5P |
| A1S1F | HexNac(3)Hex(4)NeuAc(1)Fuc(1) | 1694.60162 | 1695.5315 | yes |
| M7P2 | HexNac(2)Hex(7)NeuAc(0)Fuc(0)Phospho(2) | 1700.46006 | 1701.3252 | yes |
| A1S1-M4 | HexNac(3)Hex(5)NeuAc(1)Fuc(0) | 1710.59652 | 1711.5309 | yes |
| A1S1Ac1F | HexNac(3)Hex(4)NeuAc(1)Fuc(1)Acetyl(1) | 1736.61216 | 1737.5681 | added manually |
| A2G0S1 | HexNac(4)Hex(4)NeuAc(1)Fuc(0) | 1751.62308 | 1752.5827 | yes |
| A2G2F | HexNac(4)Hex(5)NeuAc(0)Fuc(1) | 1768.63834 | 1769.6098 | yes |
| A1S1Ac2F | HexNac(3)Hex(4)NeuAc(1)Fuc(1)Acetyl(2) | 1778.6227 | 1779.6047 | added manually |
| M8P | HexNac(2)Hex(8)NeuAc(0)Fuc(0)Phospho(1) | 1782.54644 | 1783.4855 | yes |
| A2G0S1Ac1 | HexNac(4)Hex(4)NeuAc(1)Fuc(0)Acetyl(1) | 1793.63362 | 1794.6193 | added manually |
| A1G1F-M5P | HexNac(3)Hex(6)NeuAc(0)Fuc(1)Phospho(1) | 1807.5781 | 1808.5379 | yes |
| A1G1-M6P or M7PGlcNAc | HexNac(3)Hex(7)NeuAc(0)Fuc(0)Phospho(1) | 1823.573 | 1824.5373 | yes |
| A2G0S1Ac2 | HexNac(4)Hex(4)NeuAc(1)Fuc(0)Acetyl(2) | 1835.64416 | 1836.6559 | added manually |
| M8P2 | HexNac(2)Hex(8)NeuAc(0)Fuc(0)Phospho(2) | 1862.51276 | 1863.4654 | yes |
| A1S1-M5 | HexNac(3)Hex(6)NeuAc(1)Fuc(0) | 1872.64922 | 1873.6711 | yes |
| A2G1S1 | HexNac(4)Hex(5)NeuAc(1)Fuc(0) | 1913.67578 | 1914.7229 | yes |
| A2G1S1Ac1 | HexNac(4)Hex(5)NeuAc(1)Fuc(0)Acetyl(1) | 1955.68632 | 1956.7595 | added manually |
| A1G1F-M6P | HexNac(3)Hex(7)NeuAc(0)Fuc(1)Phospho(1) | 1969.6308 | 1970.6781 | yes |
| A2G1S1Ac2 | HexNac(4)Hex(5)NeuAc(1)Fuc(0)Acetyl(2) | 1997.69686 | 1998.7961 | only MS1 |
| Glycan | monosaccharide composition | Monoisotopic mass | Average mass | MS/MS |
| A2G1S1F | HexNac(4)Hex(5)NeuAc(1)Fuc(1) | 2059.73358 | 2060.8637 | yes |
| A2G1S1Ac1F | HexNac(4)Hex(5)NeuAc(1)Fuc(1)Acetyl(1) | 2101.74412 | 2102.9003 | added manually |
| A1S1-M6P | HexNac(3)Hex(7)NeuAc(1)Fuc(0)Phospho(1) | 2114.66824 | 2115.7912 | yes |
| A2G1S1Ac2F | HexNac(4)Hex(5)NeuAc(1)Fuc(1)Acetyl(2) | 2143.75466 | 2144.9369 | only MS1 |
| A2S2 | HexNac(4)Hex(5)NeuAc(2)Fuc(0) | 2204.77102 | 2205.9768 | yes |
| A2S1Sg1 | HexNac(4)Hex(5)NeuAc(1)Fuc(0)NeuGc(1) | 2220.76592 | 2221.9762 | yes |
| A2S2Ac1 | HexNac(4)Hex(5)NeuAc(2)Fuc(0)Acetyl(1) | 2246.78156 | 2248.0134 | added manually |
| A1S1F-M6P | HexNac(3)Hex(7)NeuAc(1)Fuc(1)Phospho(1) | 2260.72604 | 2261.932 | yes |
| A2S2Ac2 | HexNac(4)Hex(5)NeuAc(2)Fuc(0)Acetyl(2) | 2288.7921 | 2290.05 | only MS1 |
| A1S1-M6PGlcNAc | HexNac(4)Hex(7)NeuAc(1)Fuc(0)Phospho(1) | 2317.7475 | 2318.9832 | yes |
| A2S2Ac3 | HexNac(4)Hex(5)NeuAc(2)Fuc(0)Acetyl(3) | 2330.80264 | 2332.0866 | added manually |
| A2S2F | HexNac(4)Hex(5)NeuAc(2)Fuc(1) | 2350.82882 | 2352.1176 | yes |
| A2S1Sg1F | HexNac(4)Hex(5)NeuAc(1)Fuc(1)NeuGc(1) | 2366.82372 | 2368.117 | yes |
| A2S2Ac4 | HexNac(4)Hex(5)NeuAc(2)Fuc(0)Acetyl(4) | 2372.81318 | 2374.1232 | added manually |
| A2S2Ac1F | HexNac(4)Hex(5)NeuAc(2)Fuc(1)Acetyl(1) | 2392.83936 | 2394.1542 | added manually |
| A2S2Ac2F | HexNac(4)Hex(5)NeuAc(2)Fuc(1)Acetyl(2) | 2434.8499 | 2436.1908 | only MS1 |
| A1S1F-M6PGlcNAc | HexNac(4)Hex(7)NeuAc(1)Fuc(1)Phospho(1) | 2463.8053 | 2465.124 | yes |
| A2S2Ac4F | HexNac(4)Hex(5)NeuAc(2)Fuc(1)Acetyl(4) | 2518.87098 | 2520.264 | added manually |
| A3G0S2F | HexNac(5)Hex(5)NeuAc(2)Fuc(1) | 2553.90808 | 2555.3096 | only MS1 |
| A3G1S2F | HexNac(5)Hex(6)NeuAc(2)Fuc(1) | 2715.96078 | 2717.4498 | yes |
| A3S3 | HexNac(5)Hex(6)NeuAc(3)Fuc(0) | 2860.99822 | 2862.5629 | yes |
| A3S3F | HexNac(5)Hex(6)NeuAc(3)Fuc(1) | 3007.05602 | 3008.7037 | yes |
| A4S4F | HexNac(6)Hex(7)NeuAc(4)Fuc(1) | 3663.28322 | 3665.2898 | yes |

**Fig. S1** Schematic representation of the workflow used to analyse Myozyme®.

PGC-HPLC-MS/MS → Porous Graphitized Carbon High-Performance Liquid Chromatography - tandem Mass Spectrometry

RP-HPLC-MS/MS → Reversed Phase High-Performance Liquid Chromatography - tandem Mass Spectrometry

SAX-HPLC-MS → Strong Anion-Exchange High-Performance Liquid Chromatography - Mass Spectrometry

**Fig. S2** The amino acid sequence of recombinant acid alpha-glucosidase (r-hGAA), the lysosomal enzyme under the brand name Myozyme®. The asterisk on the N-terminal Q indicates its cyclization into N-terminal pyroglutamate and the N highlighted in red point out the 7 N-glycosylation sites. On the right the number of amino acid in the sequence, the position of the 7 N-glycosylation sites, the disulphide bridges are reported. In addition, the protein´s elemental composition, its average and monoisotopic molecular masses are listed.

**Fig. S3** All 49 N-glycan compositions (60 structures when counting the isomers, see supplementary information 2, N-glycan structure elucidation) and their fractional abundances (%) identified in the released glycan analysis by nano-PGC-HPLC-MS/MS. The two glycans containing N-glycolyl neuraminic acid (Sg), indicated by a box, were excluded for the glycopeptide identification because of the problem of coelution as well as isomericity with one N-acetyl neuraminic acid (S) and one oxidation that makes the semi-quantitation at glycopeptide level impossible at MS^1^ level.

**Fig. S4** Results of nano-RP-HPLC-MS/MS analysis of r-hGAA glycopeptides. Extracted ion current chromatograms retrieved from Skyline of the most abundant r-hGAA glycopeptides related to the glycosylation site (A) N84, (B) N177, (C) N334, (D) N414, (E) N596, (F) N826 and (G) 869. The structures of the glycans attached to the glycopeptides are reported. On the top, the sequence of the peptide is reported with the glycosylation site highlighted in red.

**Fig. S4** Continued.

**Fig. S4** Continued.

**Fig. S4** Continued.


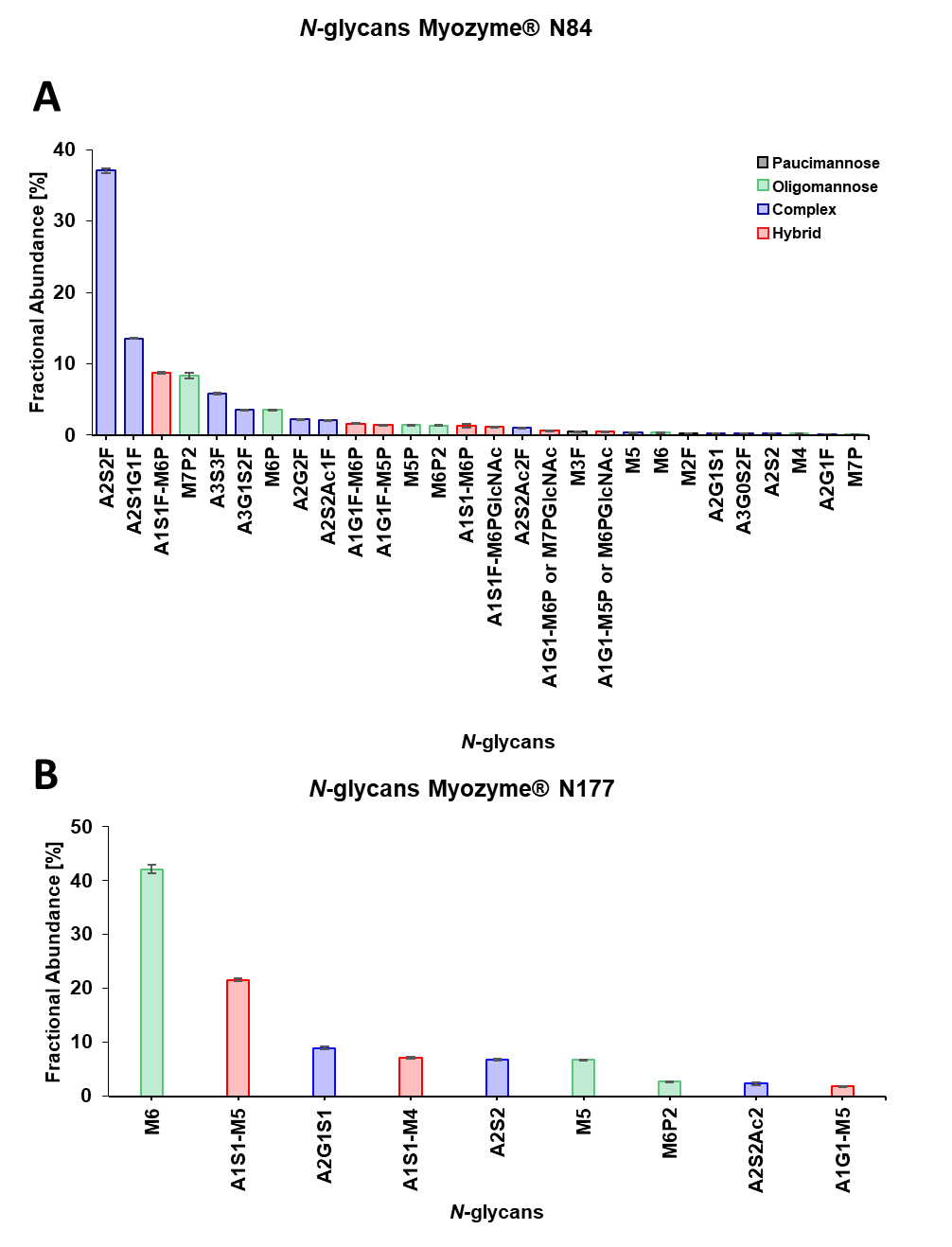


**Fig. S5** Results of nano-RP-HPLC-MS/MS analysis of r-hGAA glycopeptides. Bar charts reporting the structure and the fractional abundances of r-hGAA glycopeptides related to the glycosylation site (A) N84, (B) N177, (C) N334, (D) N414, (E) N596, (F) N826 and (G) N869.


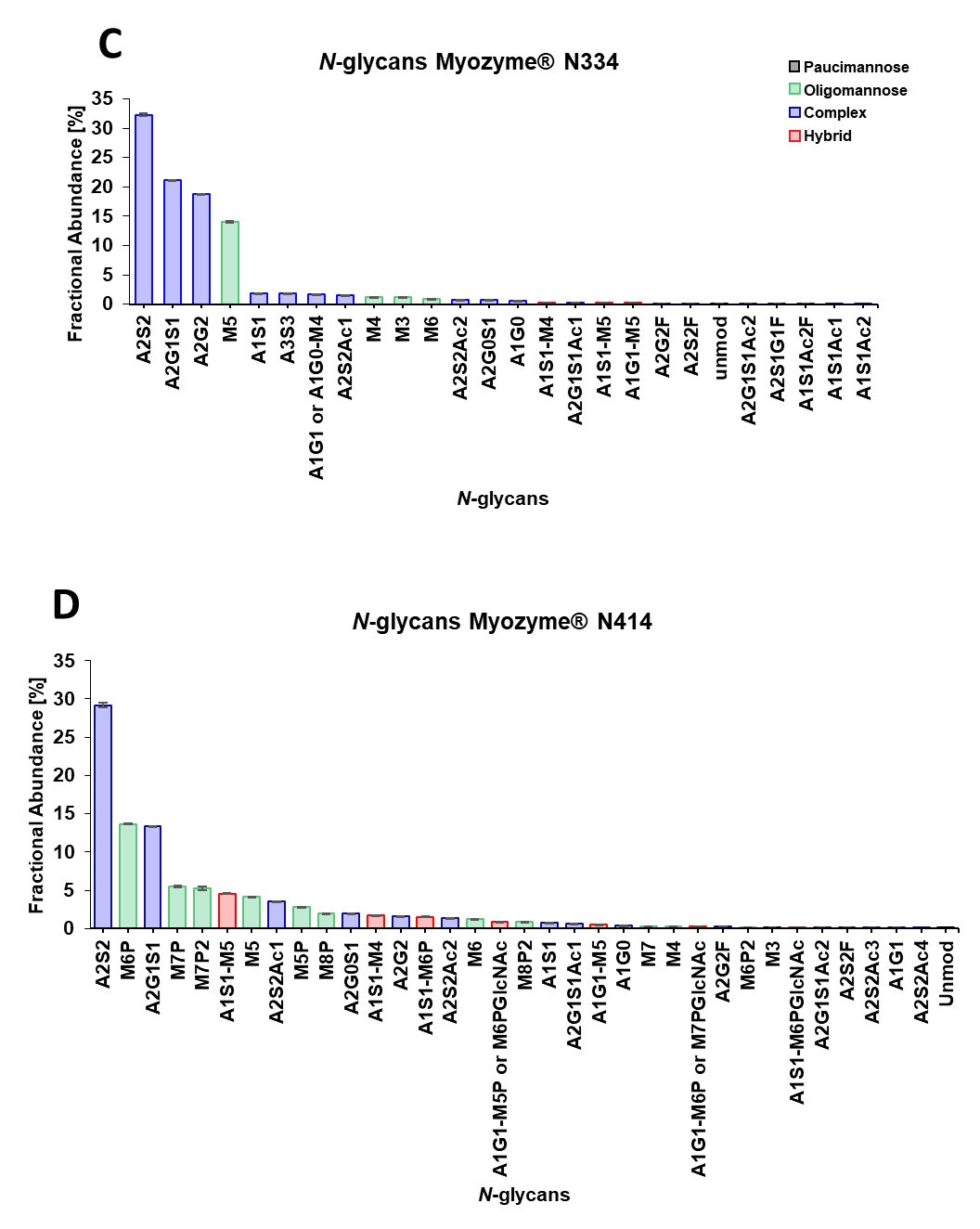


**Fig. S5** Continued.

**Fig. S5** Continued.

**Fig. S5** Continued.


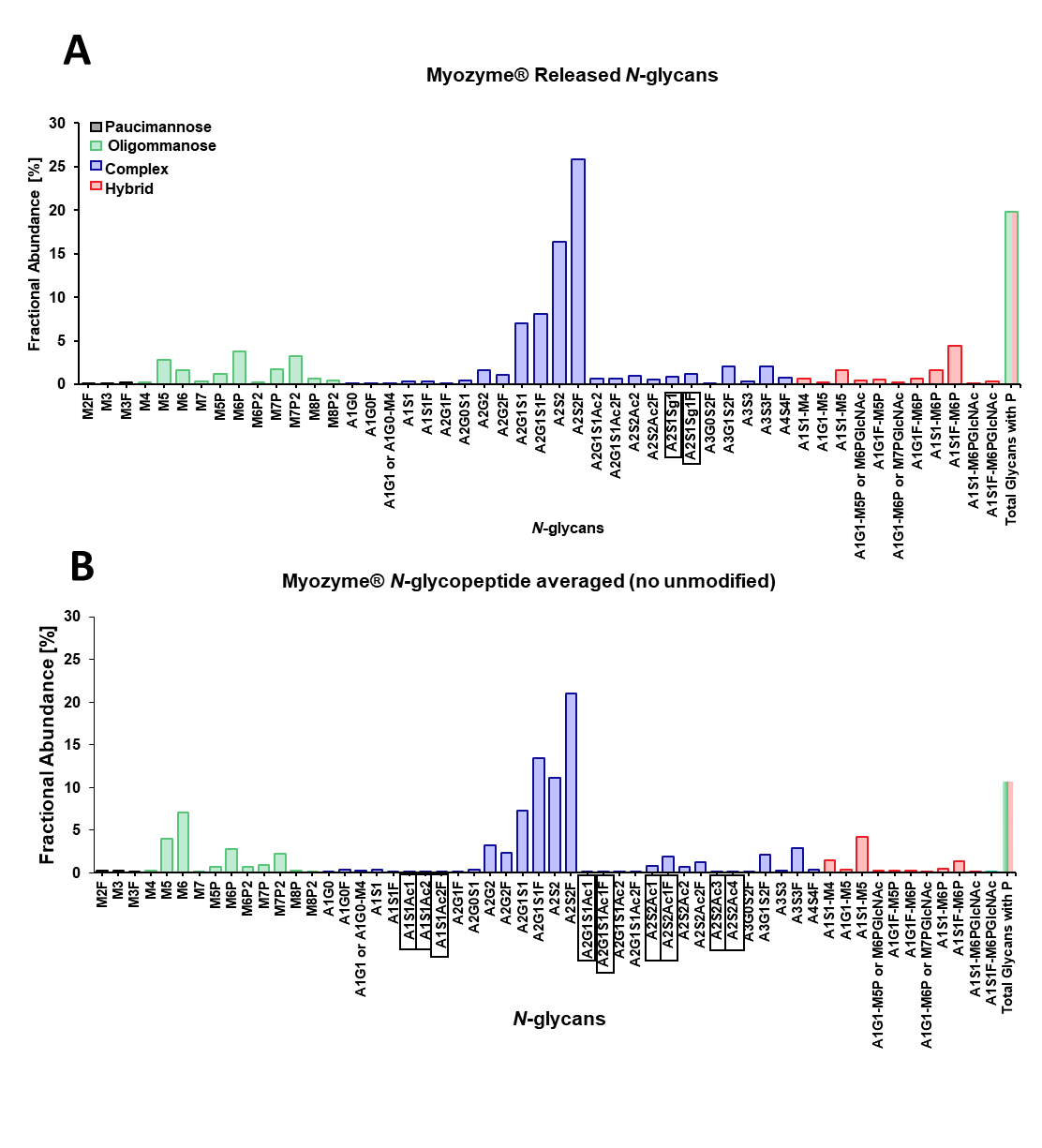


**Fig. S6** Fractional abundances and the N-glycans identified by (A) released glycan analysis and (B) glycopeptide analysis, where the fractional abundances of the glycopeptides are averaged for the 7 N-glycosylation sites without considering the fractional abundance of the unmodified peptides. The bar labelled “Total Glycans with P” indicates the summed fractional abundance of the glycan containing one or two phosphoryl group. The two glycans containing N-glycolyl neuraminic acid (Sg) in (A), indicated by a box, were excluded for the glycopeptide identification because of the problem of coelution and isomericity with one N-acetyl neuraminic acid (S) and one oxidation that makes the semi-quantitation at glycopeptide level impossible at MS^1^ level. In (B) the structures indicated by a box are the acetylated N-glycan structures found at glycopeptide level and added manually in the glycan library used for glycopeptide identification because they were not found at released glycan level but they were reported in a previous paper^1^ (see Table S1).

**Fig. S7** SAX-HPLC-MS analysis of intact glycoforms of r-hGAA. (A) In grey, the total ion current chromatogram (TICC) is shown. The retention time windows of the raw spectra reported below (B-G) are indicated in pink. More chromatographic retention is correlated with an increased sialylation degree of the glycoforms. The spanned elution of the glycoforms reduced spectra complexity allowing the acquisition of native mass spectra of r-hGAA glycoforms.

**Fig. S8** Comparison between the raw mass spectrum of intact r-hGAA glycoforms obtained by (A) a novel semi-automated native SAX-HPLC-MS approach and (B) a conventional native MS analysis with direct injection of the sample through a static nano-ESI source.

**Fig. S9** RP-HPLC-MS analysis of N-deglycosylated r-hGAA by PNGase F under non-reducing conditions. (A) Total ion current chromatogram showing the separation between PNGase F and N-deglycosylated r-hGAA that elutes in a retention time window from 31 to 35 min in two chromatographic peaks indicated in pink. (B) Raw mass spectrum associated to the t_R_ window from 31.31 to 32.22 min and corresponding deconvoluted mass spectrum. (C) Raw mass spectrum associated to the t_R_ window from 32.26 to 33.12 min and corresponding deconvoluted mass spectrum. (D) Averaged raw mass spectrum associated to the two chromatographic peaks with a t_R_ window from 31.00 to 35.00 min and associated deconvoluted mass spectrum. As it can be observed, the spectra associated to the two chromatographic peaks varies just for the charge state distribution and not for the deconvoluted mass. This may indicate that two different conformations of r-hGAA are present, probably due to different disulfide bridge linkages. The experimental mass of 99464.9 Da obtained resulted to be ≈ 102 Da heavier than the expected theoretical mass (99366.8 Da) indicating the presence of PTMs on the protein backbone or sequence variants. These modifications were attributed to cysteinylation and N-terminal pyroglutamate formation based on the results of complementary analyses (see Figure S10, S11 and S12).

**Fig. S10** Nano-RP-HPLC-MS/MS analysis of r-hGAA peptides. (A) Extracted ion current chromatogram of the ion 518.7700 corresponding to the doubly charged ion of the N-terminal peptide of r-hGAA where the N-terminal glutamine cyclizes to form N-terminal pyro-glutamate. (B) CID-MS^2^ raw mass spectrum showing the fragments y and b confirming the cyclization of glutamine to pyro-glutamate on the N-terminus of the protein. The peptide sequence is indicated and the N-terminal glutamine affected by the modification is highlighted in red.

**Fig. S11** RP-HPLC-MS analysis of N-deglycosylated r-hGAA with PNGase F under reducing conditions. (A) Total ion current chromatogram of N-deglycosylated r-hGAA that elutes in a retention time window from 30 to 34 min in one chromatographic peak indicated in pink. (B) Raw mass spectrum associated to the RT window from 29.51 to 34.01 min and corresponding deconvoluted mass spectrum. The mass of 99359.5 Da obtained for N-deglycosylated r-hGAA is 105.4 Da lighter compared to the 99464.9 Da obtained when performing enzymatic dissection under non-reducing condition (see Fig. S9). This mass shift of 105.4 Da confirmed the presence of a cysteinylation on the protein backbone because this Δm is in accordance with the reduction of a cysteinylated cysteine (-119 Da) and the 6 disulphide bridges (+12 Da) due to the reducing condition used. Together with cysteinylation N-terminal pyro-glutamate formation was unravelled and confirmed at peptide level (see Fig.10).

**Fig. S12** Micro-RP-HPLC-MS/MS analysis of r-hGAA non-reduced tryptic peptides after treatment with PNGase F. (A) Extracted ion current chromatogram of the ion 823.6515 corresponding to the doubly charged ion of r-hGAA peptide containing a cysteinylated cysteine that is also the one not involved in disulphide bridges (C318) (B) Raw mass spectrum of the doubly charge peptide containing the cysteinylated cysteine associated to the RT 48.49 min, (B) CID-MS^2^ raw mass spectrum showing the fragments y and b of the doubly charge peptide confirming the cysteinylation of C318. The peptide sequence is indicated and the cysteine affected by the modification is highlighted in red (C318). Notably, even if C318 was the one most affected by cysteinylation also cysteines 26, 36, 47 and 882 displayed cysteinylation at peptide level, albeit at a comparably low percentage (data not shown).

**Fig. S13** SAX-HPLC-MS analysis of desialylated glycoforms of r-hGAA. (A) Total ion current chromatogram of desialylated r-hGAA obtained by SAX-HPLC-MS. (B-E) Raw mass spectra associated to the retention time window indicated in the figure.

**Fig. S14** Mirror plot of the deconvoluted mass spectra of desialylated (orange) and intact (blue) r-hGAA. The shift to lower masses of desialylated (108 to 113.5 Da) compared to the intact masses of r-hGAA glycoforms (109 to 118 kDa), together with the absence of Δm of 291 (the mass increment of a sialic acid residue) between mass peaks, indicated the completeness of the enzymatic dissection.

**Fig. S15** Filtering of r-hGAA intact glycoform annotations using the bioinformatics workflow reported in Fig. 5. (A) Mirror plot of experimentally and in-silico desialylated spectra without any filtering of the intact glycoform MoFi annotations. (B) Mirror plot of experimentally and in-silico desialylated spectra filtered based on the mass fitting between experimental and computational data. (C) Mirror plot of experimentally and in-silico desialylated spectra filtered based on the mass fitting between experimental and computational data and based on the hit score cutoff of 0.01%. Even if the filtered (by mass and 0.01% hit score cutoff) and the experimental spectra do not perfectly fit, an improvement of the merge of experimental and in-silico data between non-filtered and filtered spectra can be clearly observed.

**References**

1. H. Park, S. You, J. I. J. Kim, W. Kim, J. Do, Y. Jang, D. Kim, J. Lee, J. Ha, D. B. Oh, J. I. J. Kim and H. H. Kim, J. Pharm. Biomed. Anal., 2019, 169, 188–195.
