# Supplementary material for: Glycoproteomics of a Single Protein: Revealing Tens of Thousands of Myozyme Glycoforms by Hybrid HPLC-MS Approaches
Source: Mol Cell Proteomics. 2023 Jul 20;22(9):100622. doi: 10.1016/j.mcpro.2023.100622 (PMC10470421; doi:10.1016/j.mcpro.2023.100622)

Supplementary information 2

Glycoproteomics of a single protein: revealing tens of thousands of Myozyme® glycoforms by hybrid HPLC-MS approaches

Fiammetta Di Marco[a,b], Constantin Blöchl[a,c], Wolfgang Esser-Skala[b,d], Veronika Schäpertöns[a,d], Tao Zhang[c], Manfred Wuhrer[c], Koen Sandra[e] , Therese Wohlschlager[a,b], Christian G. Huber[a,b]

a. Department of Biosciences and Medical Biology, Bioanalytical Research Labs, University of Salzburg, Hellbrunner Straße 34, 5020 Salzburg, Austria
b. Christian Doppler Laboratory for Innovative Tools for Biosimilar Characterization, University of Salzburg, Hellbrunner Straße 34, 5020 Salzburg, Austria
c. Center for Proteomics and Metabolomics, Leiden University Medical Center, Albinusdreef 2, 2333 ZA Leiden, The Netherlands
d. Department of Biosciences and Medical Biology, Computational Systems Biology Group, University of Salzburg, Hellbrunner Straße 34, 5020 Salzburg, Austria
e. Research Institute for Chromatography (RIC), President Kennedypark 26, 8500 Kortrijk, Belgium

* *Correspondence:* *c.huber@sbg.ac.at, c.blochl@lumc.nl, m.wuhrer@lumc.nl, t.zhang@lumc.nl*

Glycan 1

M2F (H2N2F1)

**Monoisotopic mass:**	**896.35 Da**	**UniCarb-DB: #2223**
**Charge observed:**	**1-**	
**Theoretical ion:**	***m/z* 895.34**	
**Observed ion:**	***m/z* 895.45**	
**Mass deviation:**	***m/z* 0.11**	
**Retention time:**	**58.6 min**	

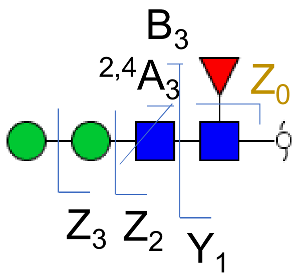

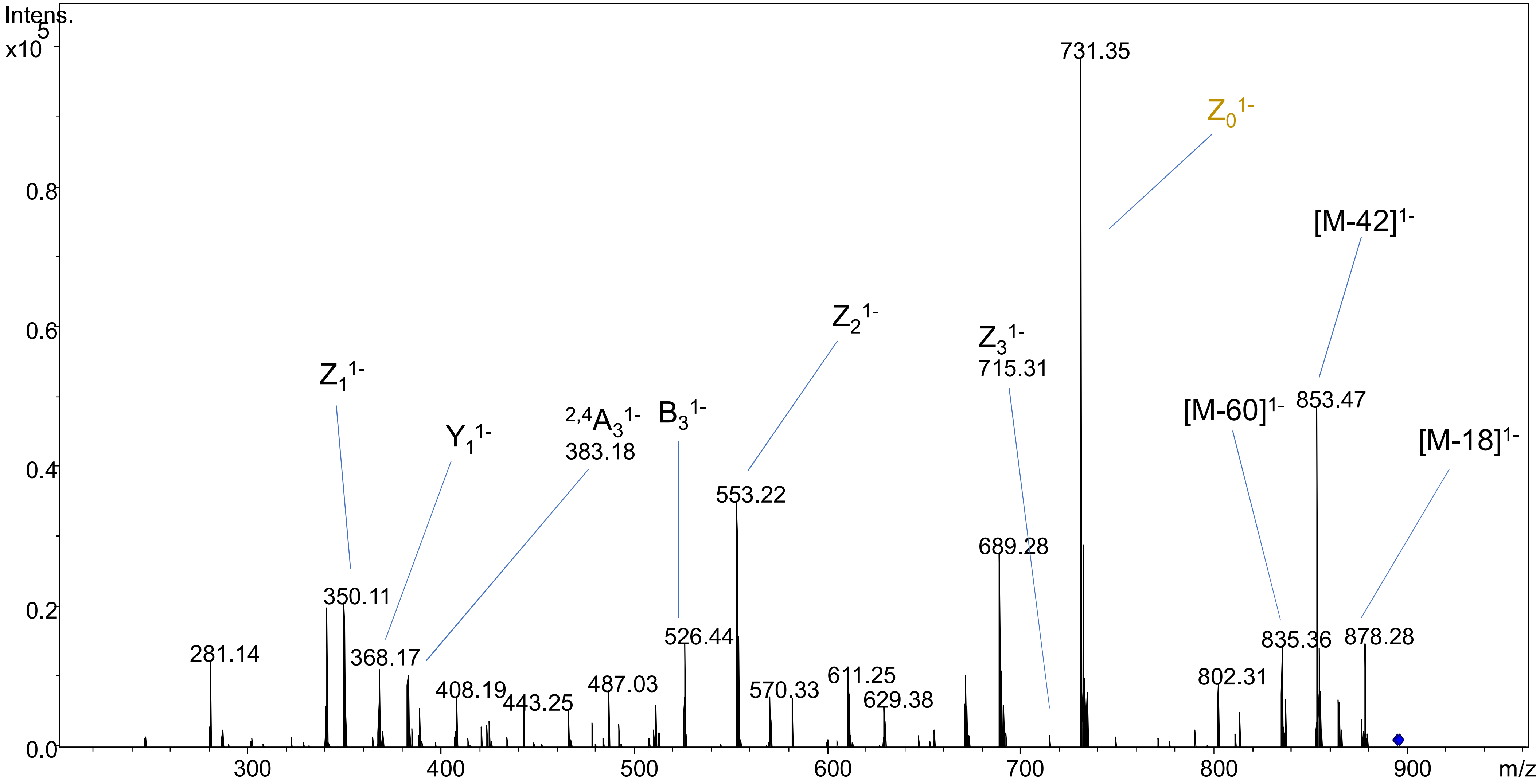

Glycan 2

M3 (H3N2)

**Monoisotopic mass:**	**912.35 Da**	**UniCarb-DB: #610**
**Charge observed:**	**1-**	
**Theoretical ion:**	***m/z* 911.34**	
**Observed ion:**	***m/z* 911.44**	
**Mass deviation:**	***m/z* 0.10**	
**Retention time:**	**55.5 min**	

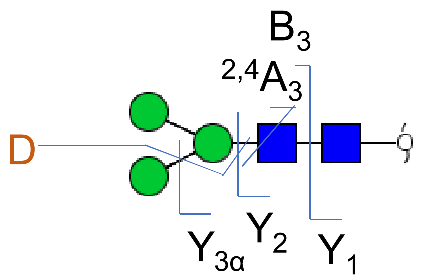

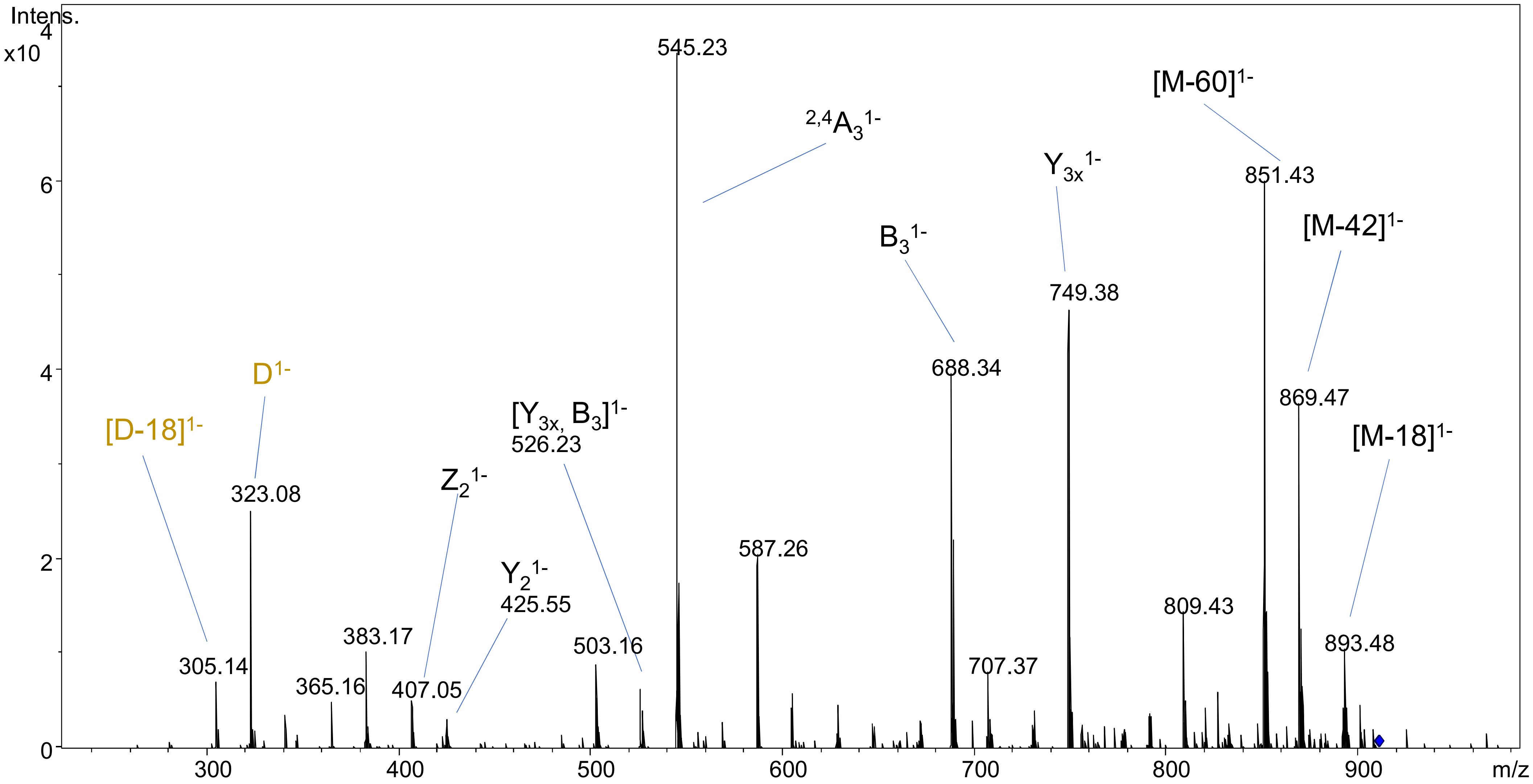

Glycan 3

M3F (H3N2F1)

**Monoisotopic mass:**	**1058.40 Da**
**Charge observed:**	**1-**
**Theoretical ion:**	***m/z* 1057.39**
**Observed ion:**	***m/z* 1057.53**
**Mass deviation:**	***m/z* 0.14**
**Retention time:**	**66.4 min**

UniCarb-DB: #441

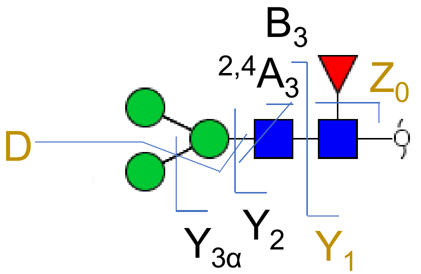

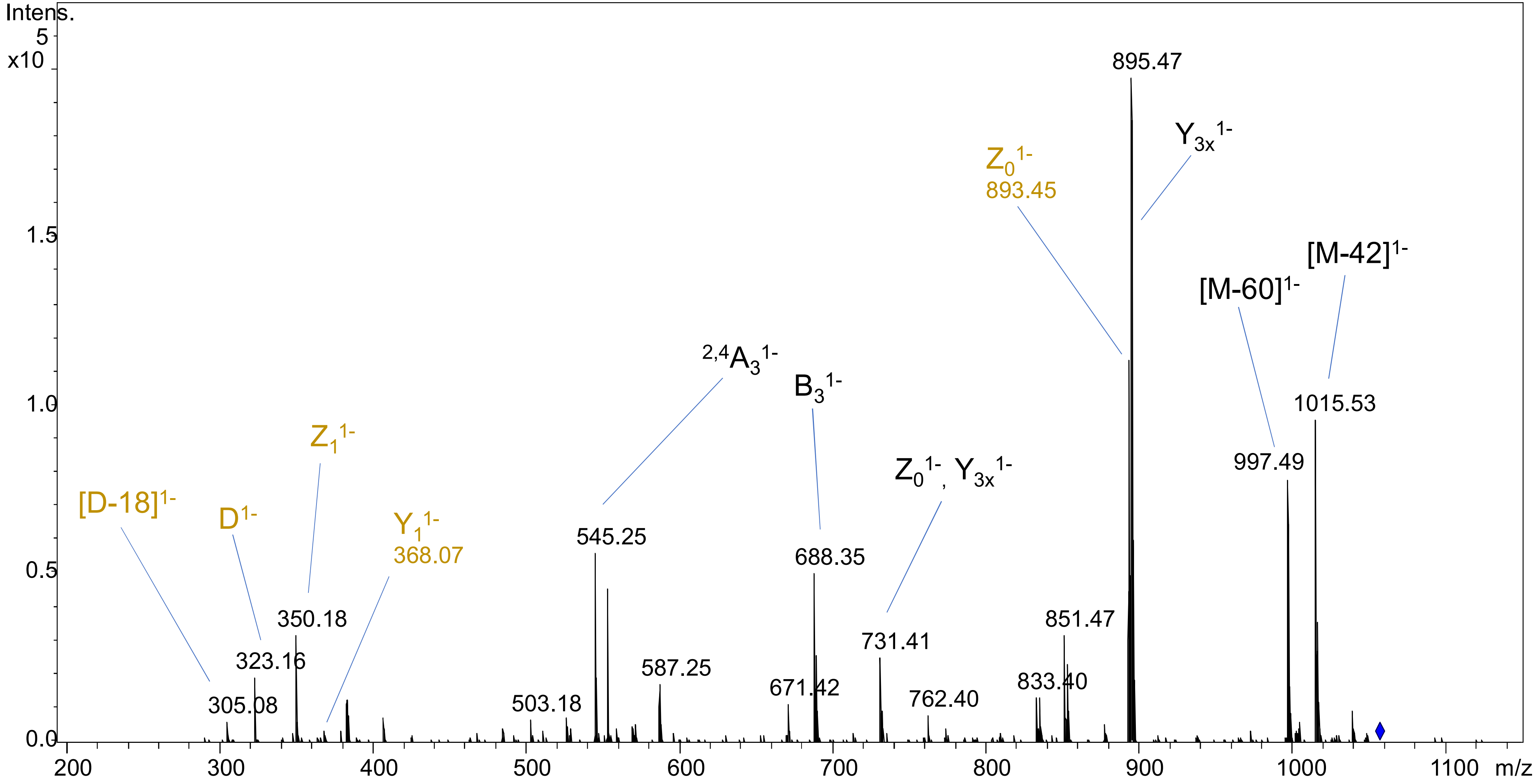

Glycan 4

M4 (H4N2)

**Monoisotopic mass:**	**1074.40 Da**
**Charge observed:**	**1-**
**Theoretical ion:**	***m/z* 1073.39**
**Observed ion:**	***m/z* 1073.53**
**Mass deviation:**	***m/z* 0.14**
**Retention time:**	**52.2 min**

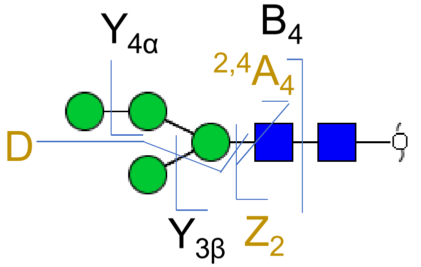

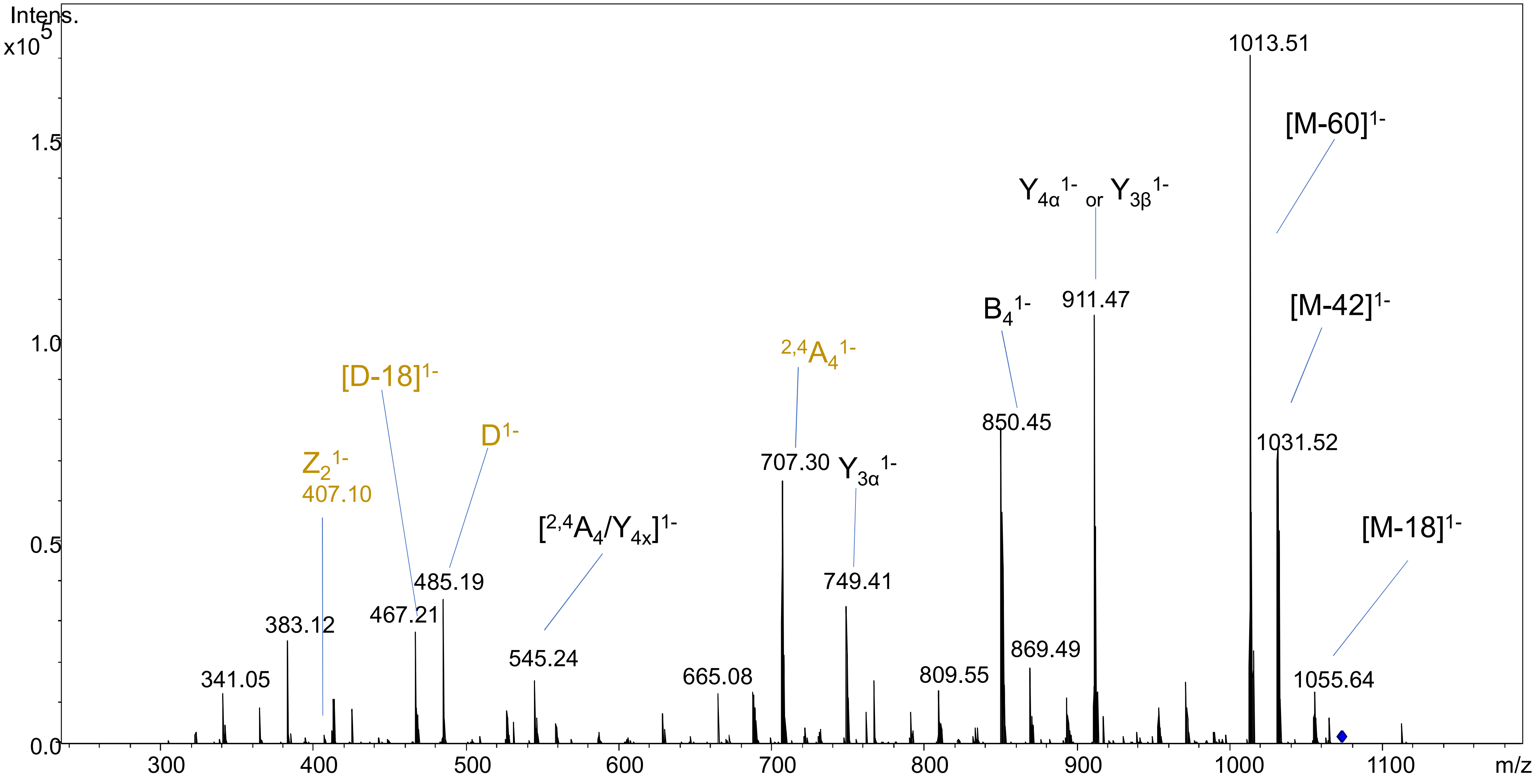

Glycan 5a

M5 (H5N2)

**Monoisotopic mass:**	**1236.45 Da**
**Charge observed:**	**1-**
**Theoretical ion:**	***m/z* 1235.44**
**Observed ion:**	***m/z* 1235.57**
**Mass deviation:**	***m/z* 0.13**
**Retention time:**	**42.6 min**

UniCarb-DB: #2213

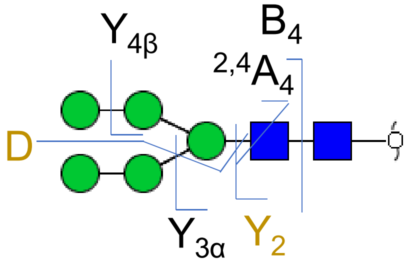

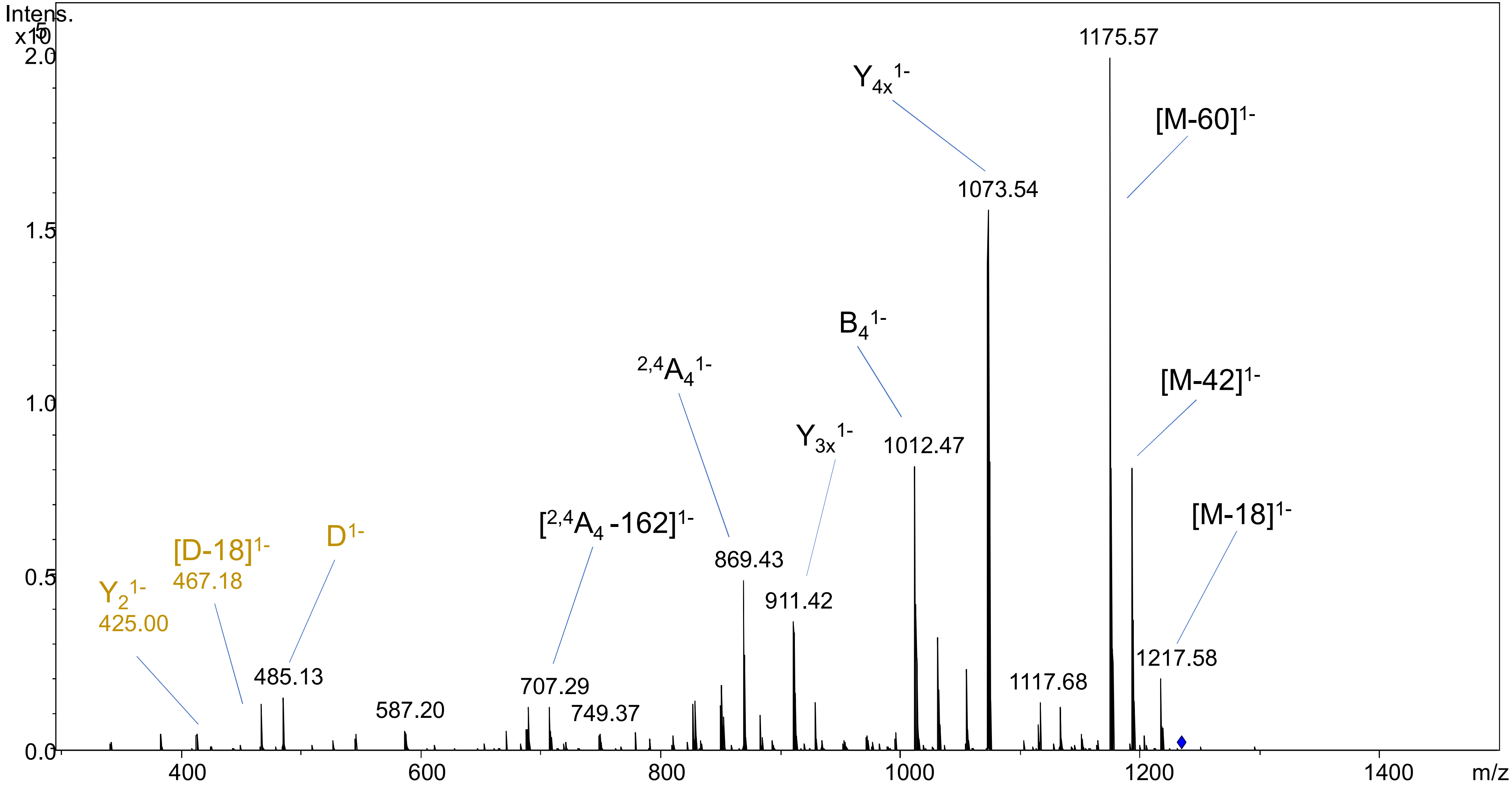

Glycan 5b

M5 (H5N2)

**Monoisotopic mass:**	**1236.45 Da**
**Charge observed:**	**1-**
**Theoretical ion:**	***m/z* 1235.44**
**Observed ion:**	***m/z* 1235.60**
**Mass deviation:**	***m/z* 0.16**
**Retention time:**	**57.6 min**

UniCarb-DB: #395

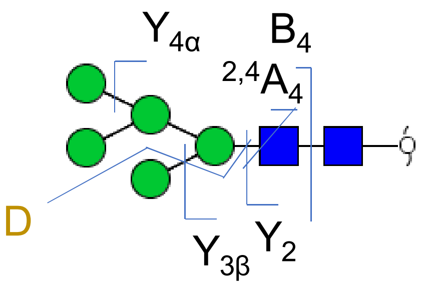

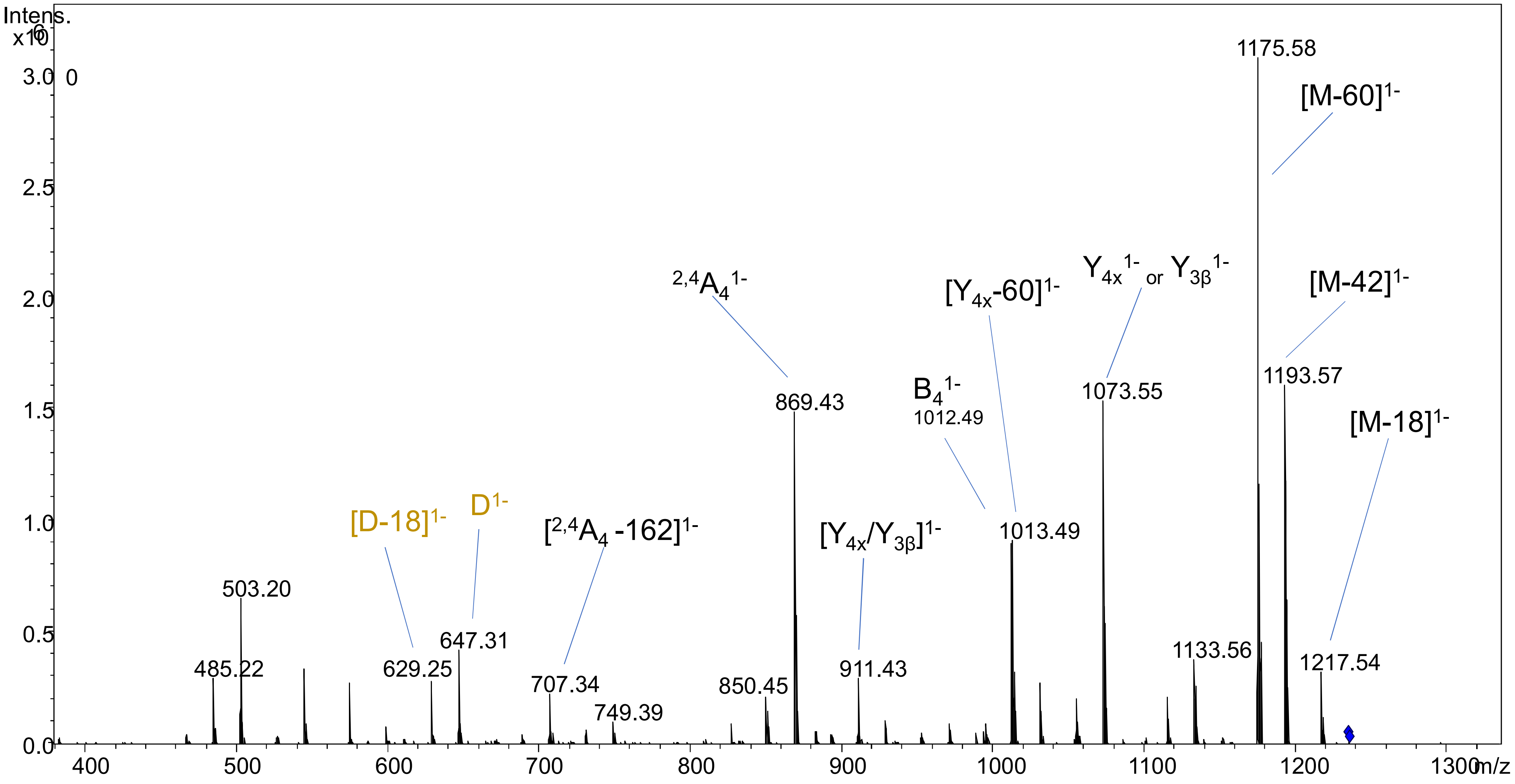

Glycan 6

M6 (H6N2)

Monoisotopic mass: **1398.51 Da**
Charge observed: **1-**
Theoretical ion: ***m/z* 1397.50**
Observed ion: ***m/z* 1397.67**
Mass deviation: ***m/z* 0.17**
Retention time: **48.4 min**

UniCarb-DB: #613

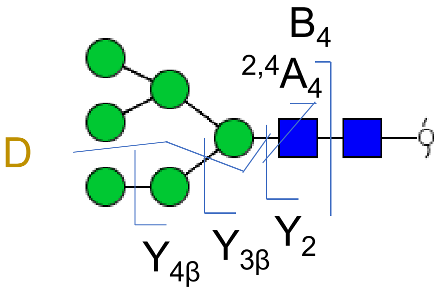

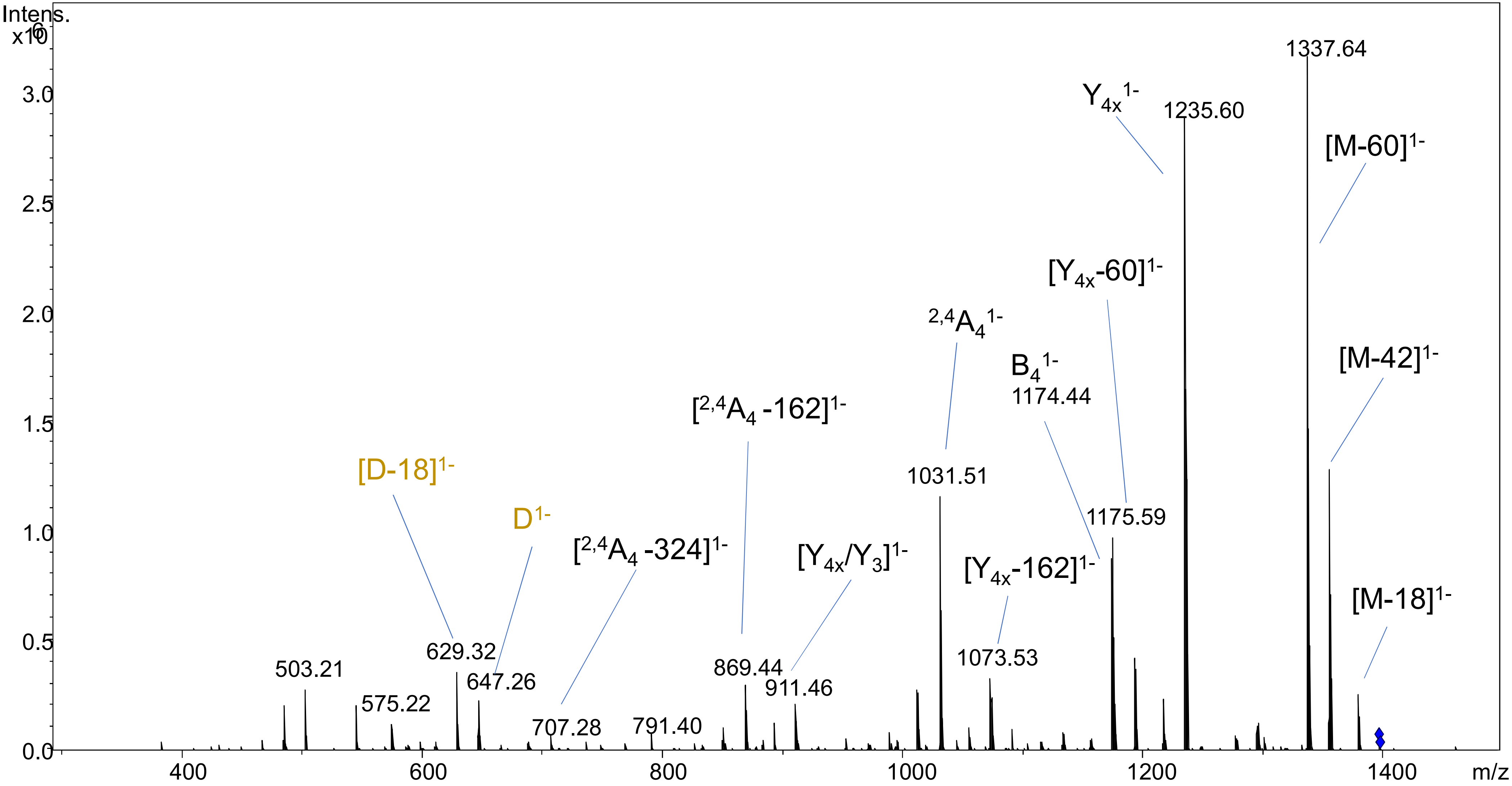

Glycan 7a

M7 (H7N2)

**Monoisotopic mass:**	**1560.55 Da**
**Charge observed:**	**2-**
**Theoretical ion:**	***m/z* 779.27**
**Observed ion:**	***m/z* 779.42**
**Mass deviation:**	***m/z* 0.15**
**Retention time:**	**46.6 min**

UniCarb-DB: #403

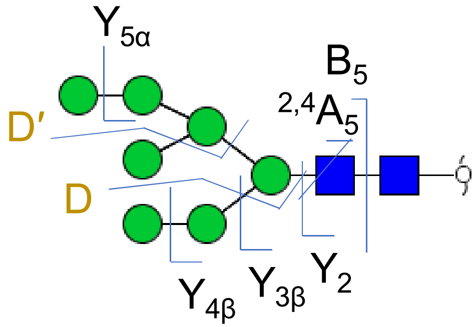

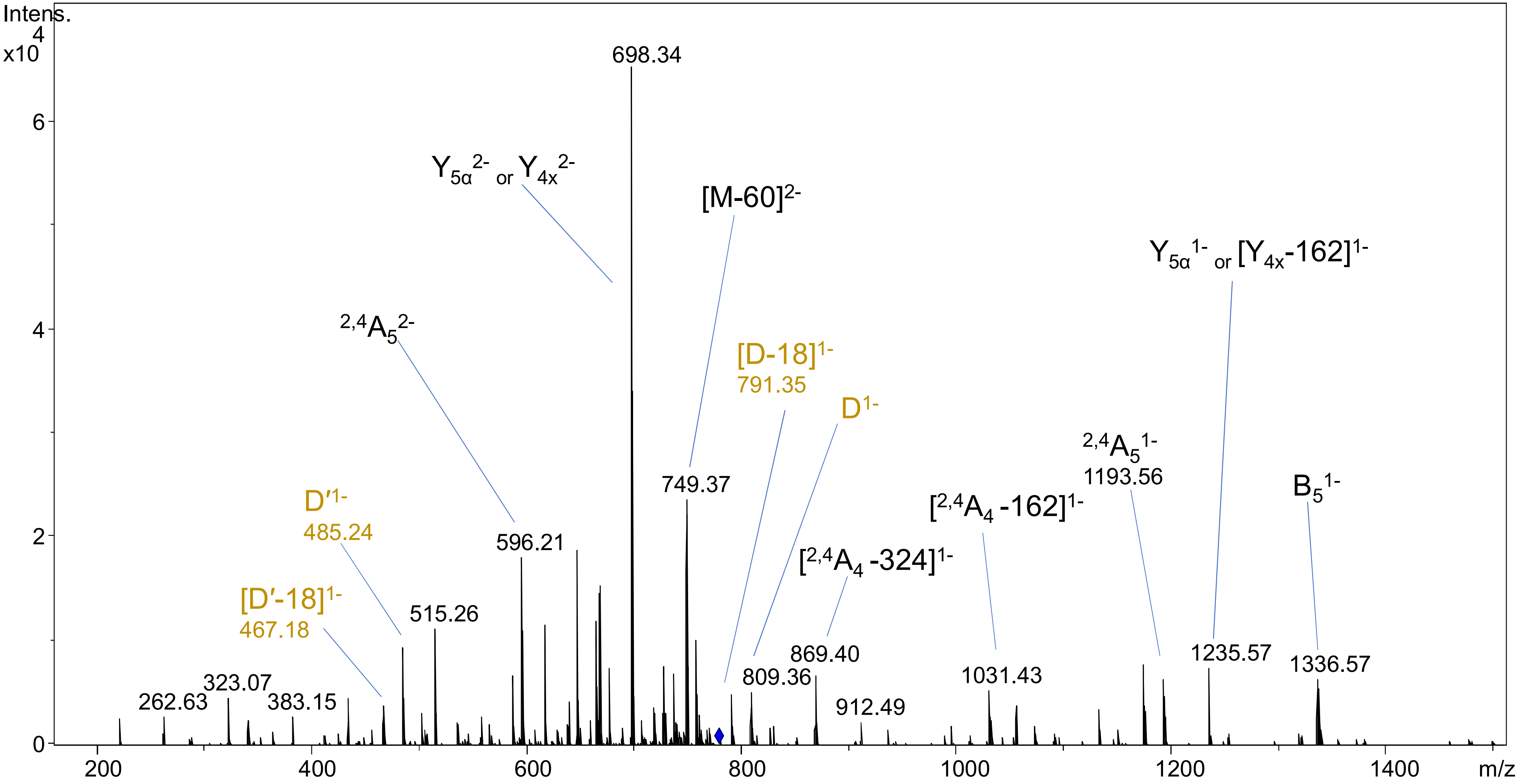

Glycan 7b

M7 (H7N2)

**Monoisotopic mass:**	**1560.55 Da**	**UniCarb-DB: #403**
**Charge observed:**	**2-**	
**Theoretical ion:**	***m/z* 779.27**	
**Observed ion:**	***m/z* 779.37**	
**Mass deviation:**	***m/z* 0.10**	
**Retention time:**	**48.8 min**	

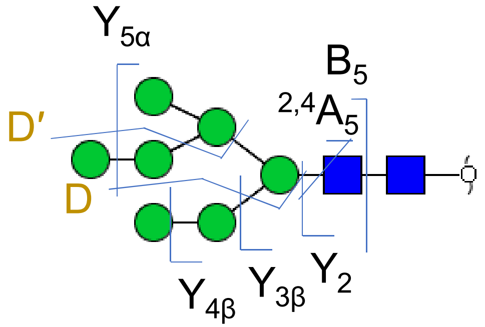

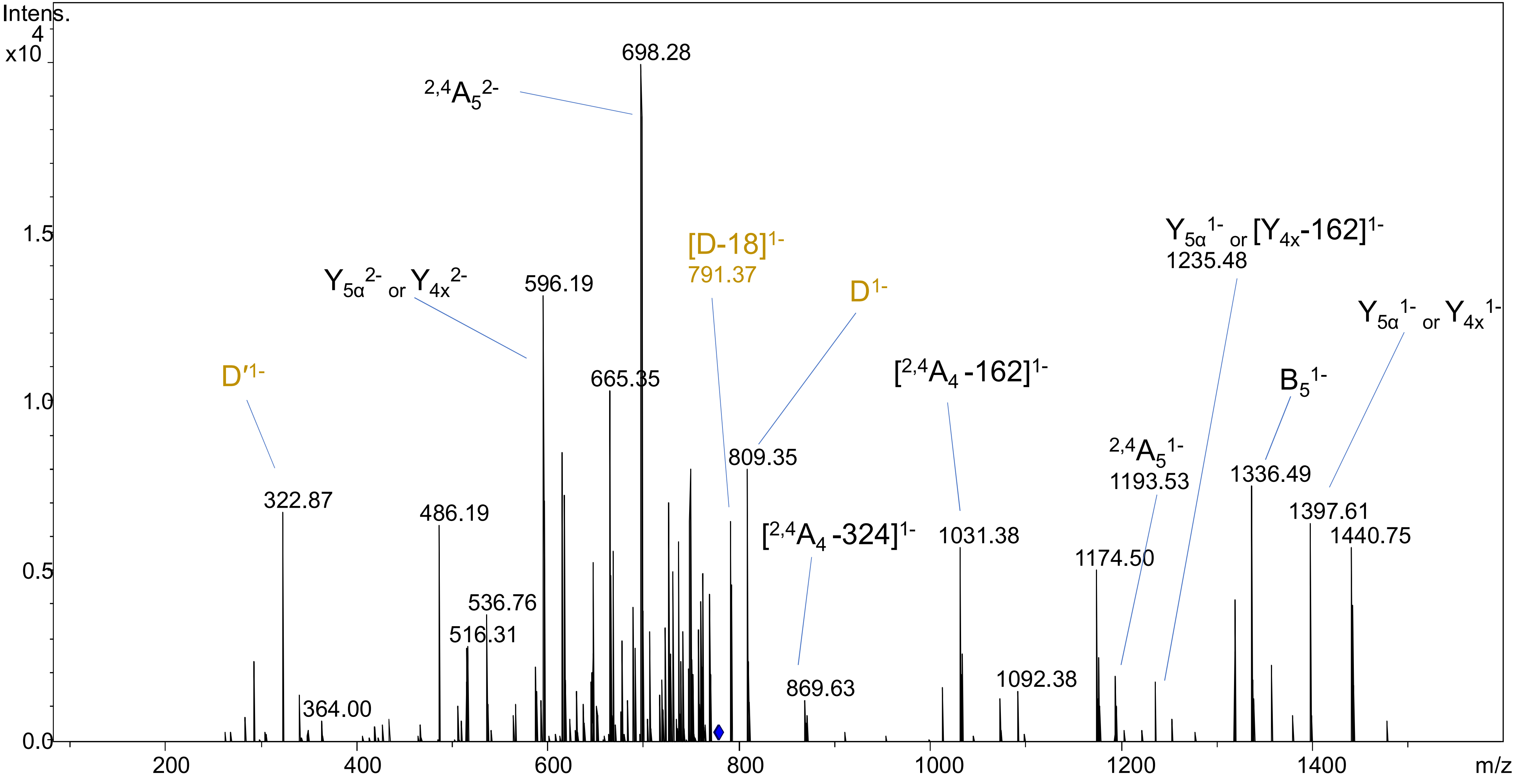

Glycan 8

M5P (H5N2P1)

**Monoisotopic mass:**	**1316.42 Da**
**Charge observed:**	**1-**
**Theoretical ion:**	***m/z* 1315.41**
**Observed ion:**	***m/z* 1315.58**
**Mass deviation:**	***m/z* 0.17**
**Retention time:**	**33.4 min**

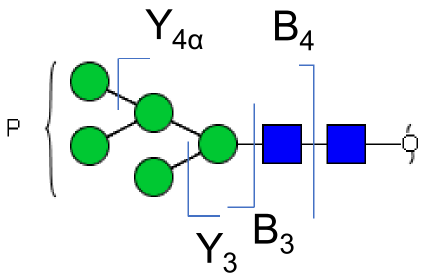

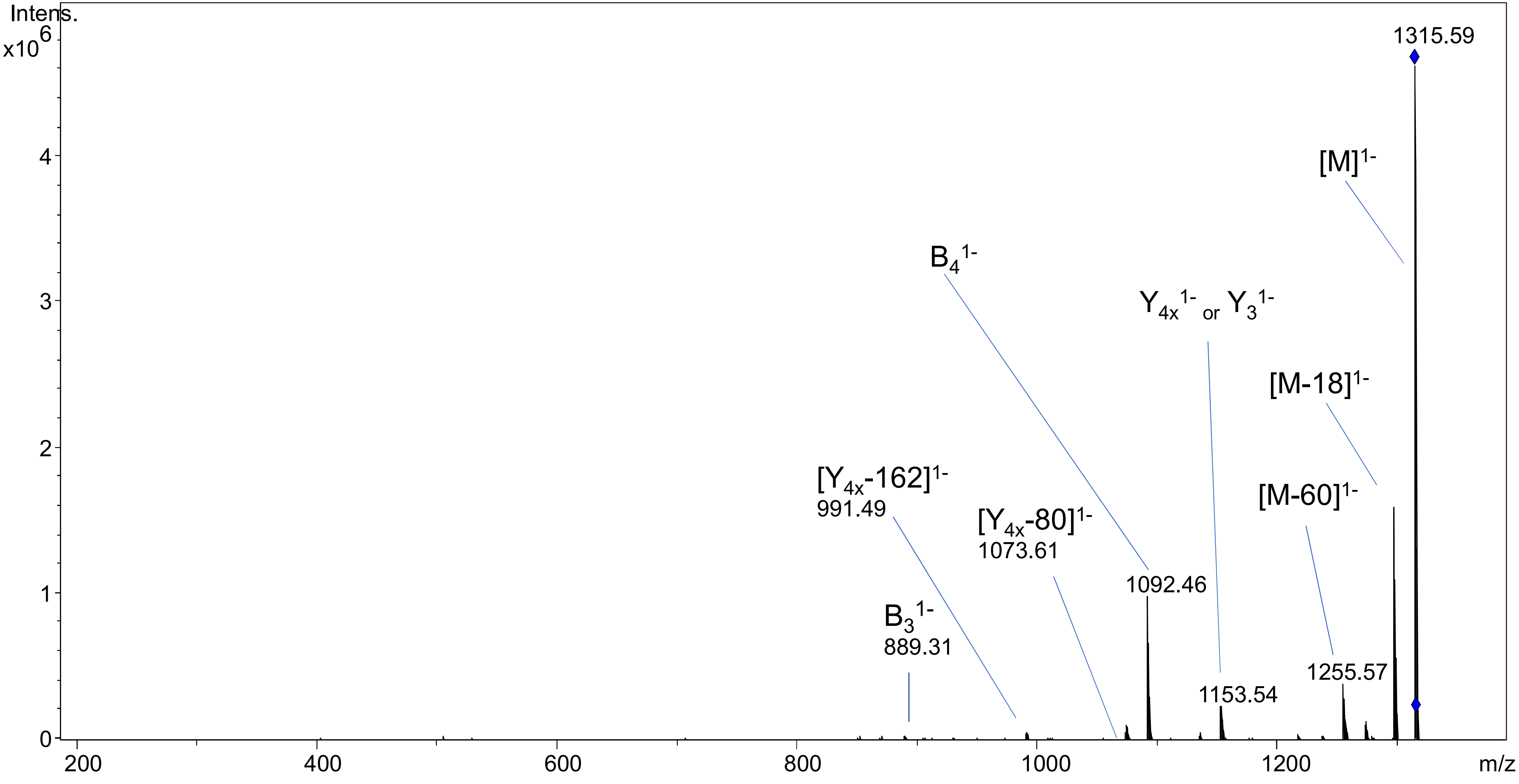

Glycan 9a

M6P (H6N2P1)

**Monoisotopic mass:**	**1478.47 Da**
**Charge observed:**	**2-**
**Theoretical ion:**	***m/z* 738.23**
**Observed ion:**	***m/z* 738.34**
**Mass deviation:**	***m/z* 0.11**
**Retention time:**	**39.1 min**

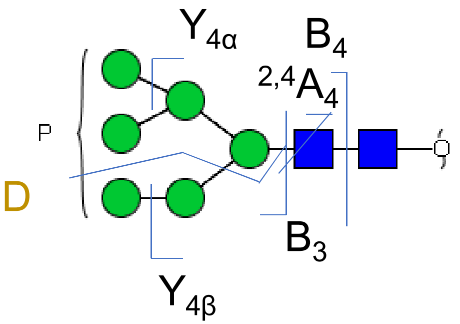

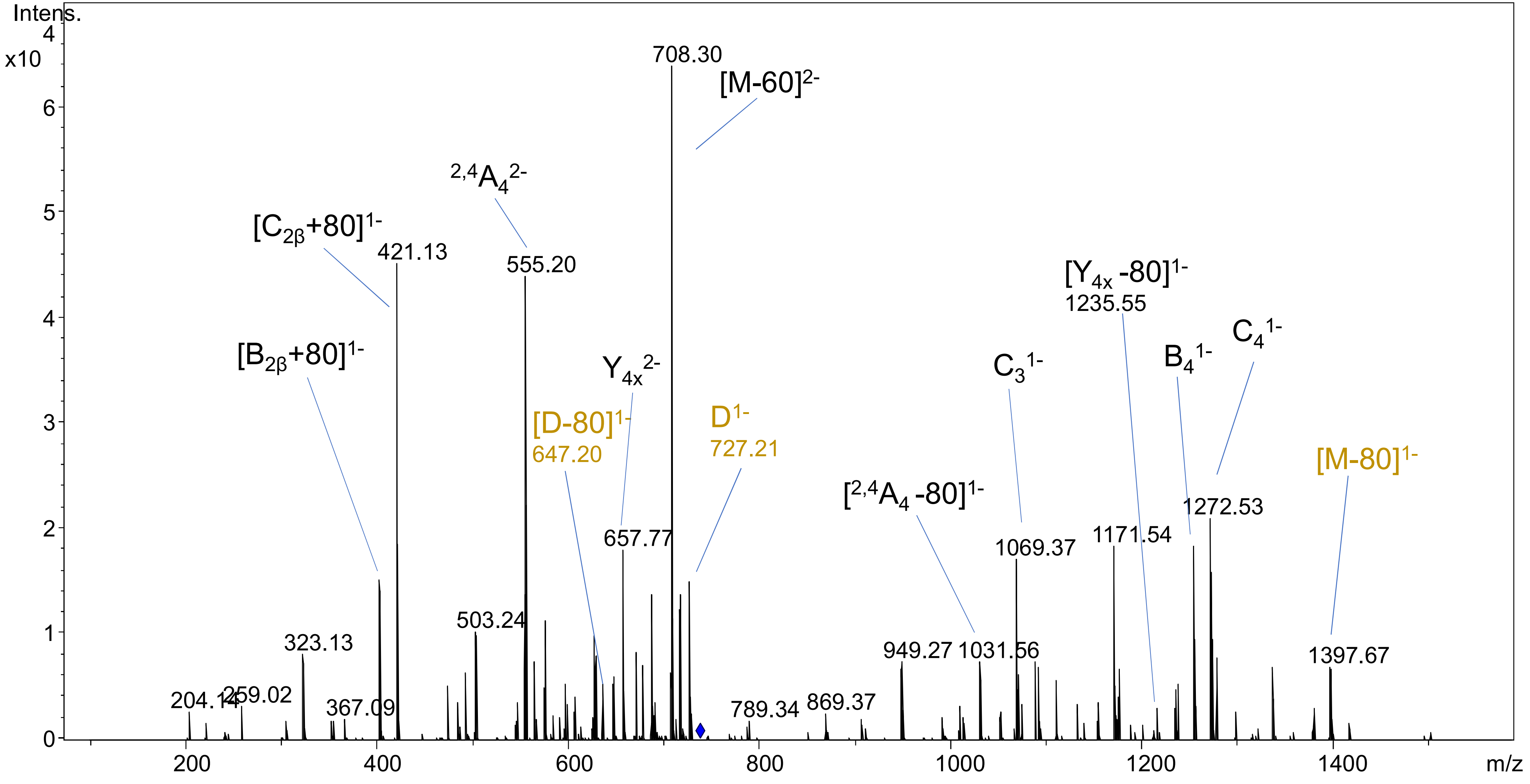

Glycan 9b

M6P (H6N2P1)

**Monoisotopic mass:**	**1478.47 Da**
**Charge observed:**	**2-**
**Theoretical ion:**	***m/z* 738.23**
**Observed ion:**	***m/z* 738.33**
**Mass deviation:**	***m/z* 0.10**
**Retention time:**	**42.2 min**

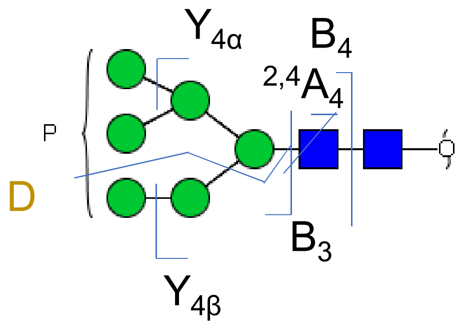

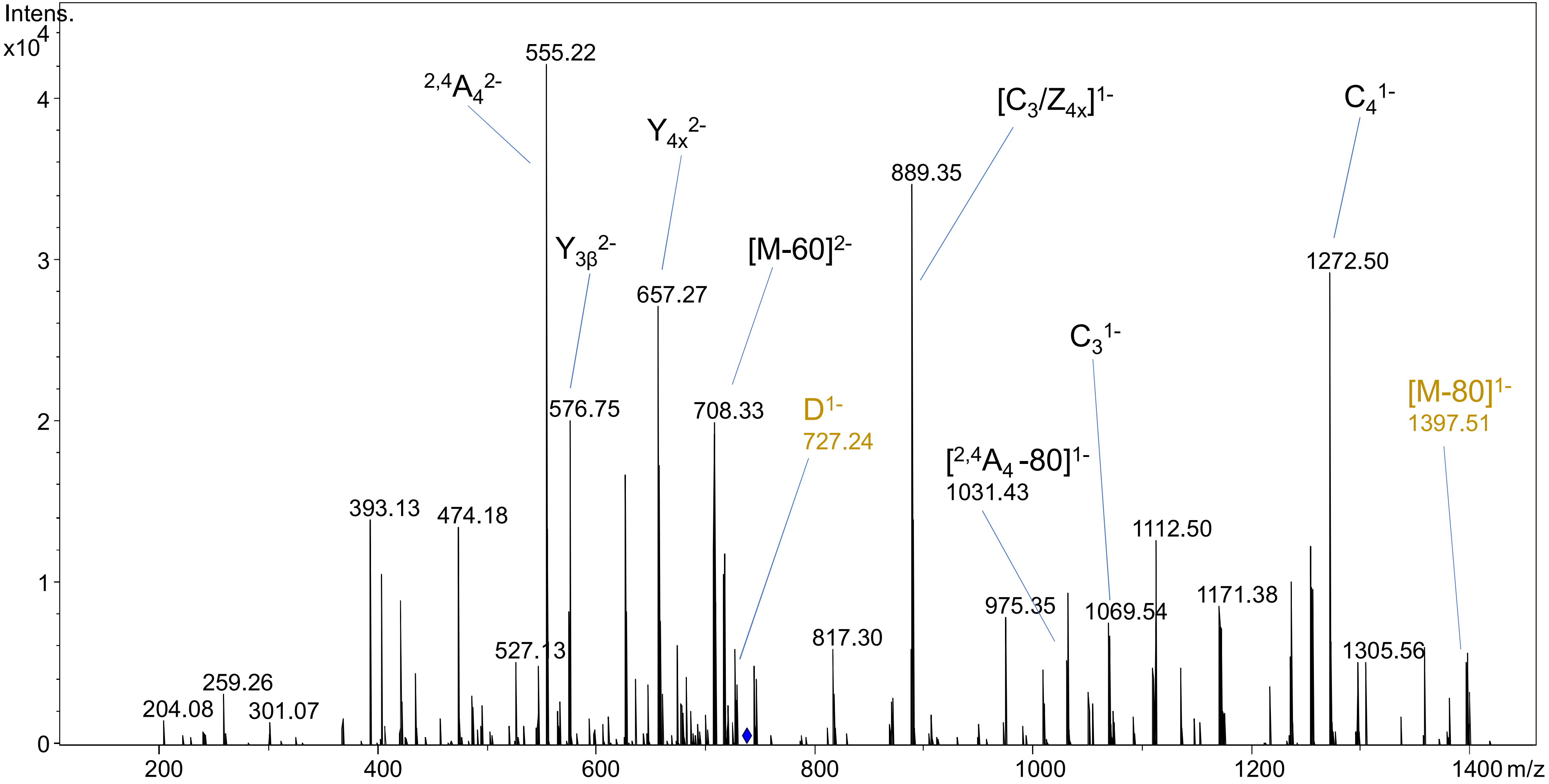

Glycan 10

M6P2 (H6N2P2)

**Monoisotopic mass:**	**1558.43 Da**
**Charge observed:**	**2-**
**Theoretical ion:**	***m/z* 778.21**
**Observed ion:**	***m/z* 778.33**
**Mass deviation:**	***m/z* 0.12**
**Retention time:**	**30.6 min**

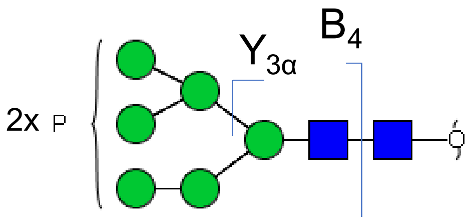

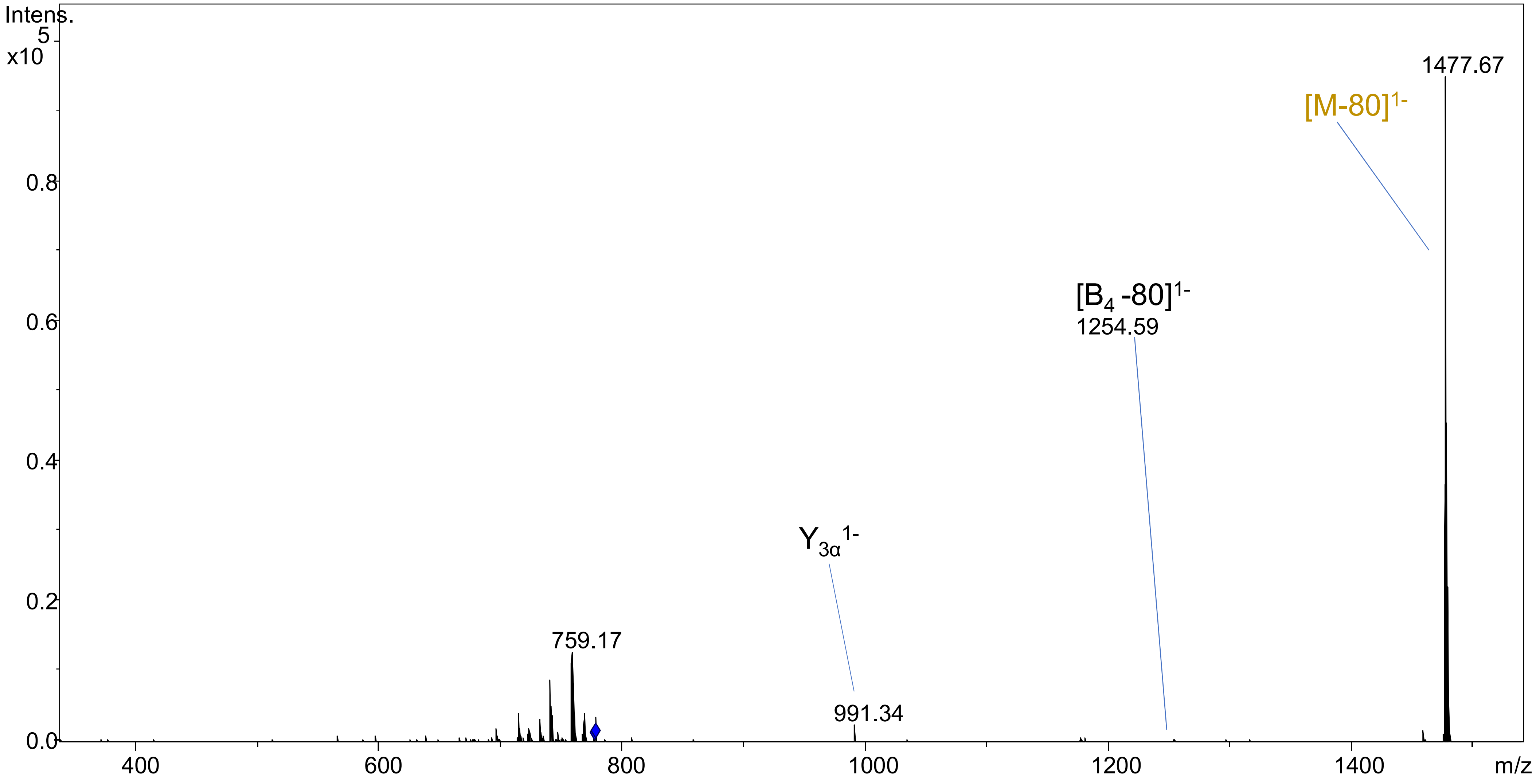

Glycan 11

M7P (H7N2P1)

**Monoisotopic mass:**	**1640.52 Da**
**Charge observed:**	**2-**
**Theoretical ion:**	***m/z* 819.25**
**Observed ion:**	***m/z* 819.28**
**Mass deviation:**	***m/z* 0.03**
**Retention time:**	**38.7 min**

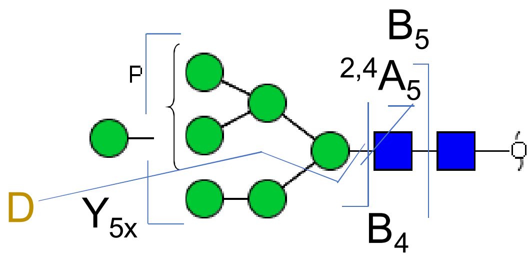

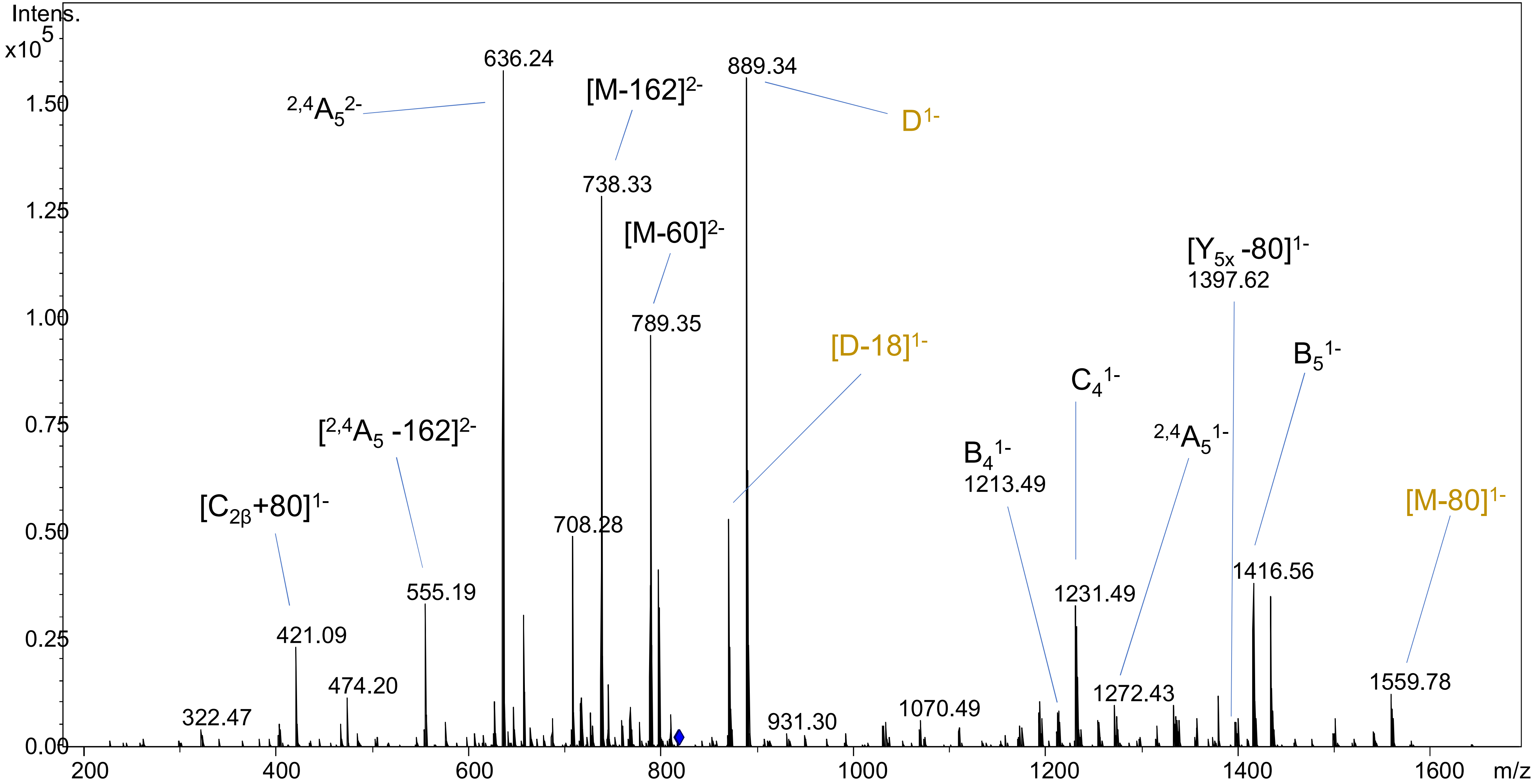

Glycan 12

M6PGlcNAc (H6N3P1)

Monoisotopic mass: 1681.55 Da
Charge observed: 2-
Theoretical ion: *m/z* 839.77
Observed ion: *m/z* 839.90
Mass deviation: *m/z* 0.13
Retention time: 41.9 min
Note: Isomer to glycan 41.

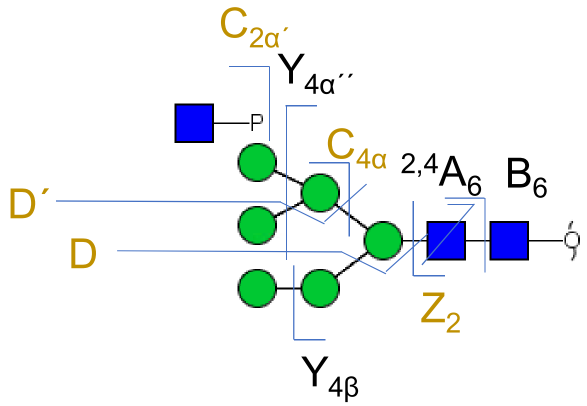

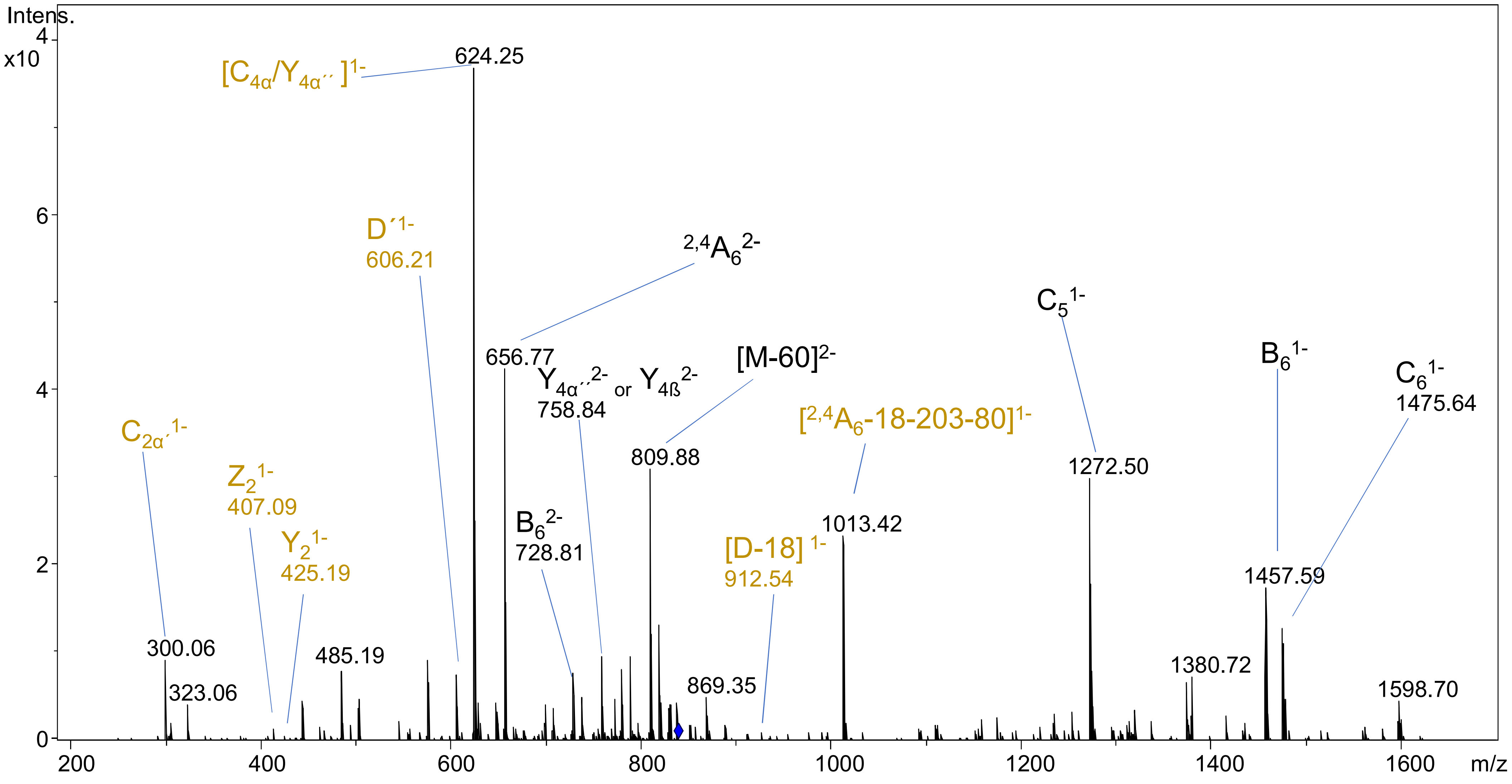

Glycan 13

M7P2
(H7N2P2)

**Monoisotopic mass:**	**1720.49 Da**
**Charge observed:**	**2-**
**Theoretical ion:**	***m/z* 859.24**
**Observed ion:**	***m/z* 859.38**
**Mass deviation:**	***m/z* 0.14**
**Retention time:**	**31.7 min**

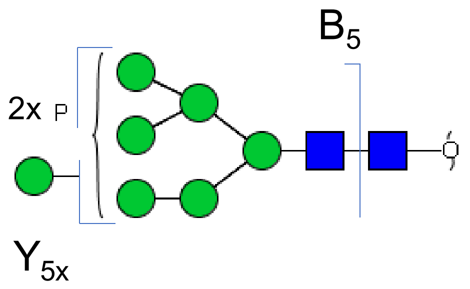

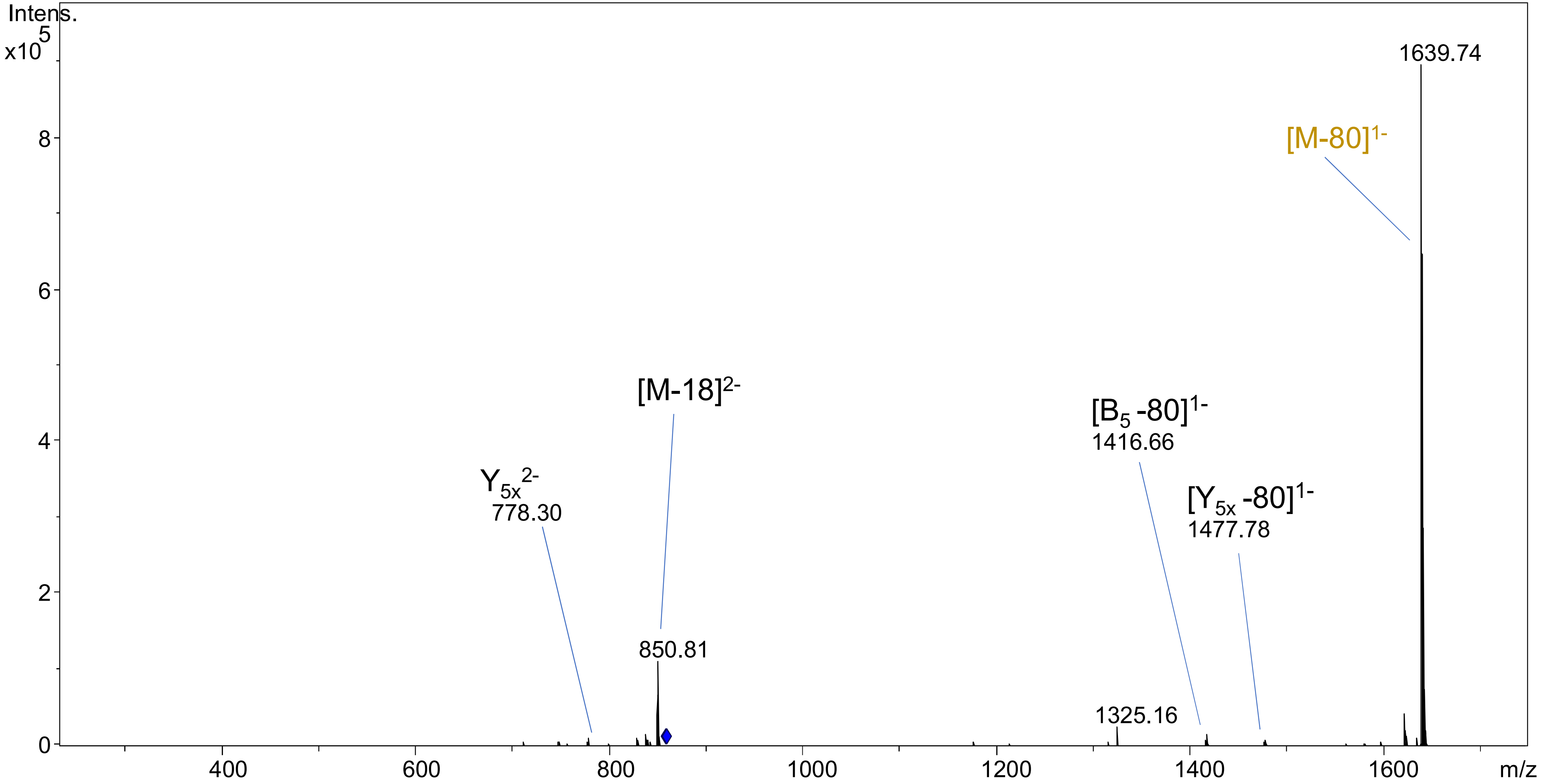

Glycan 14

M8P
(H8N2P1)

**Monoisotopic mass:**	**1802.57 Da**
**Charge observed:**	**2-**
**Theoretical ion:**	***m/z* 900.28**
**Observed ion:**	***m/z* 900.40**
**Mass deviation:**	***m/z* 0.12**
**Retention time:**	**38.6 min**

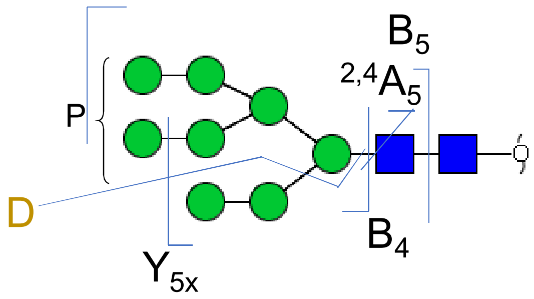

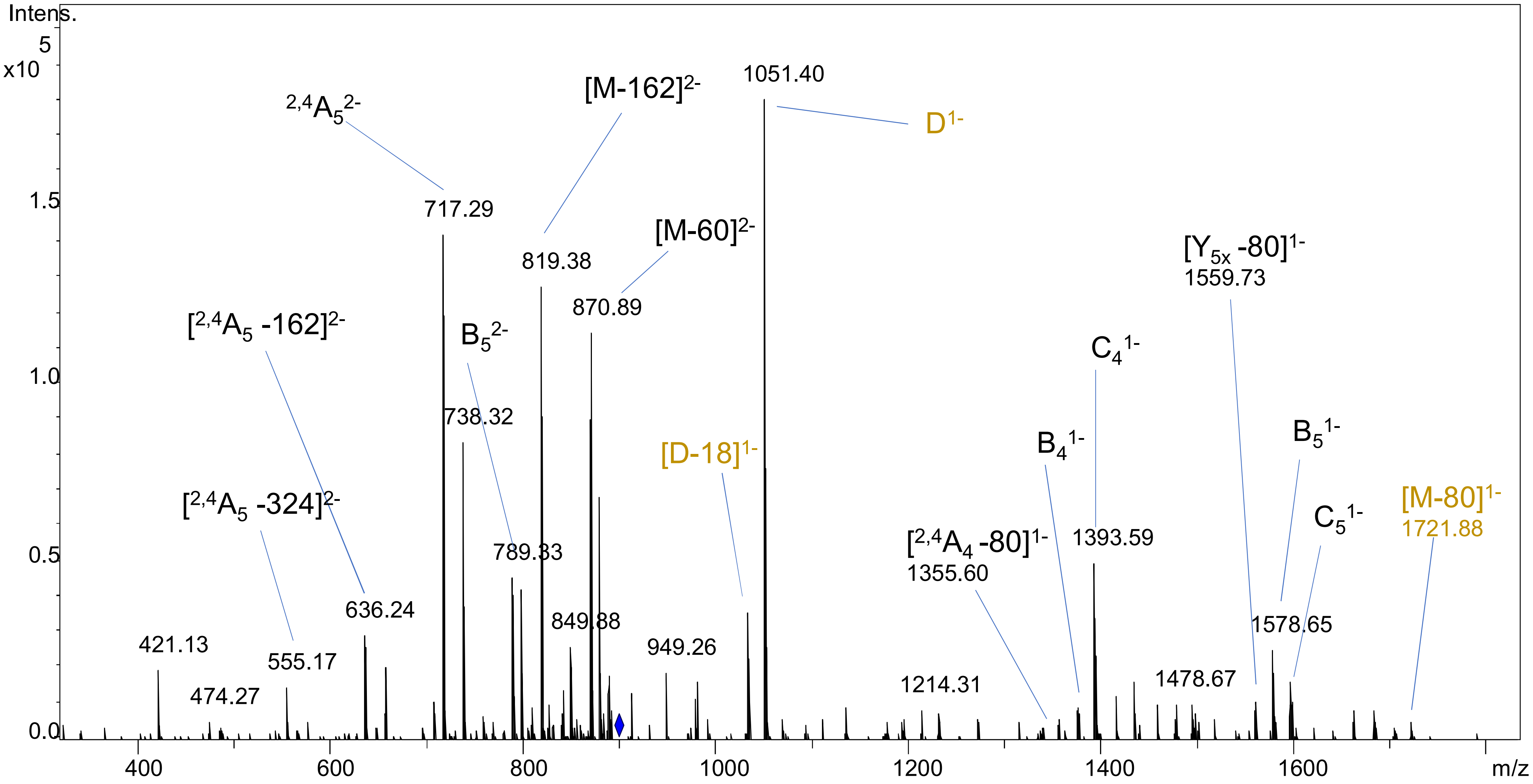

Glycan 15

M7PGlcNAc
(H7N3P1)

Monoisotopic mass: 1843.60 Da
Charge observed: 2-
Theoretical ion: *m/z* 920.79
Observed ion: *m/z* 920.91
Mass deviation: *m/z* 0.12
Retention time: 42.4 min
Note: Isomer to glycan 44.

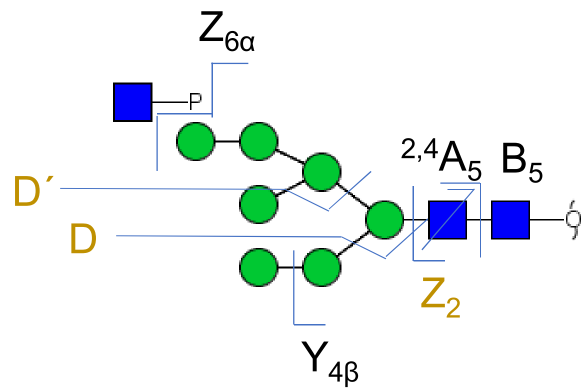

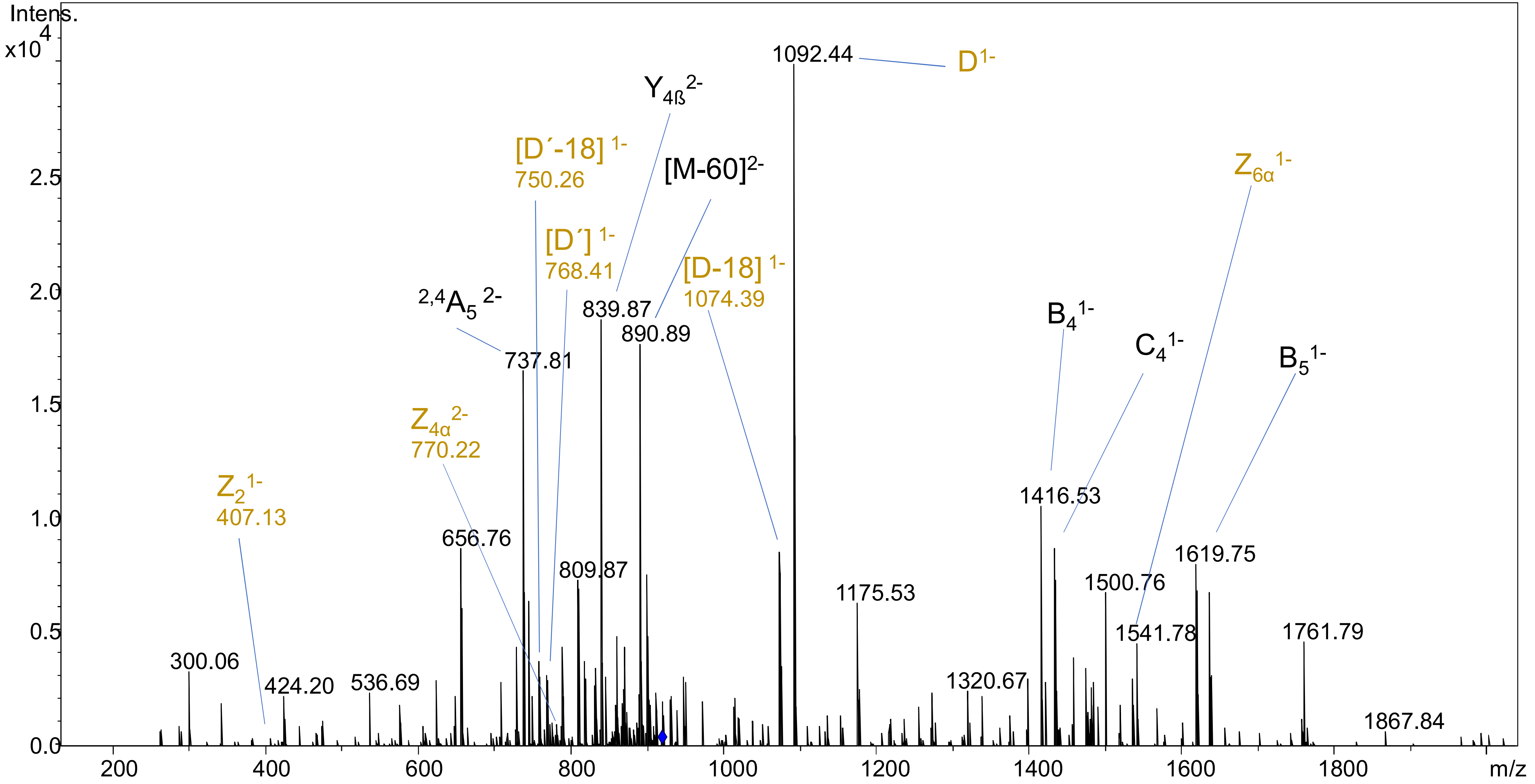

Glycan 16

M8P2
(H8N2P2)

**Monoisotopic mass:**	**1882.53 Da**
**Charge observed:**	**2-**
**Theoretical ion:**	***m/z* 940.26**
**Observed ion:**	***m/z* 940.37**
**Mass deviation:**	***m/z* 0.11**
**Retention time:**	**31.4 min**

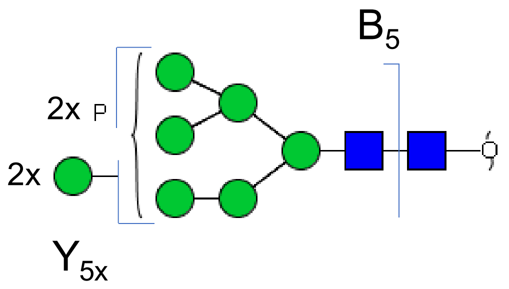

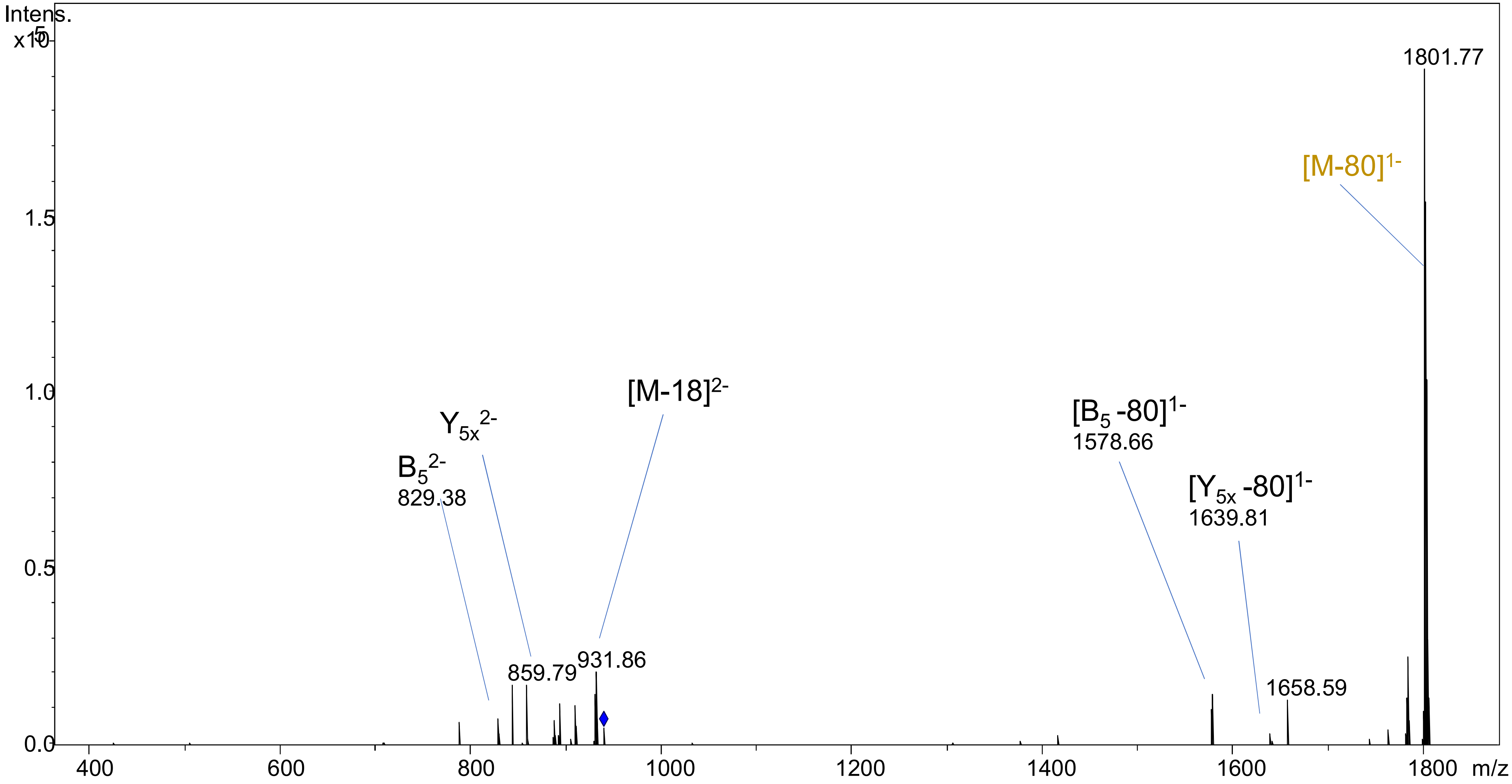

Glycan 17

A2G1F
(H4N4F1)

**Monoisotopic mass:**	**1626.61 Da**
**Charge observed:**	**2-**
**Theoretical ion:**	***m/z* 812.30**
**Observed ion:**	***m/z* 812.42**
**Mass deviation:**	***m/z* 0.12**
**Retention time:**	**60.2 min**

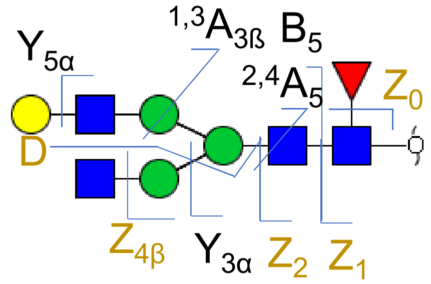

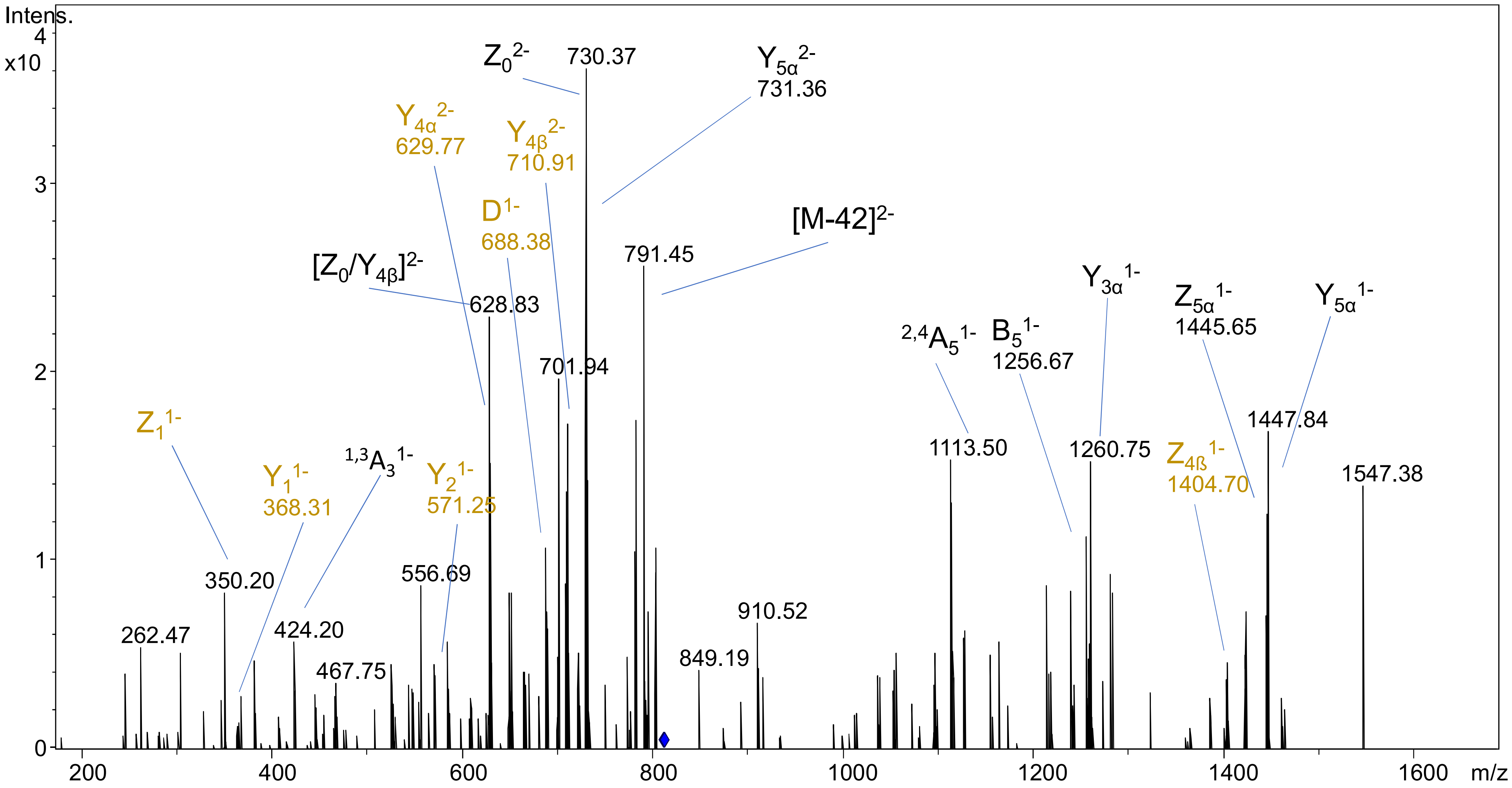

Glycan 18

A2G2
(H5N4)

Monoisotopic mass: **1642.61 Da**
Charge observed: **2-**
Theoretical ion: ***m/z* 820.30**
Observed ion: ***m/z* 820.44**
Mass deviation: ***m/z* 0.14**
Retention time: **55.9 min**

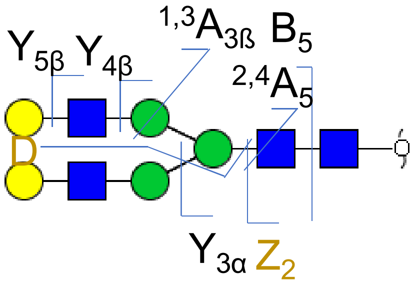

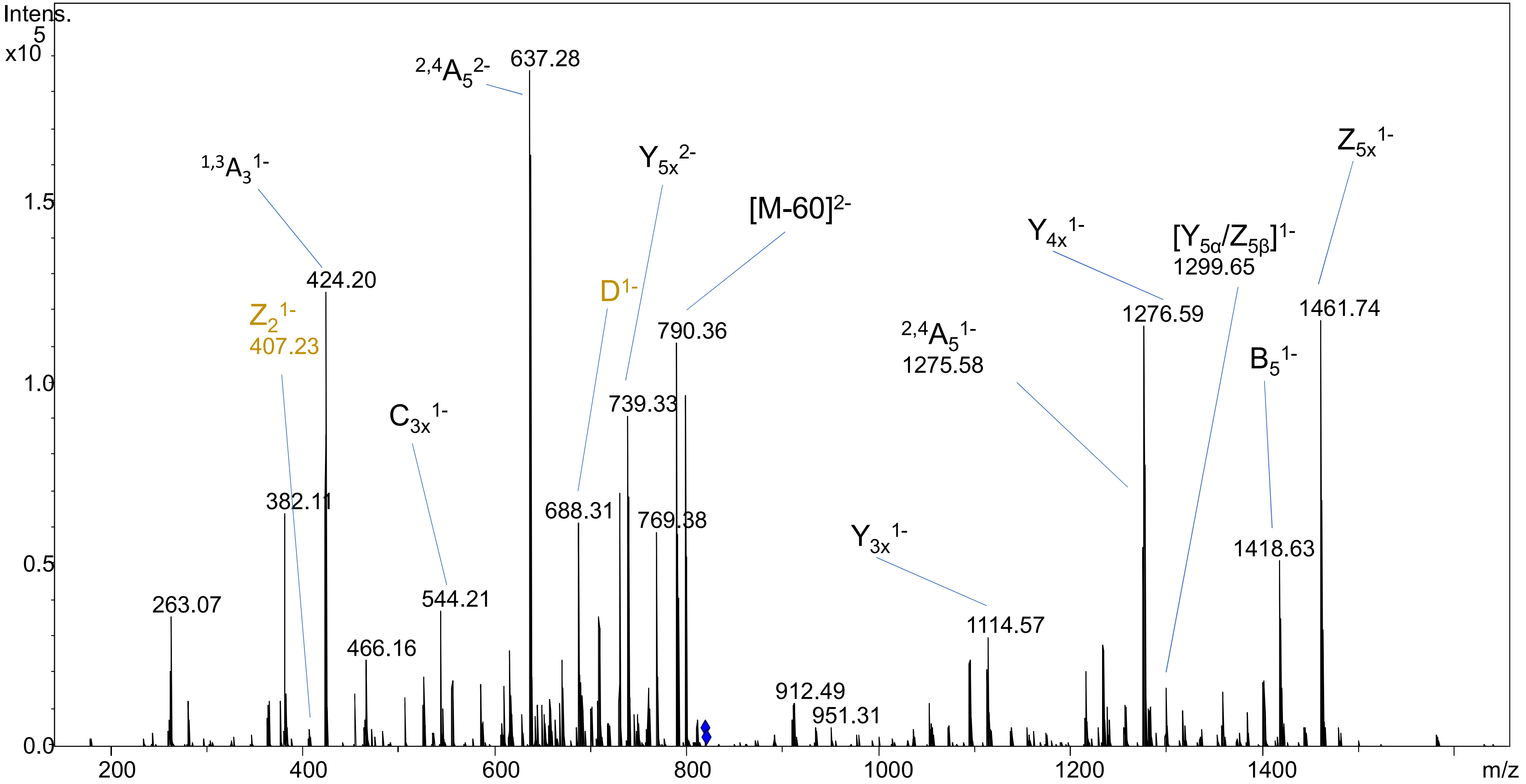

Glycan 19a

A2G0S1
(H4N4S1)

**Monoisotopic mass:**	**1771.64 Da**
**Charge observed:**	**1-**
**Theoretical ion:**	***m/z* 884.82**
**Observed ion:**	***m/z* 884.95**
**Mass deviation:**	***m/z* 0.13**
**Retention time:**	**60.5 min**

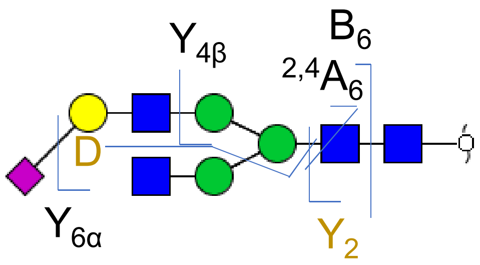

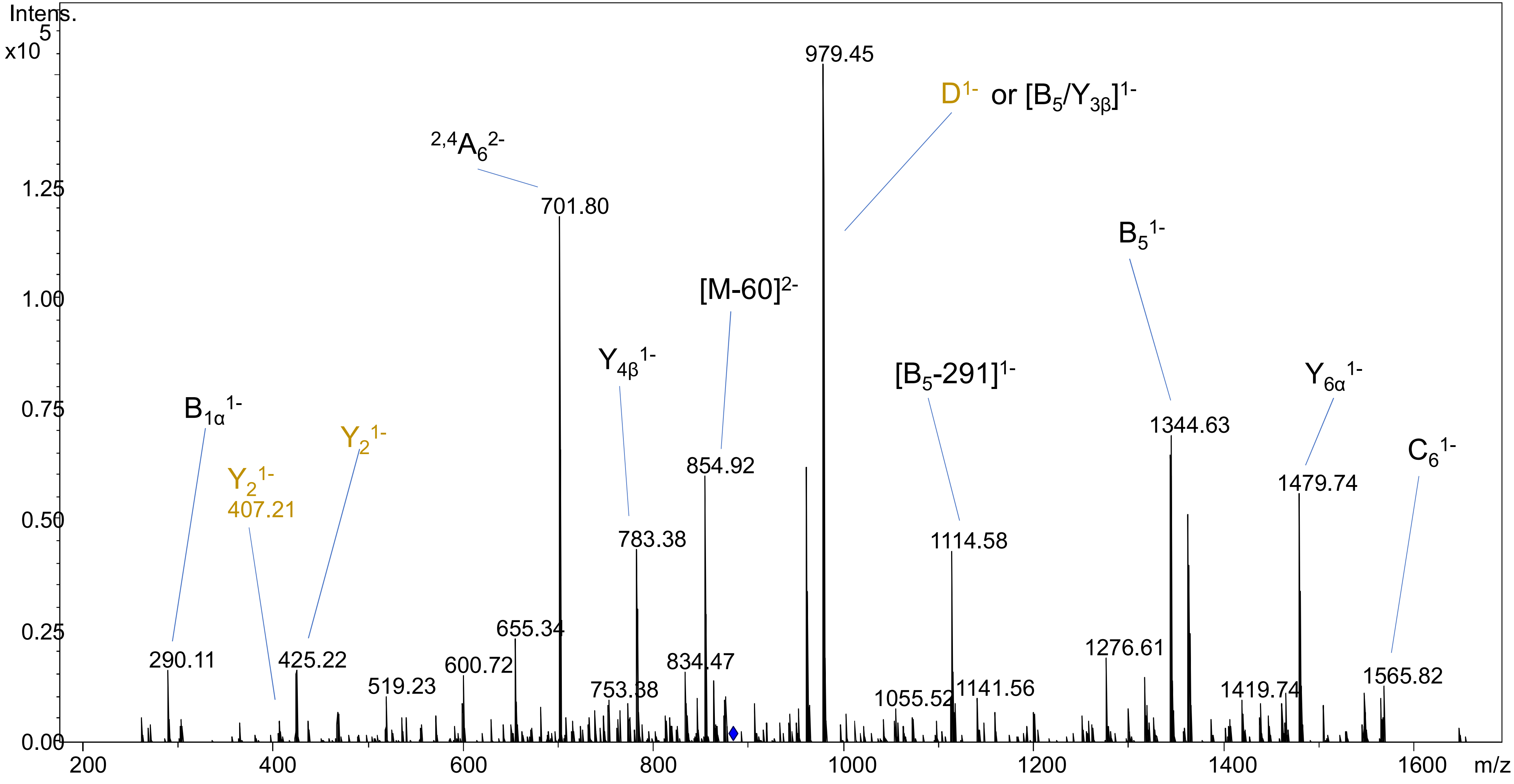

Glycan 19b

A1S1
(H4N4S1)

**Monoisotopic mass:**	**1771.64 Da**
**Charge observed:**	**1-**
**Theoretical ion:**	***m/z* 884.82**
**Observed ion:**	***m/z* 884.97**
**Mass deviation:**	***m/z* 0.13**
**Retention time:**	**61.6 min**

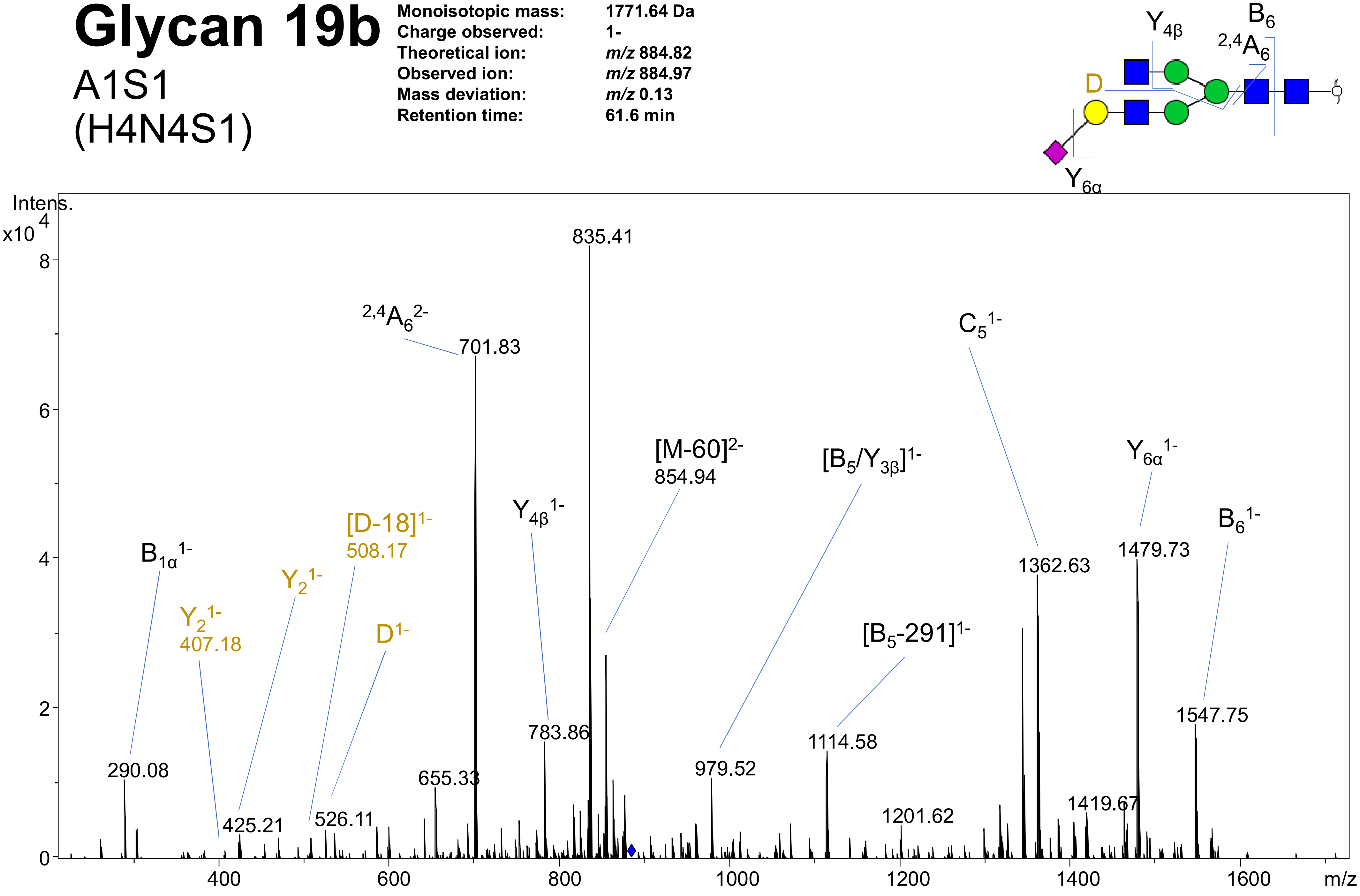

Glycan 20

A2G2F
(H5N4F1)

**Monoisotopic mass:**	**1788.67 Da**
**Charge observed:**	**2-**
**Theoretical ion:**	***m/z* 893.32**
**Observed ion:**	***m/z* 893.46**
**Mass deviation:**	***m/z* 0.14**
**Retention time:**	**63.3 min**

UniCarb-DB: #425

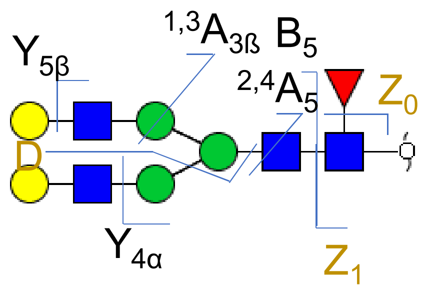

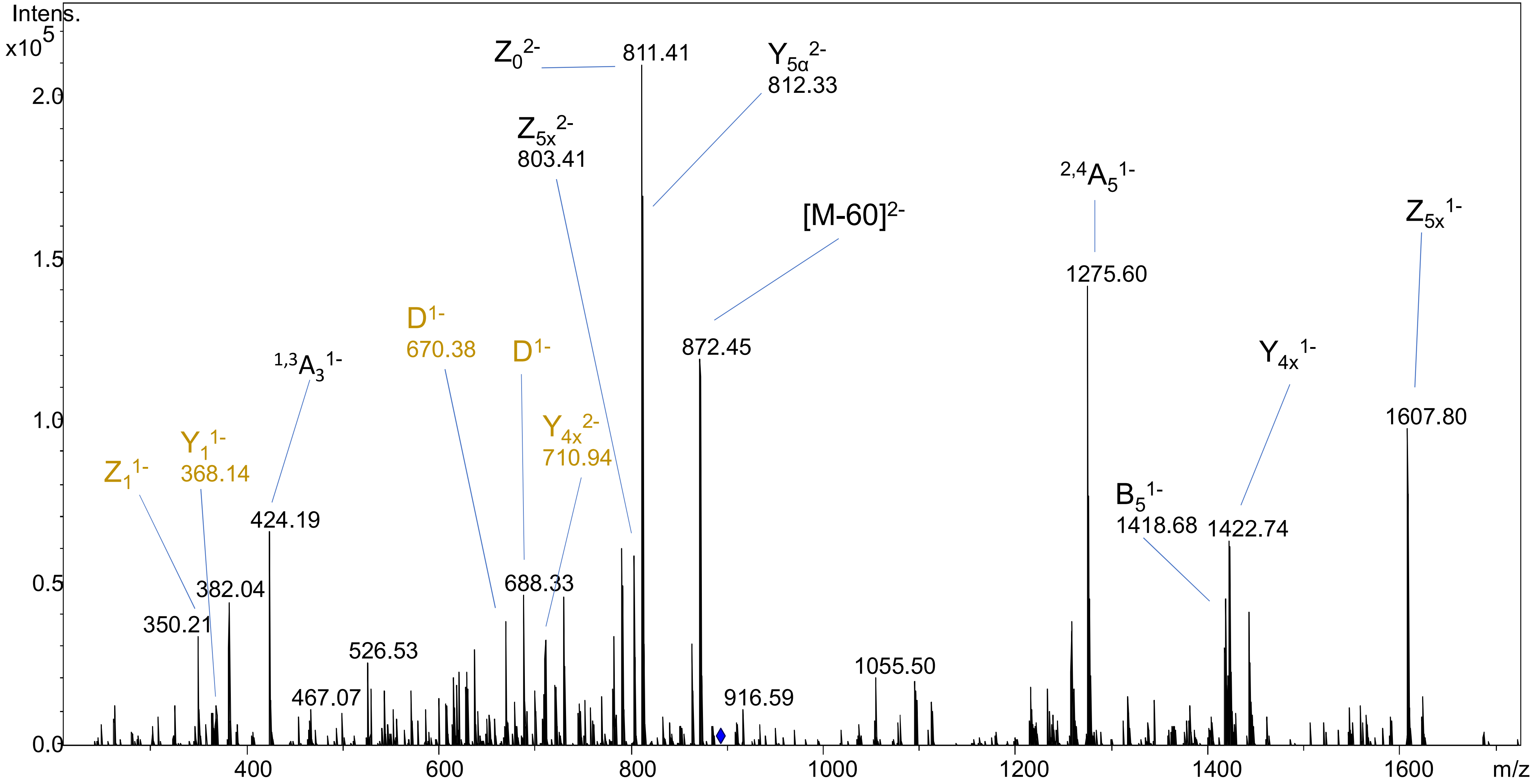

Glycan 21

A2G1S1
(H5N4S1)

Monoisotopic mass: 1933.70 Da
Charge observed: 2-
Theoretical ion: *m/z* 965.84
Observed ion: *m/z* 965.99
Mass deviation: *m/z* 0.15
Retention time: 63.8 min

UniCarb-DB: #2338

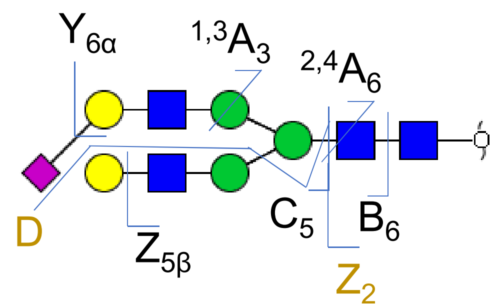

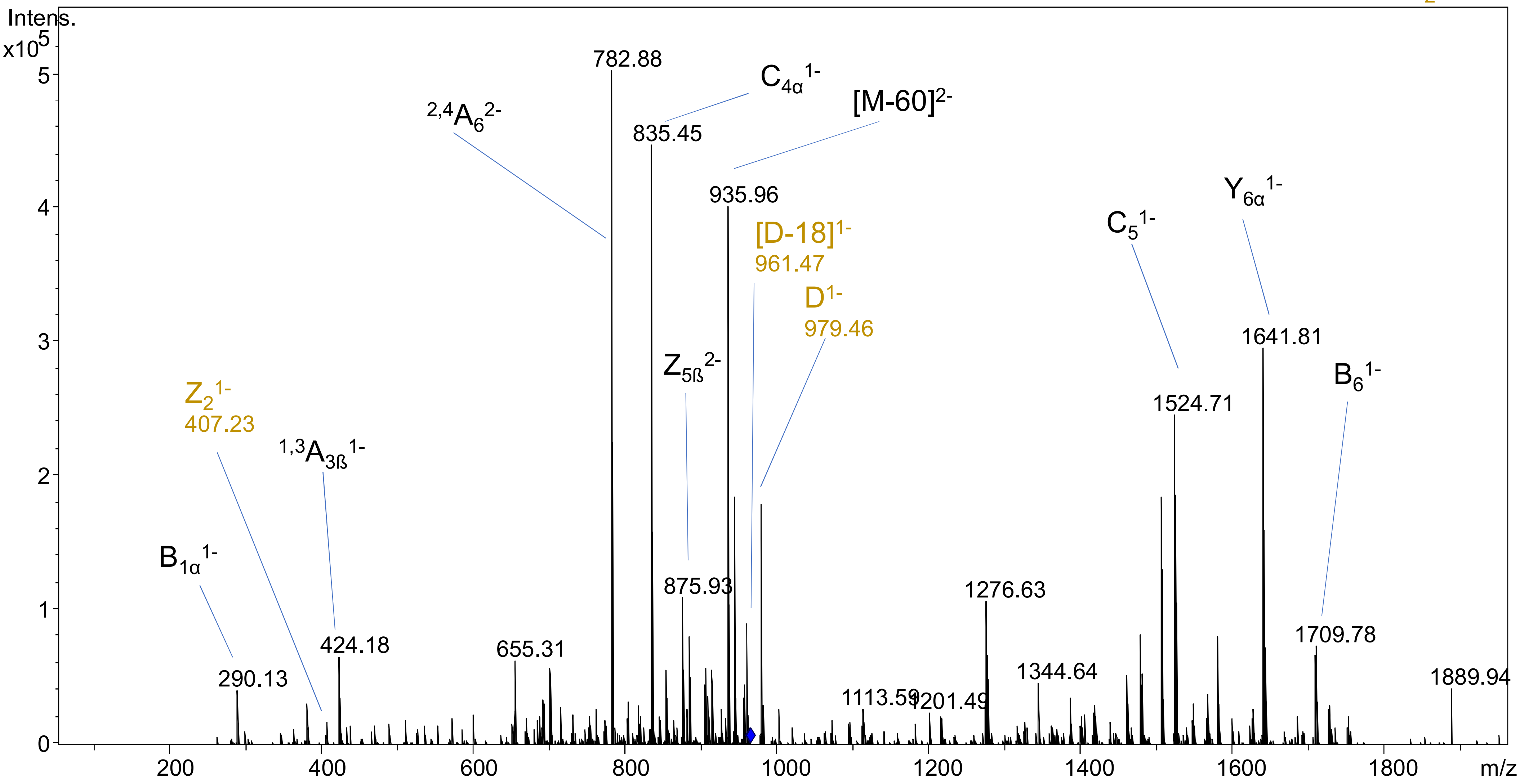

Glycan 22

A2G1S1Ac2
(H5N4S1Ac2)

Monoisotopic mass: **2017.72 Da**
Charge observed: **2-**
Theoretical ion: ***m/z* 1007.86**
Observed ion: ***m/z* 1007.92**
Mass deviation: ***m/z* 0.08**
Retention time: **63.9 min**
Note: No insightful MS2 available.

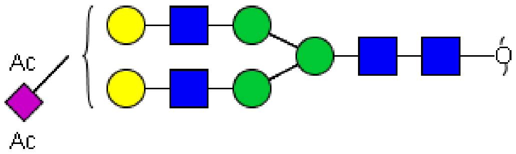

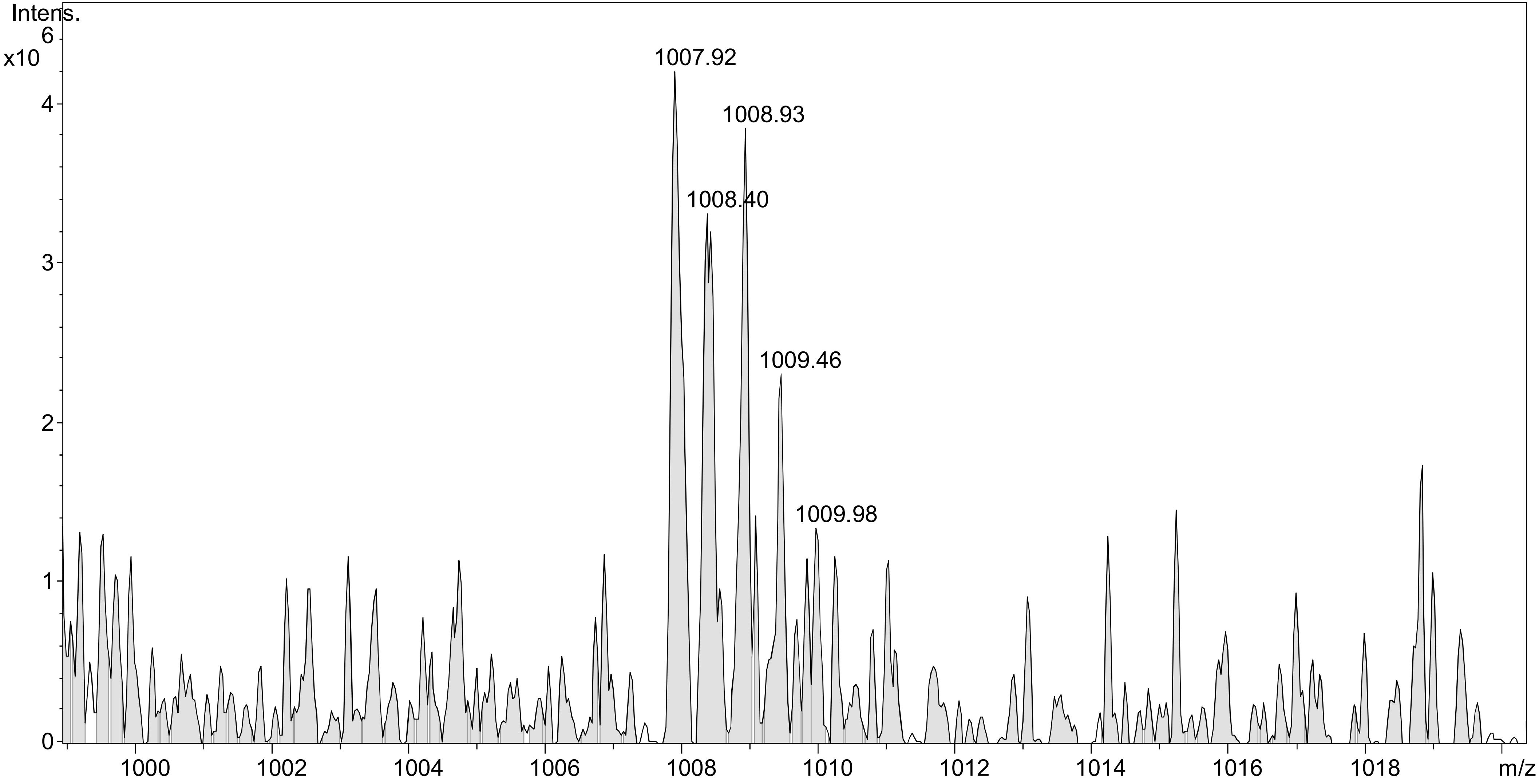

Glycan 23

A2G1S1F
(H5N4F1S1)

**Monoisotopic mass:**	**2079.76 Da**
**Charge observed:**	**2-**
**Theoretical ion:**	***m/z* 1038.87**
**Observed ion:**	***m/z* 1039.00**
**Mass deviation:**	***m/z* 0.13**
**Retention time:**	**69.6 min**

UniCarb-DB: #2364

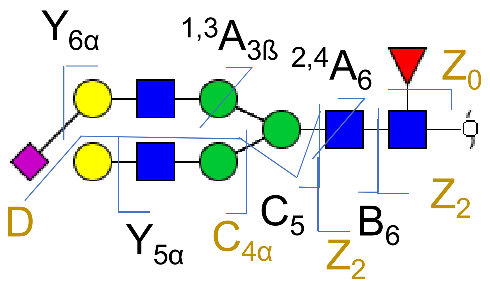

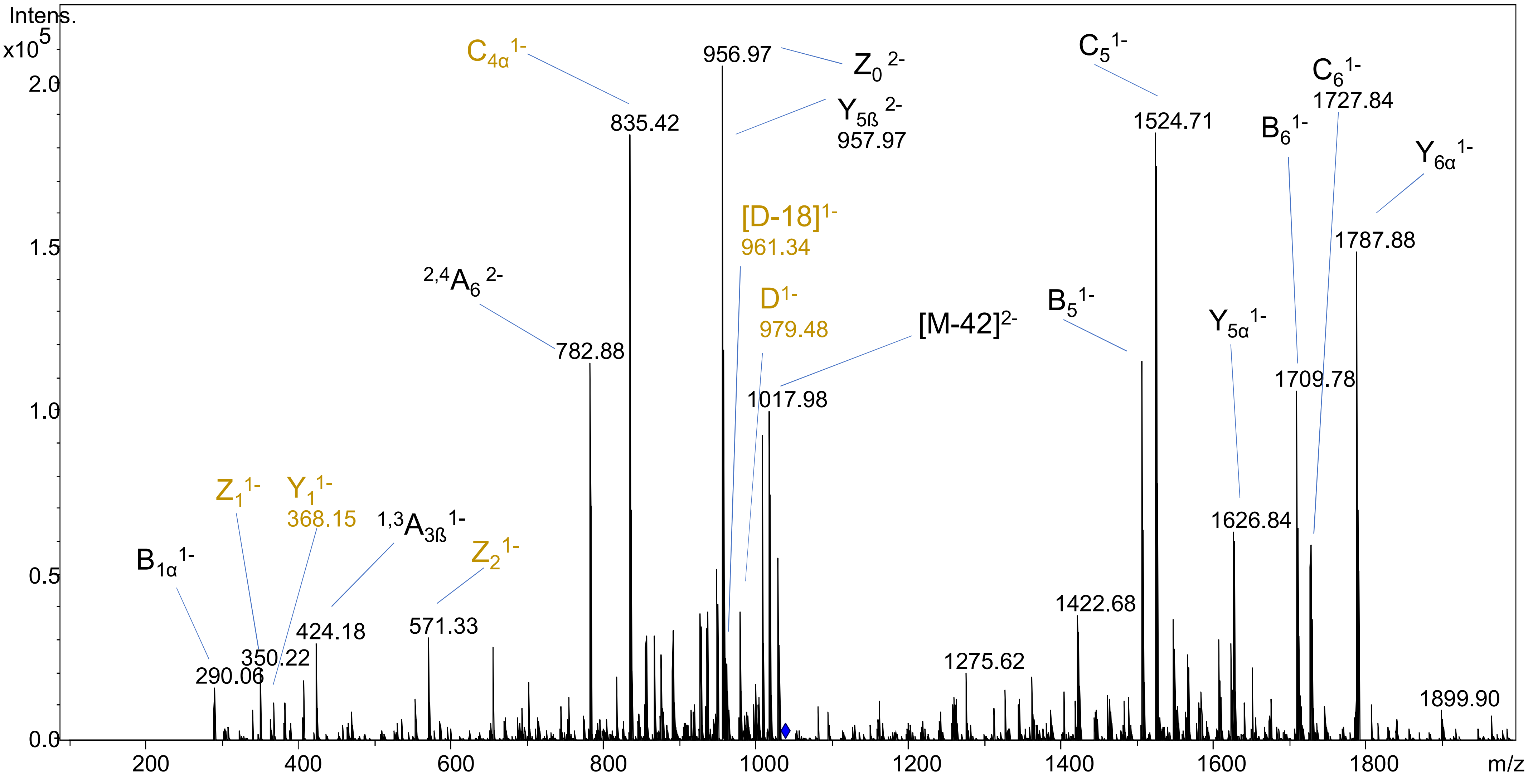

Glycan 24

A2G1S1FAc2
(H5N4F1S1Ac2)

Monoisotopic mass: 2163.78 Da
Charge observed: 2-
Theoretical ion: *m/z* 1080.88
Observed ion: *m/z* 1080.96
Mass deviation: *m/z* 0.08
Retention time: 69.3 min
Note: No insightful MS2 available.

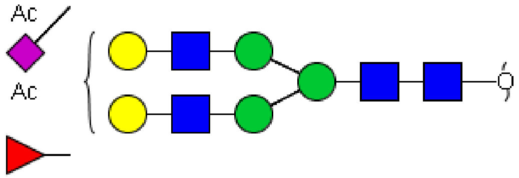

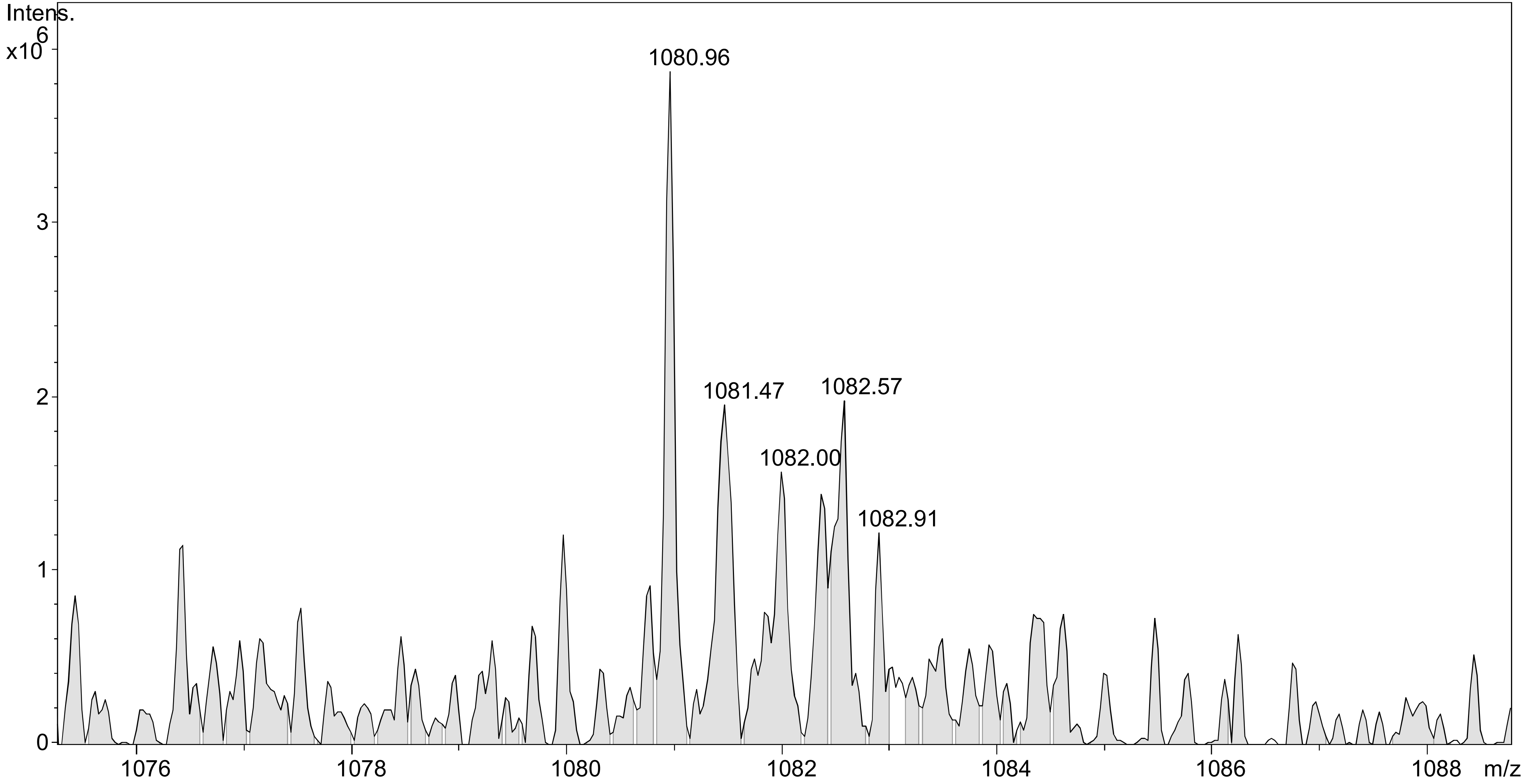

Glycan 25a

A2S2
(H5N4S2)

**Monoisotopic mass:**	**2224.80 Da**
**Charge observed:**	**2-**
**Theoretical ion:**	***m/z* 1111.42**
**Observed ion:**	***m/z* 1111.50**
**Mass deviation:**	***m/z* 0.08**
**Retention time:**	**62.1 min**

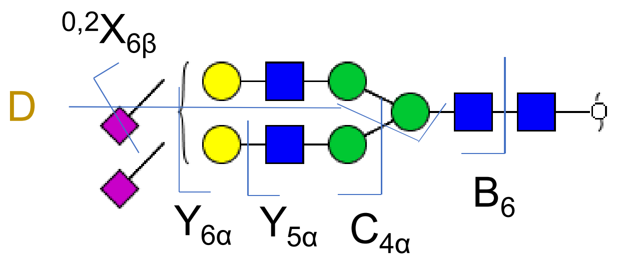

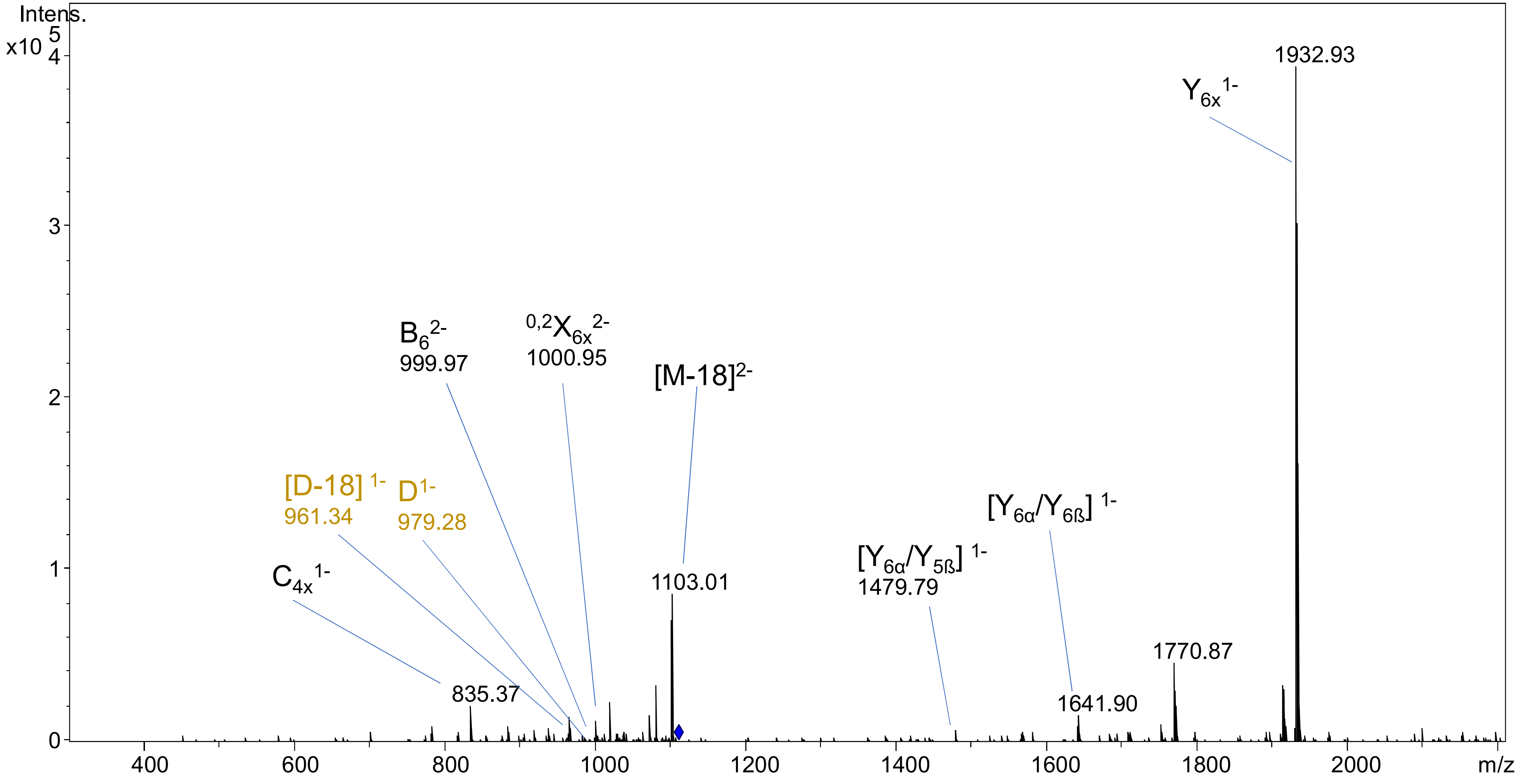

Glycan 25b

A2S2
(H5N4S2)

**Monoisotopic mass:**	**2224.80 Da**
**Charge observed:**	**2-**
**Theoretical ion:**	***m/z* 1111.42**
**Observed ion:**	***m/z* 1111.54**
**Mass deviation:**	***m/z* 0.12**
**Retention time:**	**70.0 min**

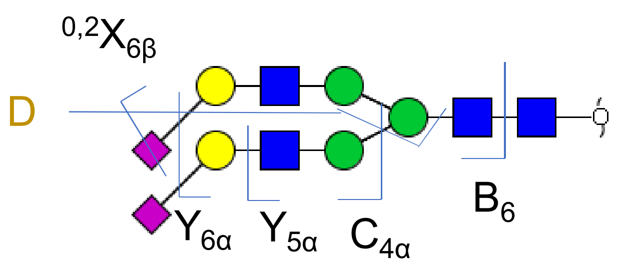

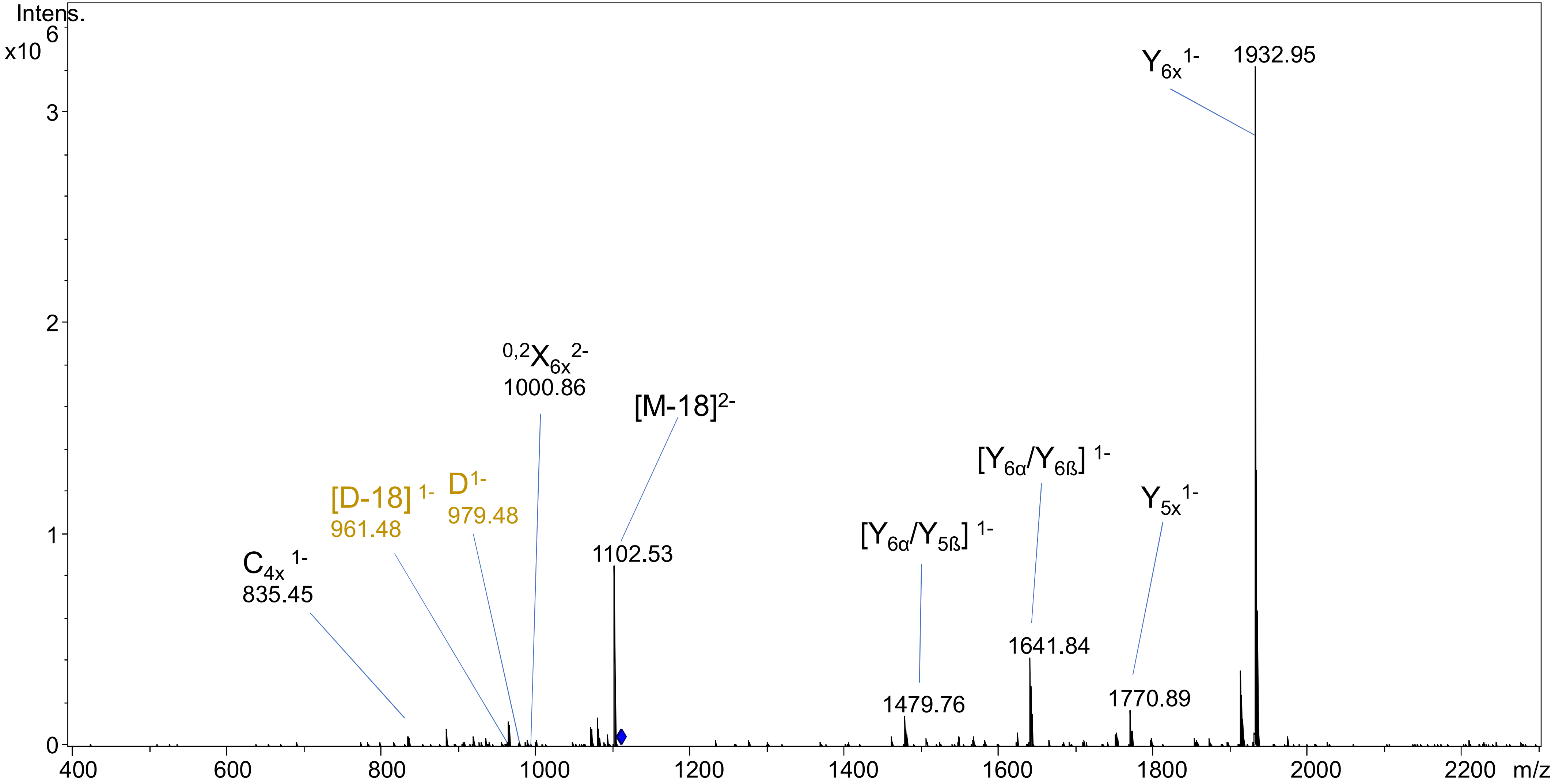

Glycan 26

A2S1Sg1
(H5N4S1G1)

Monoisotopic mass: 2240.79 Da
Charge observed: 2-
Theoretical ion: *m/z* 1119.39
Observed ion: *m/z* 1111.52
Mass deviation: *m/z* 0.13
Retention time: 69.3 min
Note: Isomers comprising the Neu5Ac either on the 3- or 6-arm potentially co-elute, which would explain the presence of both D-ions.

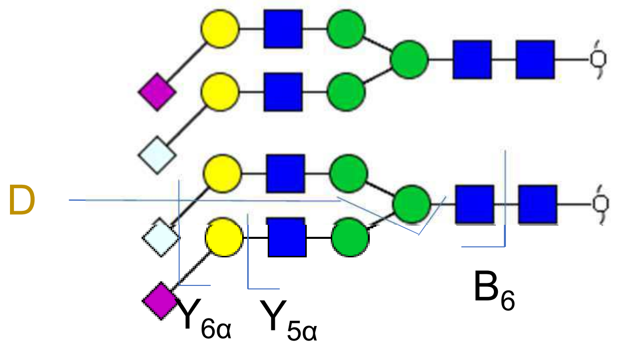

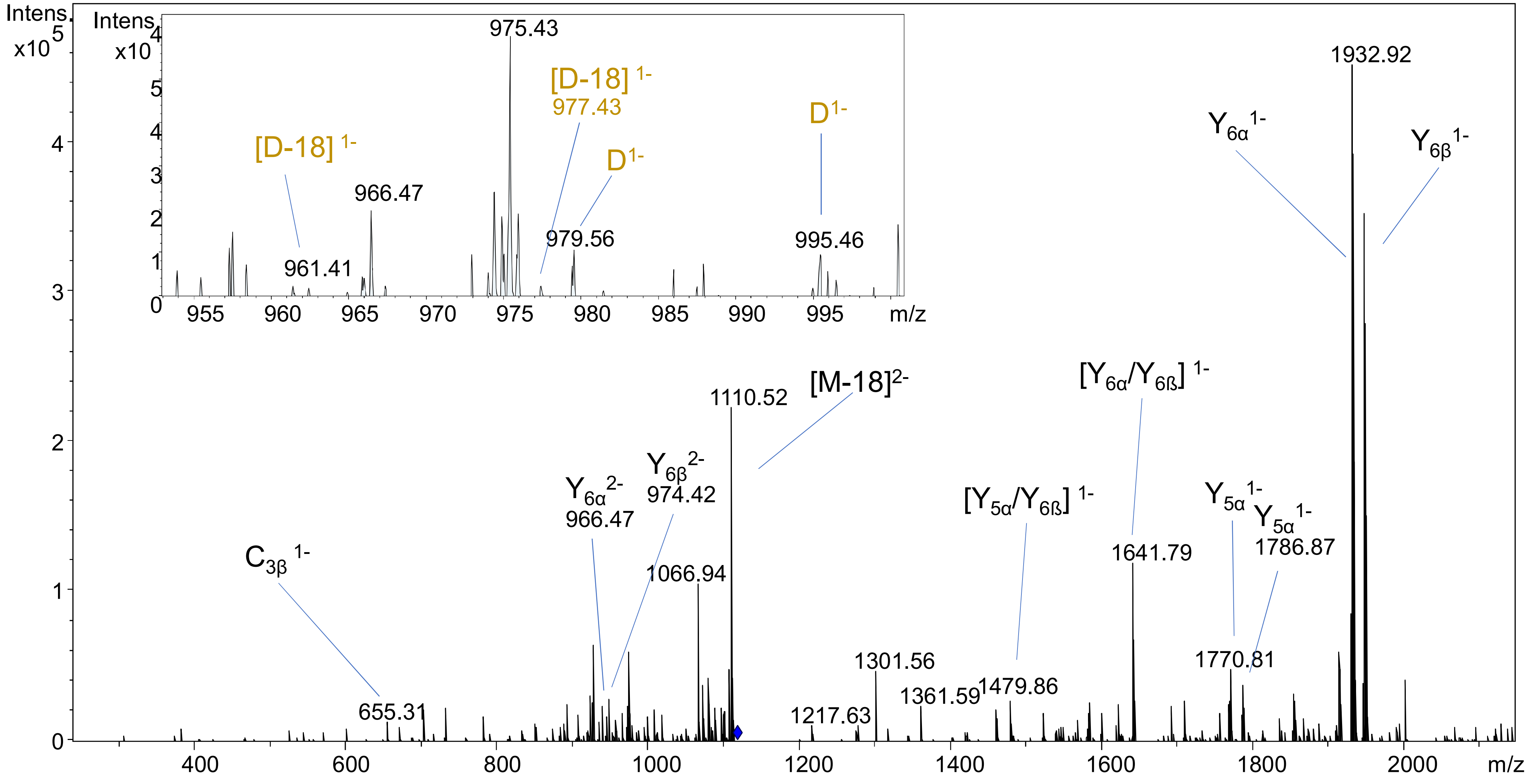

Glycan 27

A2S2Ac2
(H5N4S2Ac2)

**Monoisotopic mass:**	**2308.81 Da**
**Charge observed:**	**2-**
**Theoretical ion:**	***m/z* 1153.40**
**Observed ion:**	***m/z* 1153.47**
**Mass deviation:**	***m/z* 0.07**
**Retention time:**	**70.1 min**

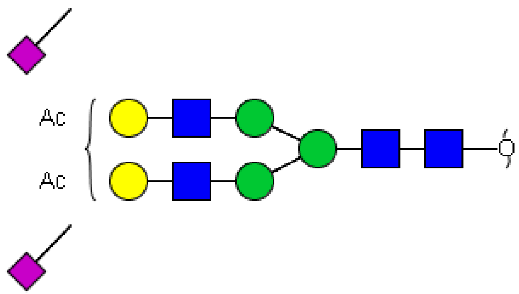

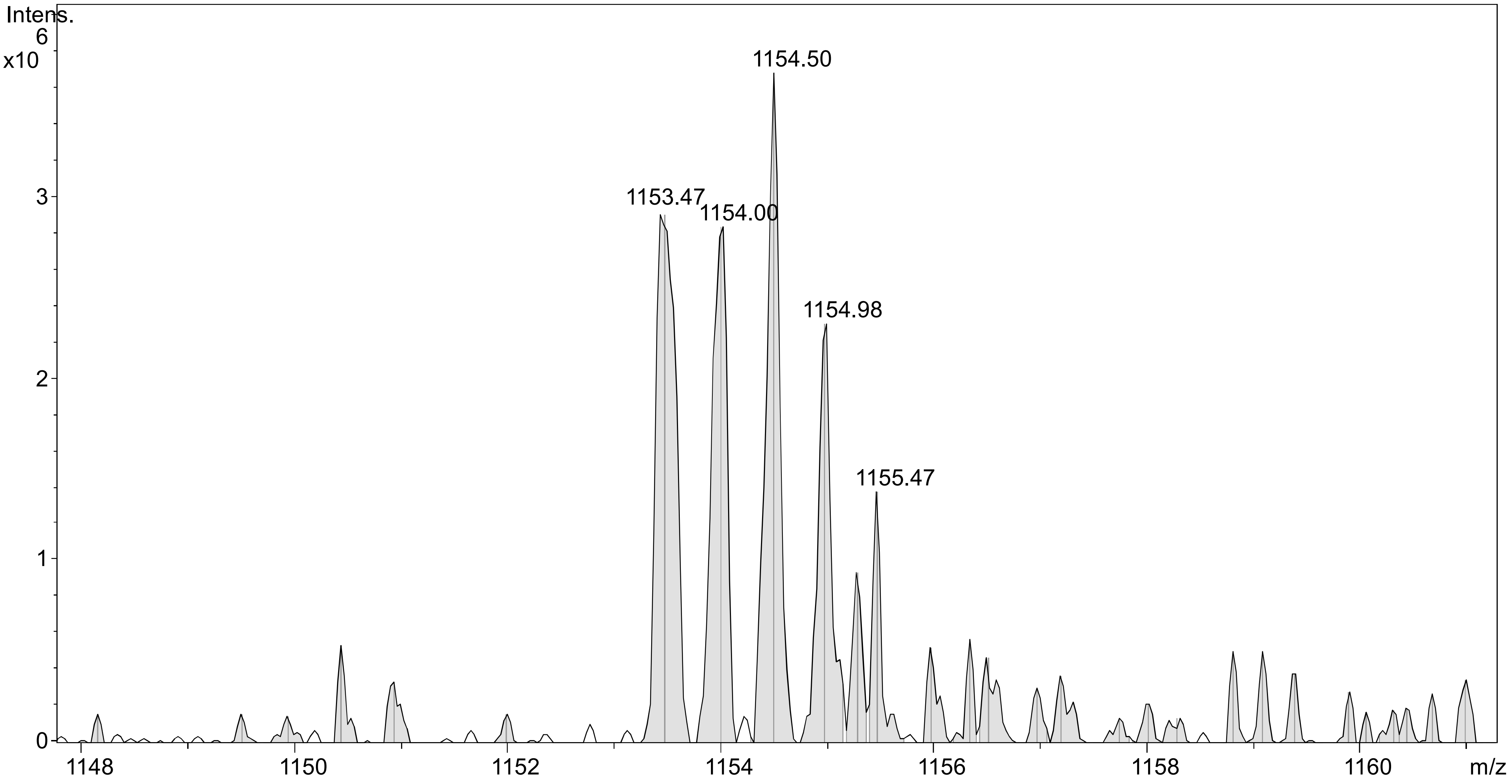

Glycan 28a

A2S2F
(H5N4S2F1)

Monoisotopic mass: **2370.86 Da**
Charge observed: **2-**
Theoretical ion: ***m/z* 1184.42**
Observed ion: ***m/z* 1184.57**
Mass deviation: ***m/z* 0.15**
Retention time: **67.5 min**

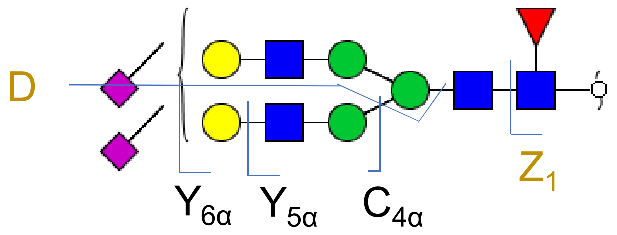

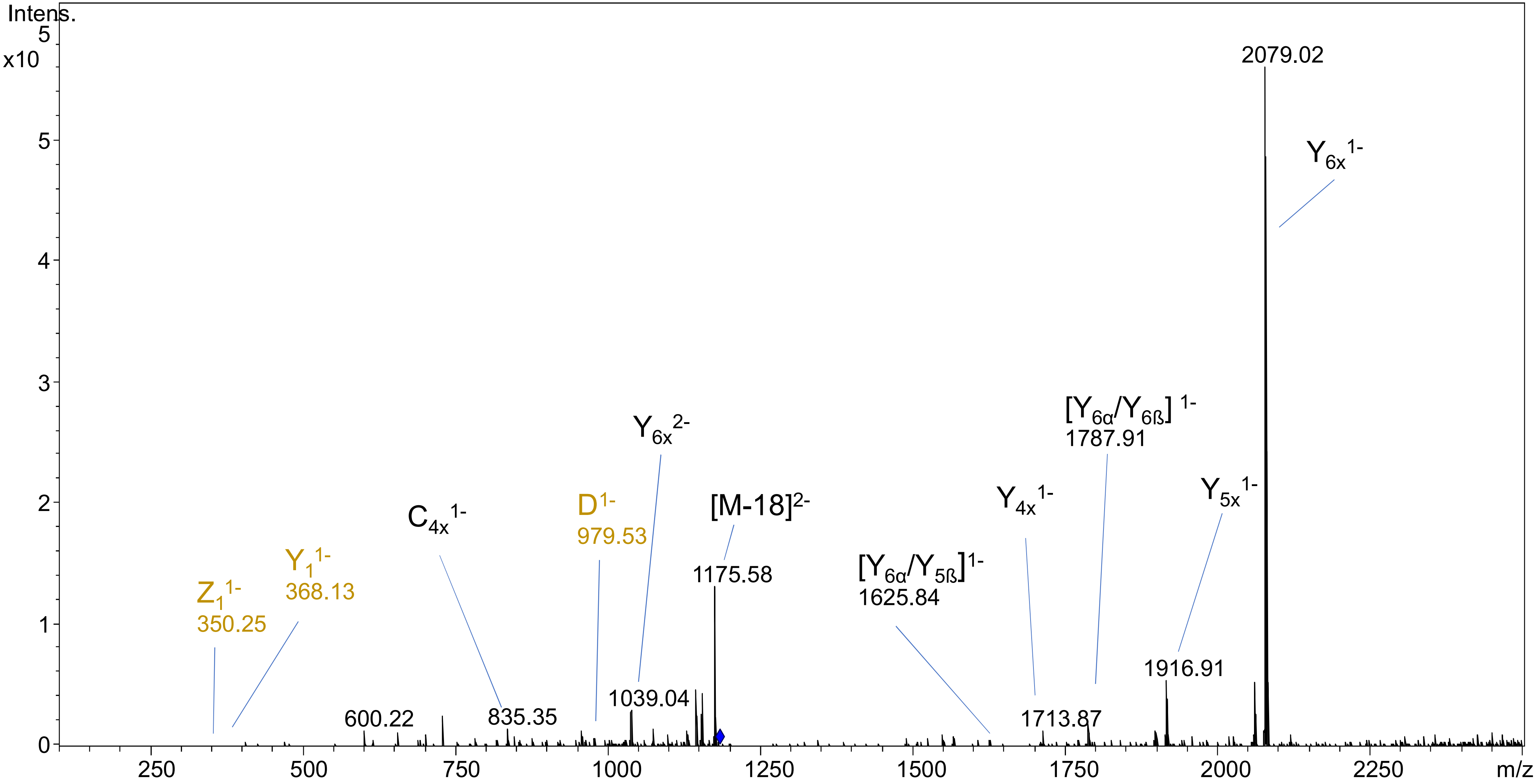

Glycan 28b

A2S2F
(H5N4S2F1)

Monoisotopic mass: **2370.86 Da**
Charge observed: **2-**
Theoretical ion: ***m/z* 1184.42**
Observed ion: ***m/z* 1184.60**
Mass deviation: ***m/z* 0.18**
Retention time: **74.9 min**

UniCarb-DB: #2563

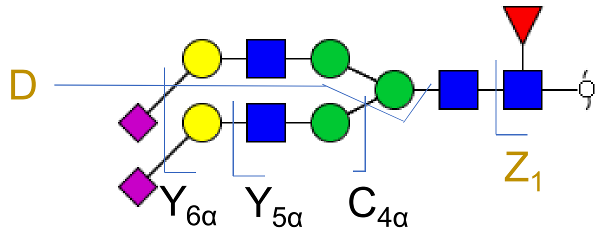

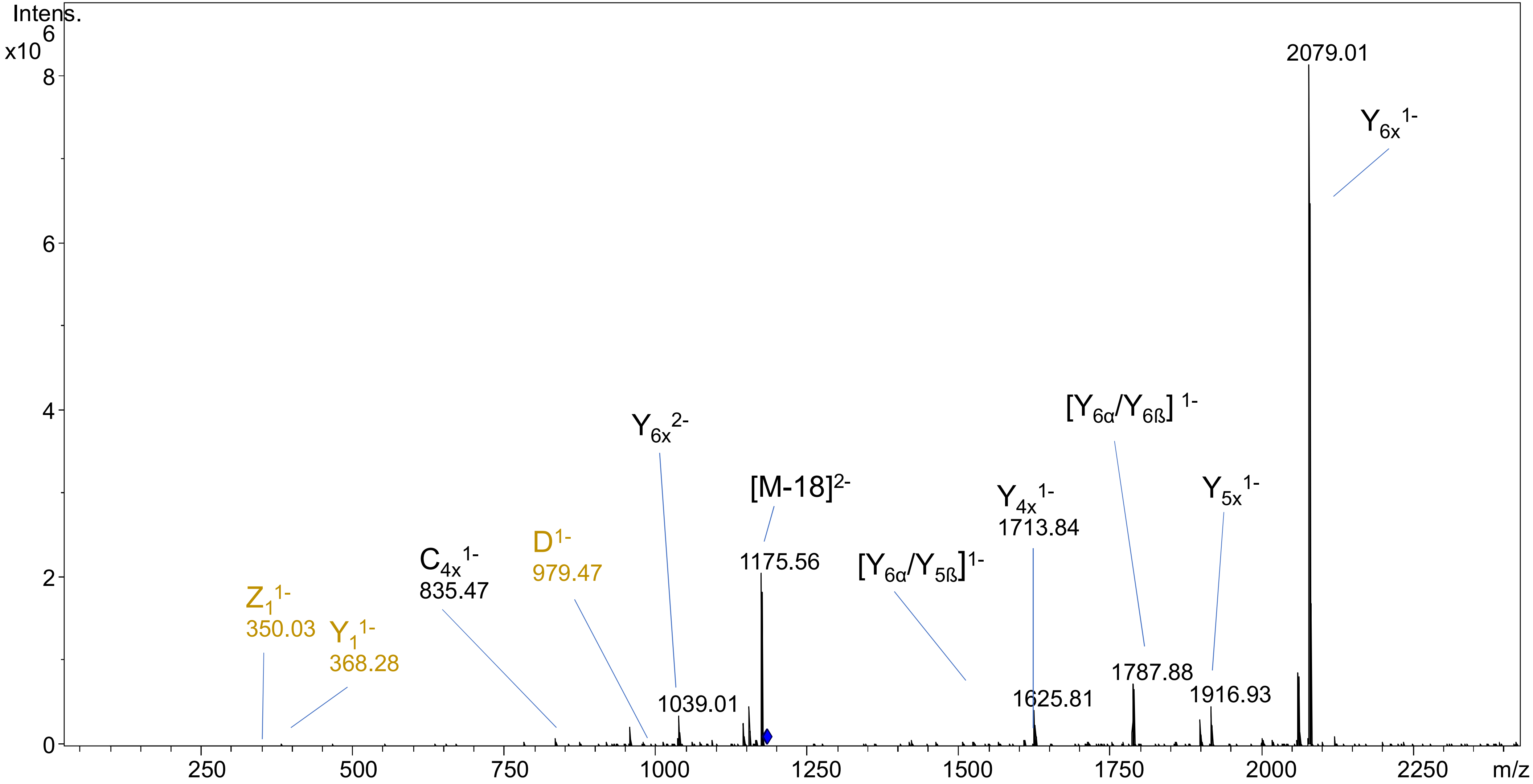

Glycan 29

A2S1Sg1F (H5N4F1S1G1)

Monoisotopic mass: 2385.85 Da
Charge observed: 2-
Theoretical ion: *m/z* 1192.42
Observed ion: *m/z* 1192.56
Mass deviation: *m/z* 0.14
Retention time: 74.4 min
Note: Isomers comprising the Neu5Ac either on the 3- or 6-arm potentially co-elute, which would explain the presence of both D-ions.

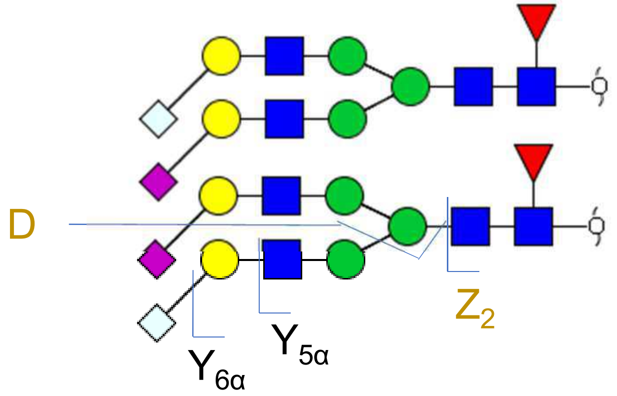

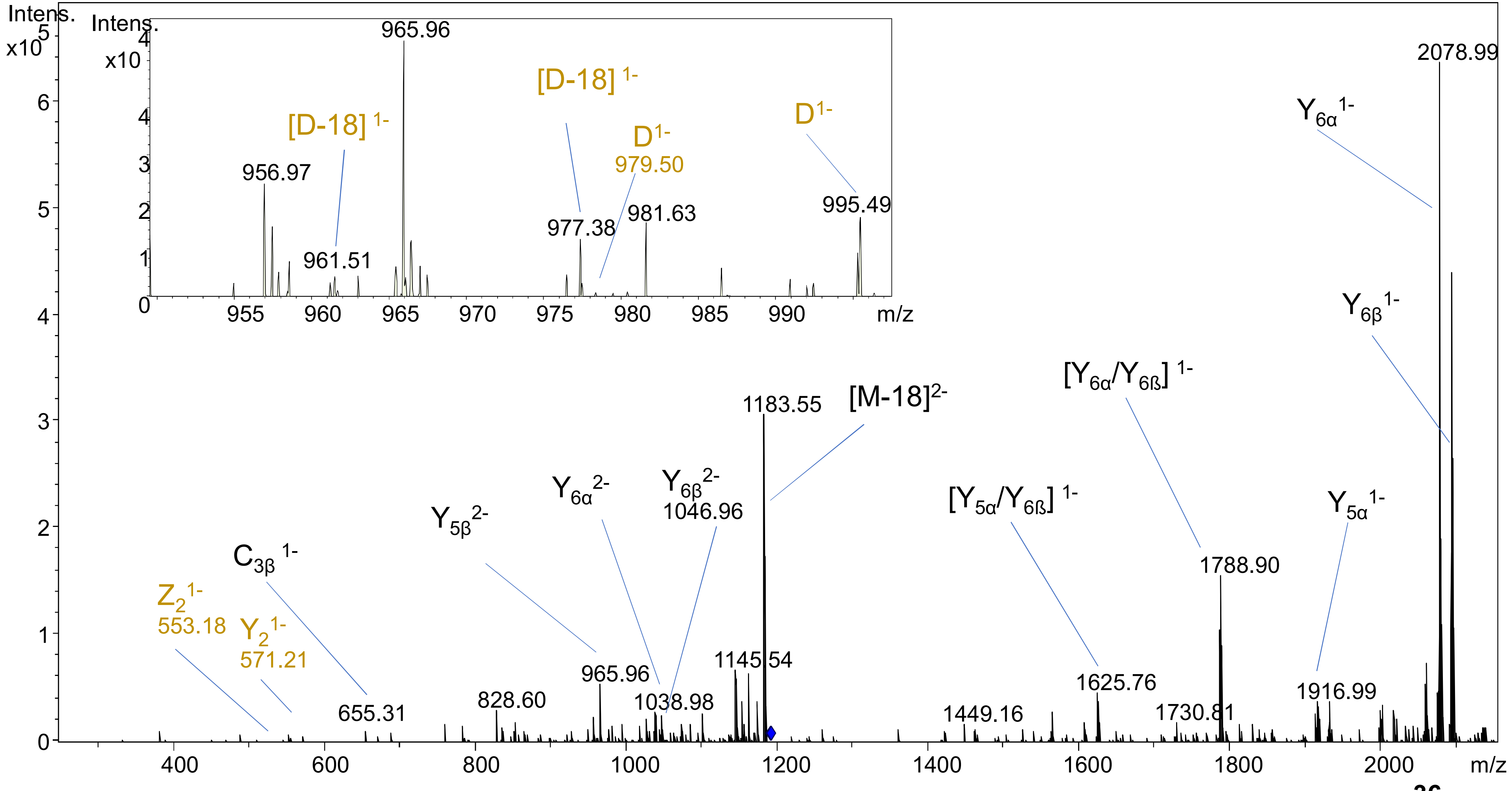

Glycan 30

A2S2Ac2F
(H5N4F1S2Ac2)

**Monoisotopic mass:**	**2454.87 Da**
**Charge observed:**	**2-**
**Theoretical ion:**	***m/z* 1226.43**
**Observed ion:**	***m/z* 1226.51**
**Mass deviation:**	***m/z* 0.08**
**Retention time:**	**75.5 min**

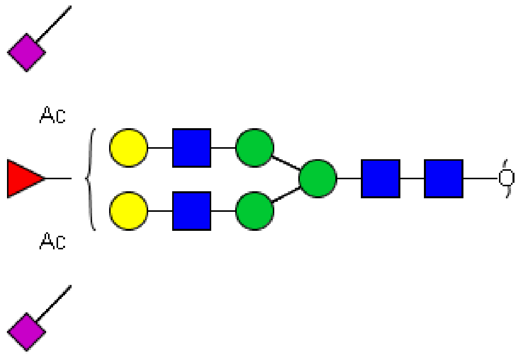

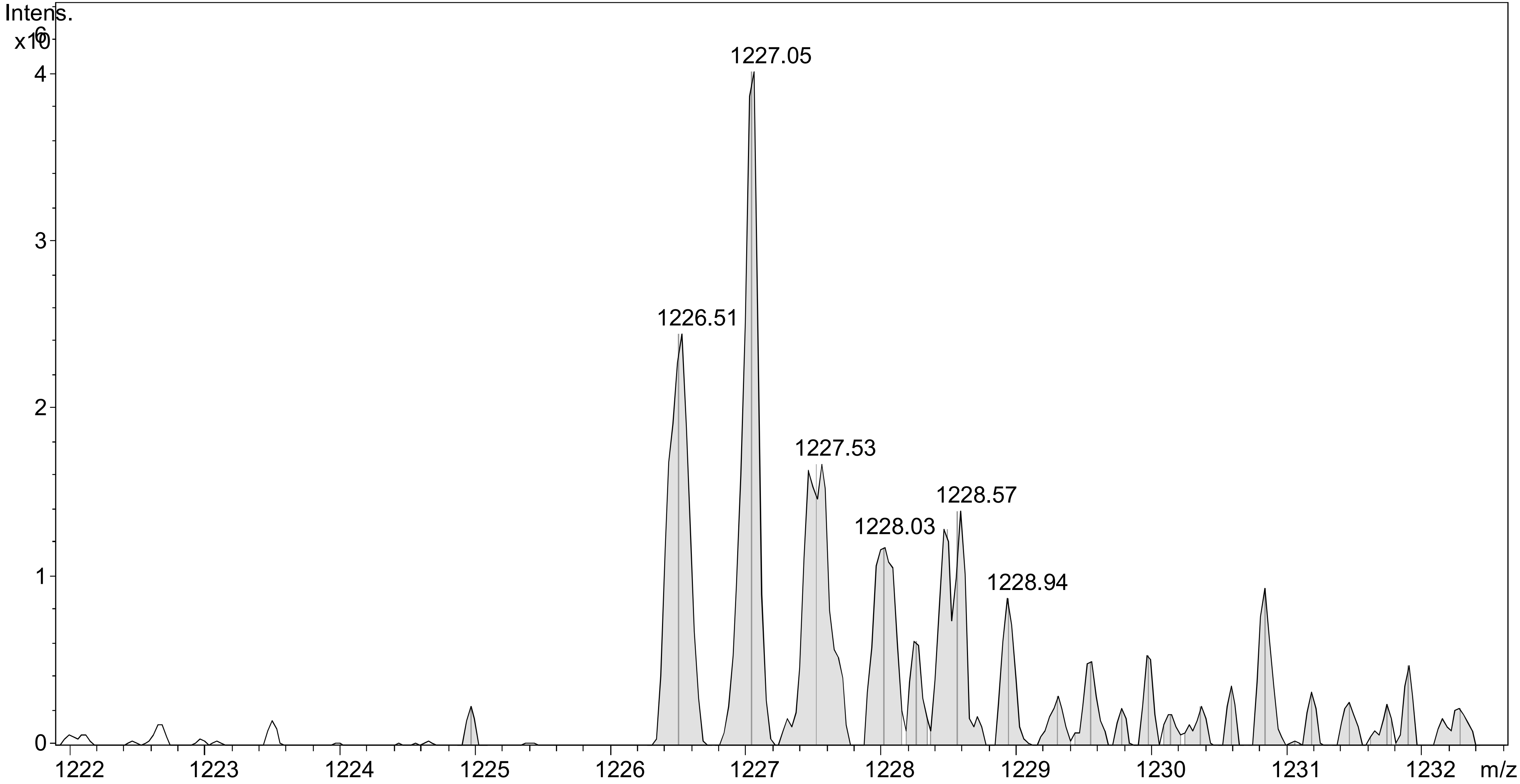

Glycan 31

A3G0S2F
(H5N5F1S2)

Monoisotopic mass: **2573.94 Da**
Charge observed: **2-**
Theoretical ion: ***m/z* 1285.96**
Observed ion: ***m/z* 1286.04**
Mass deviation: ***m/z* 0.09**
Retention time: **73.5 min**
Note: No MS2 was recorded.

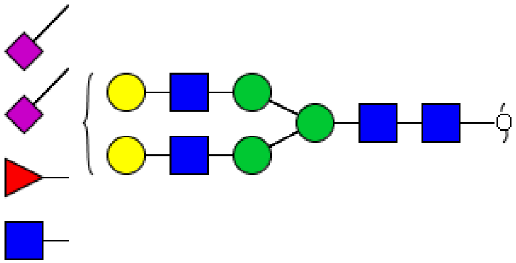

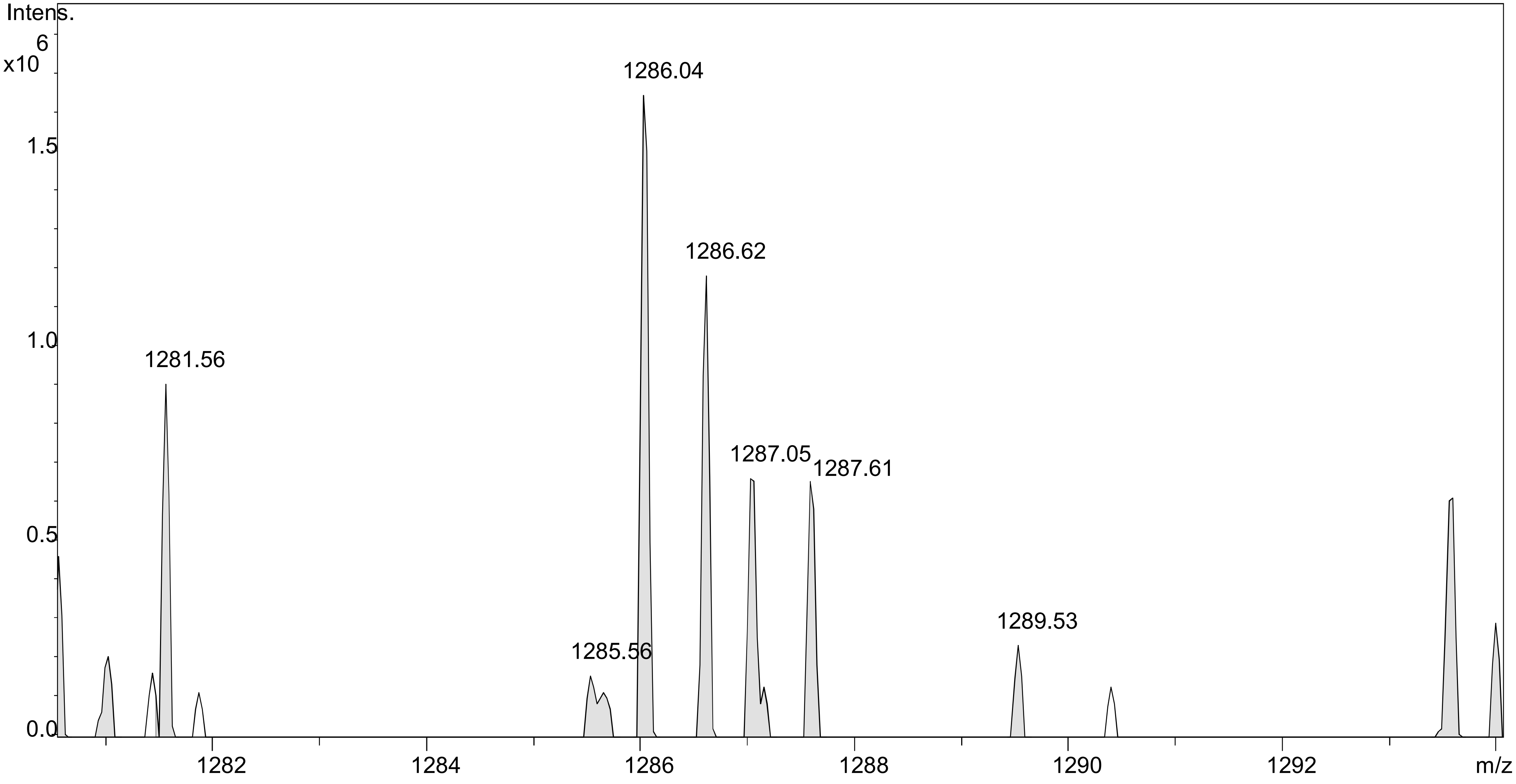

Glycan 32

A3G1S2F
(H6N5F1S2)

**Monoisotopic mass:**	**2735.98 Da**
**Charge observed:**	**2-**
**Theoretical ion:**	***m/z* 1366.99**
**Observed ion:**	***m/z* 1367.14**
**Mass deviation:**	***m/z* 0.15**
**Retention time:**	**71.8 min**

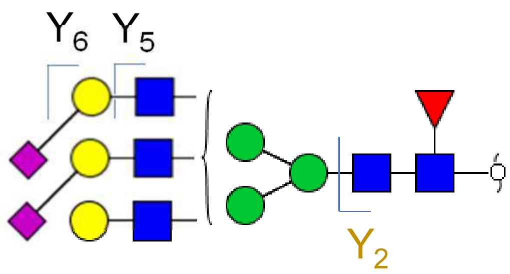

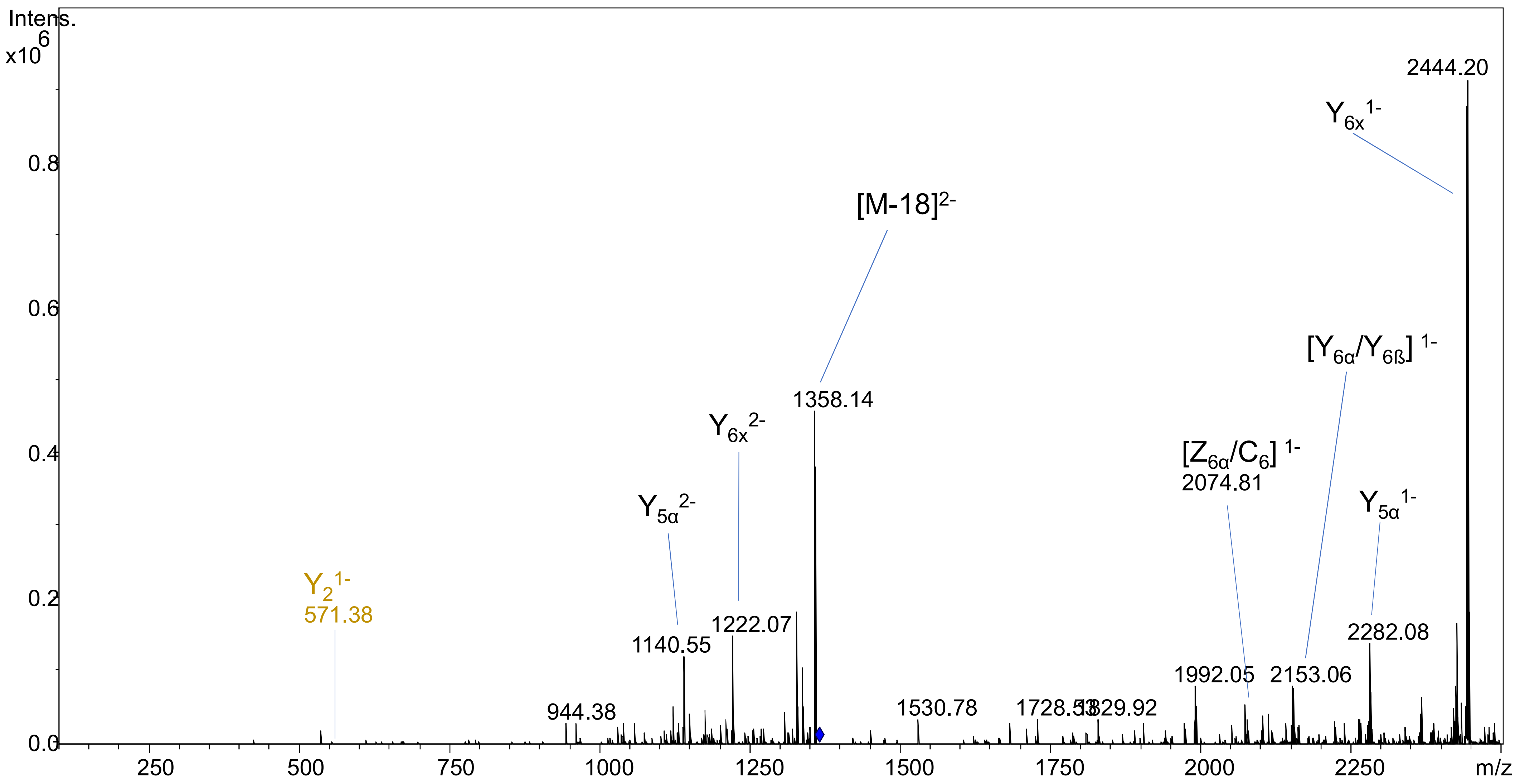

Glycan 33

A3S3
(H6N5S3)

**Monoisotopic mass:**	**2881.03 Da**
**Charge observed:**	**2-**
**Theoretical ion:**	***m/z* 1439.51**
**Observed ion:**	***m/z* 1439.68**
**Mass deviation:**	***m/z* 0.17**
**Retention time:**	**73.8 min**

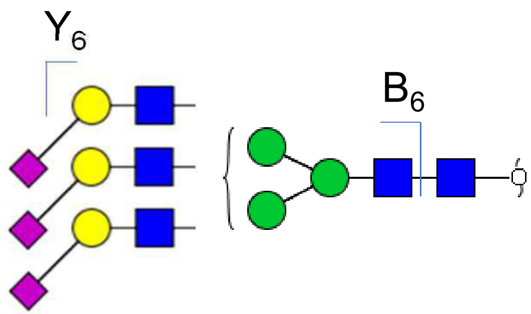

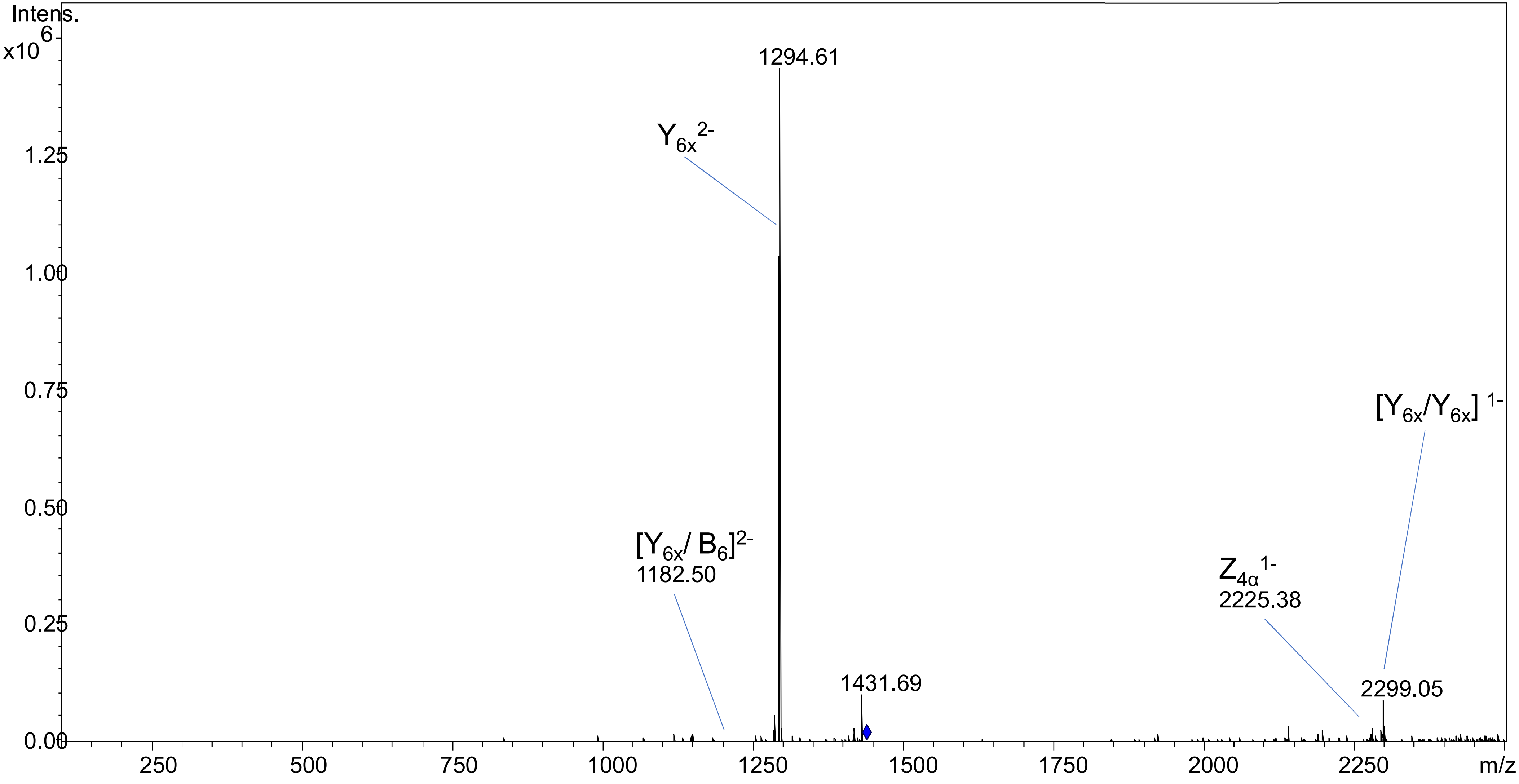

Glycan 34a

A3S3F
(H6N5F1S3)

**Monoisotopic mass:**	**3027.08 Da**
**Charge observed:**	**2-**
**Theoretical ion:**	***m/z* 1512.54**
**Observed ion:**	***m/z* 1512.69**
**Mass deviation:**	***m/z* 0.15**
**Retention time:**	**76.6 min**

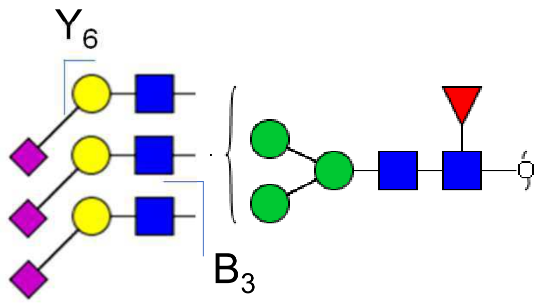

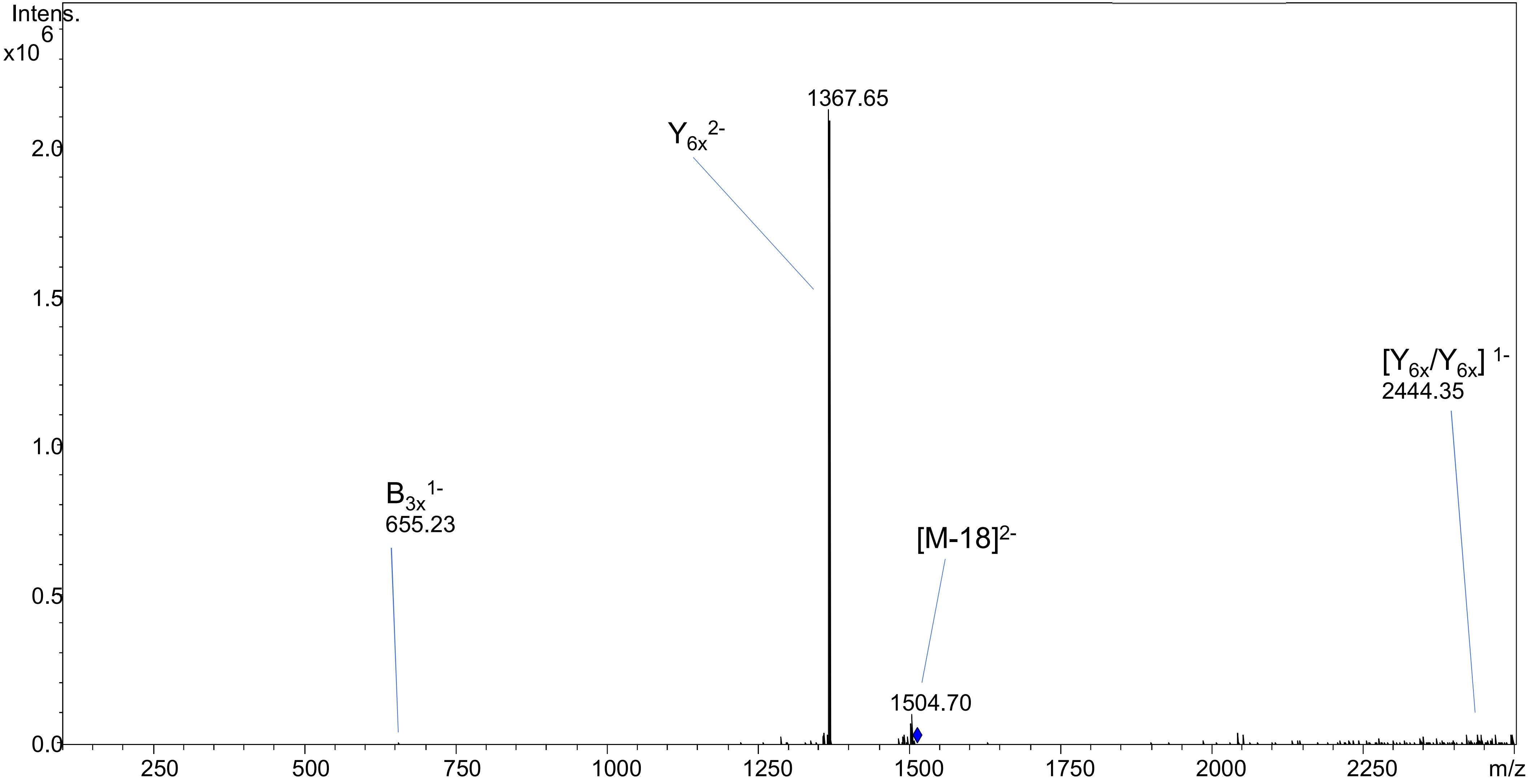

Glycan 34b

A3S3F
(H6N5F1S3)

**Monoisotopic mass:**	**3027.08 Da**
**Charge observed:**	**2-**
**Theoretical ion:**	***m/z* 1512.54**
**Observed ion:**	***m/z* 1512.68**
**Mass deviation:**	***m/z* 0.14**
**Retention time:**	**78.4 min**

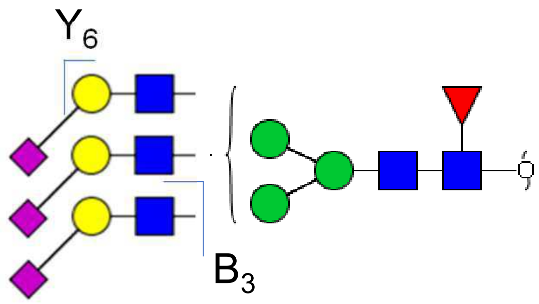

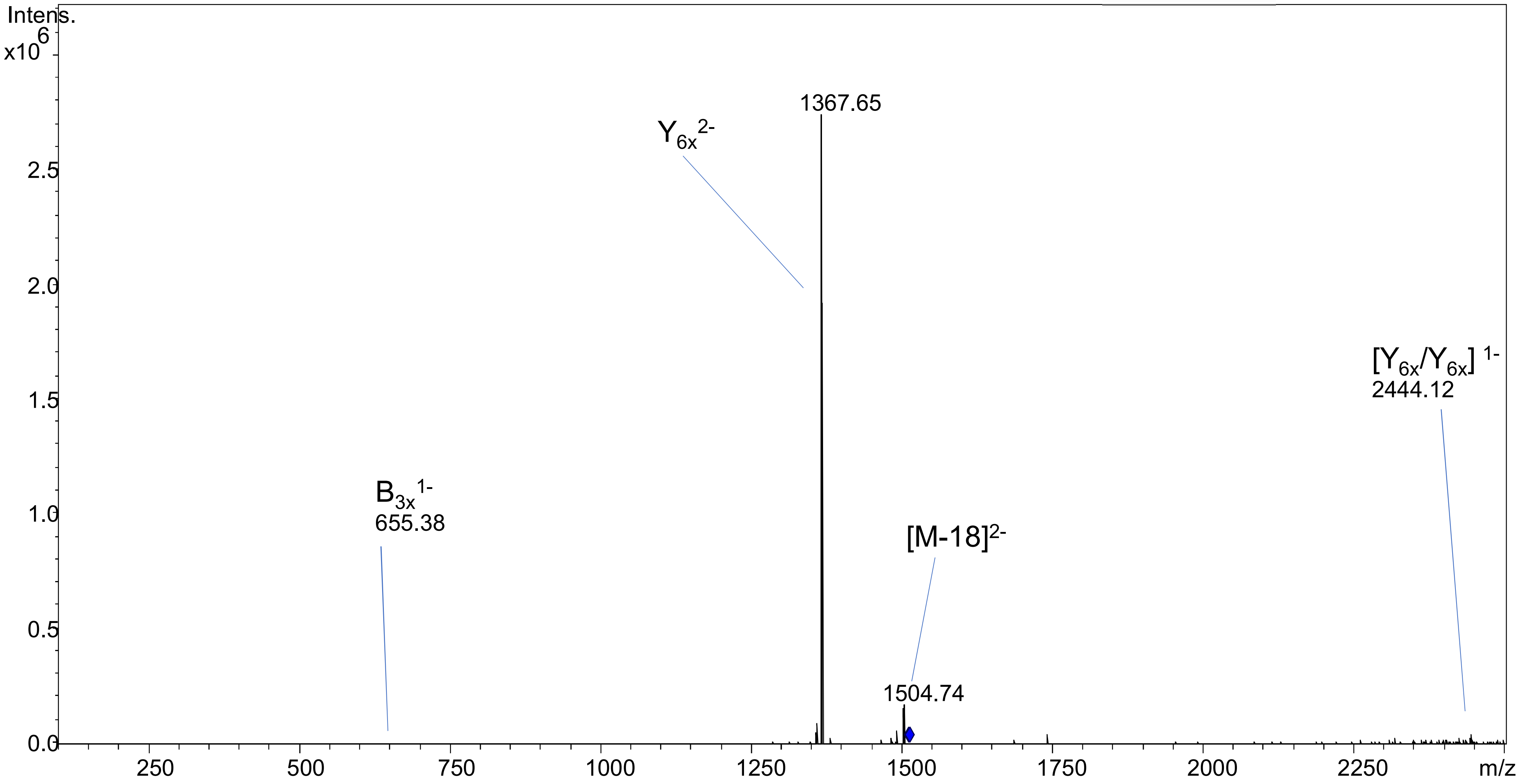

Glycan 35

A4S4F
(H7N6F1S4)

**Monoisotopic mass:**	**3683.31 Da**
**Charge observed:**	**3-**
**Theoretical ion:**	***m/z* 1226.76**
**Observed ion:**	***m/z* 1226.87**
**Mass deviation:**	***m/z* 0.11**
**Retention time:**	**67.8 min**

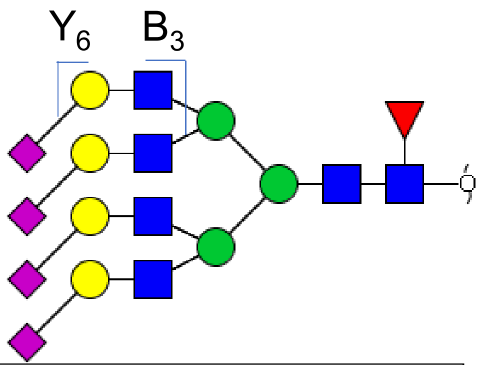

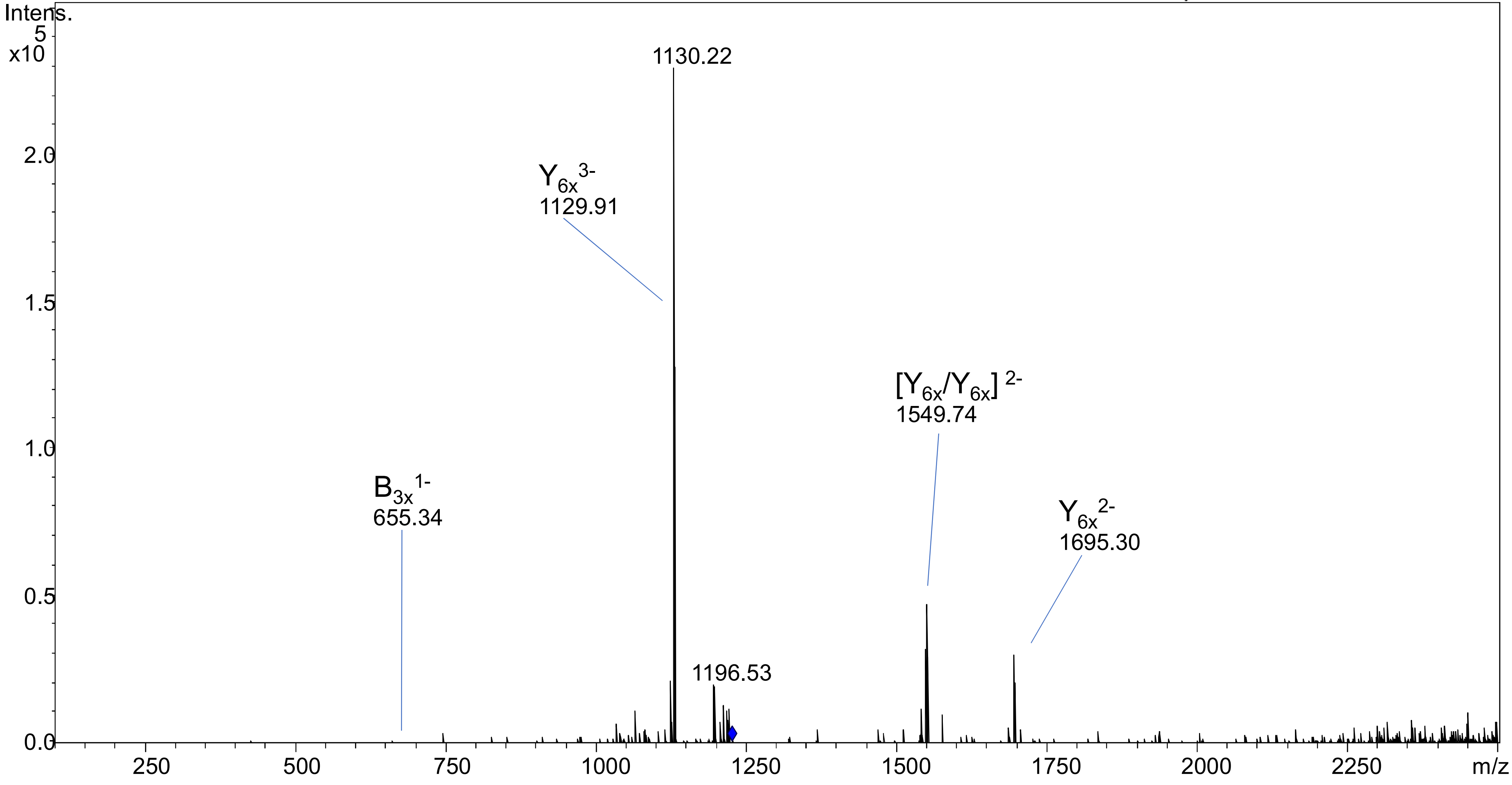

Glycan 36

A1G0
(H3N3)

**Monoisotopic mass:**	**1115.42 Da**
**Charge observed:**	**1-**
**Theoretical ion:**	***m/z* 1114.42**
**Observed ion:**	***m/z* 1114.55**
**Mass deviation:**	***m/z* 0.13**
**Retention time:**	**59.4 min**

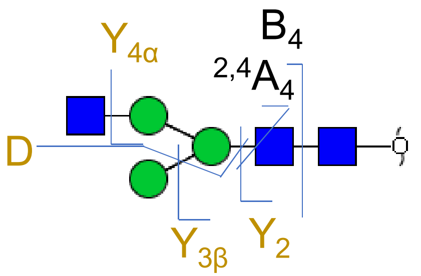

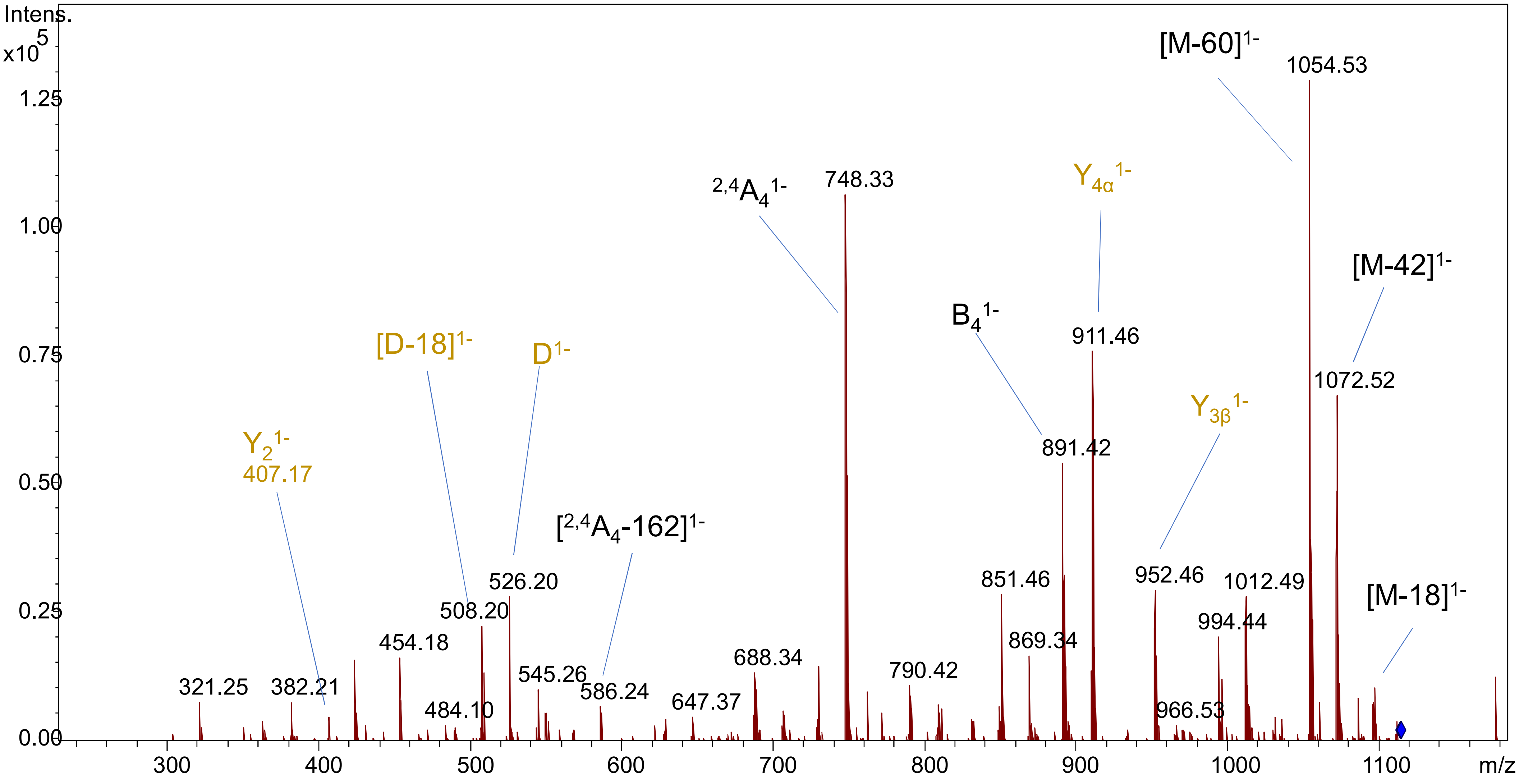

Glycan 37

A1G0F
(H3N3F1)

Monoisotopic mass: **1261.48 Da**
Charge observed: **1-**
Theoretical ion: ***m/z* 1260.47**
Observed ion: ***m/z* 1260.66**
Mass deviation: ***m/z* 0.09**
Retention time: **69.1 min**
Note: No MS2 was recorded.

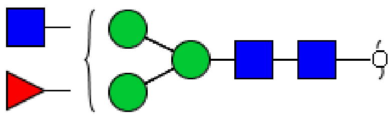

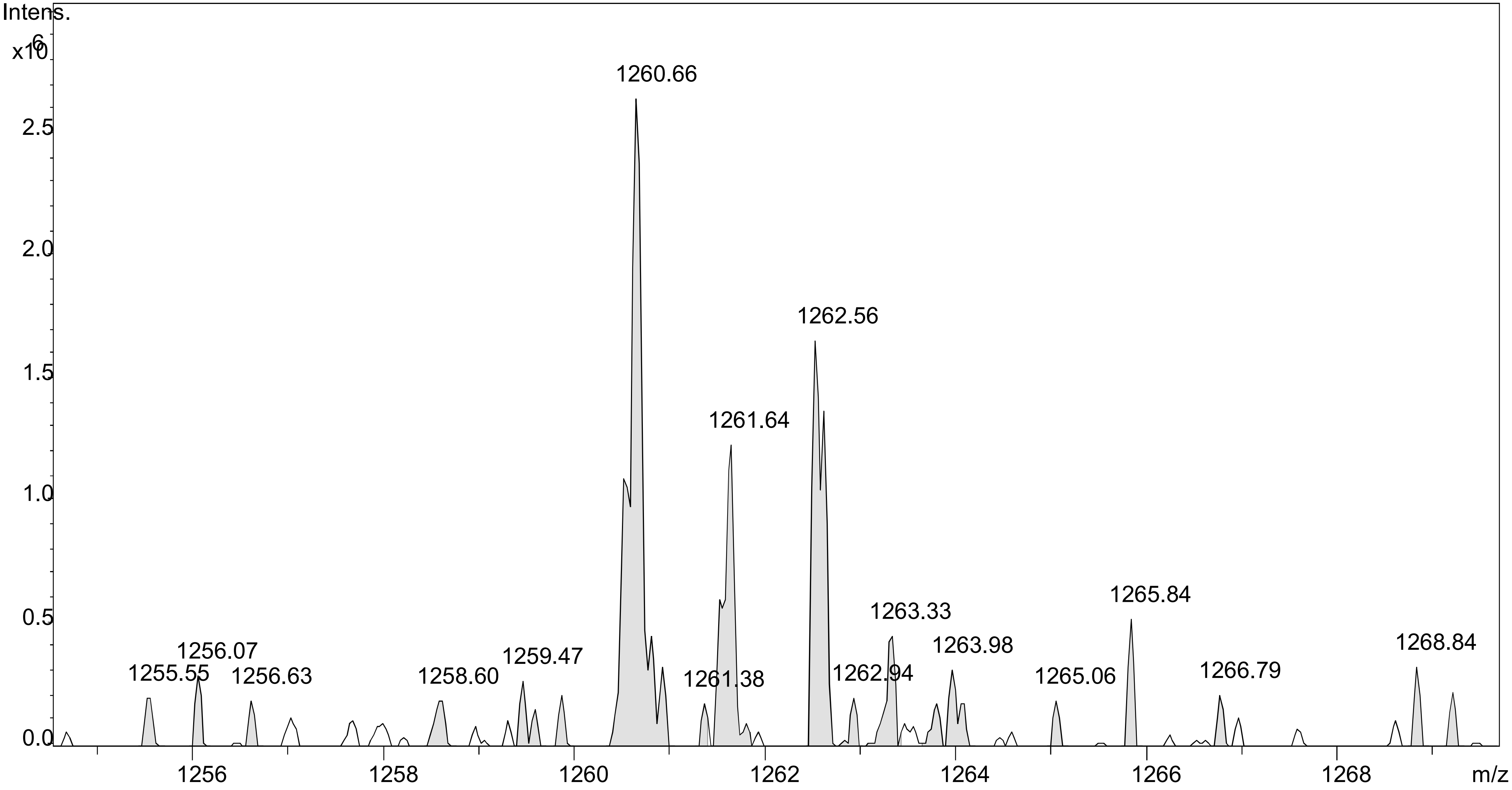

Glycan 38a

A1G0-M4
(H4N3)

**Monoisotopic mass:**	**1277.47 Da**
**Charge observed:**	**1-**
**Theoretical ion:**	***m/z* 1276.47**
**Observed ion:**	***m/z* 1276.63**
**Mass deviation:**	***m/z* 0.16**
**Retention time:**	**43.6 min**

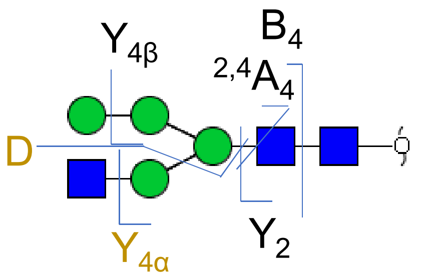

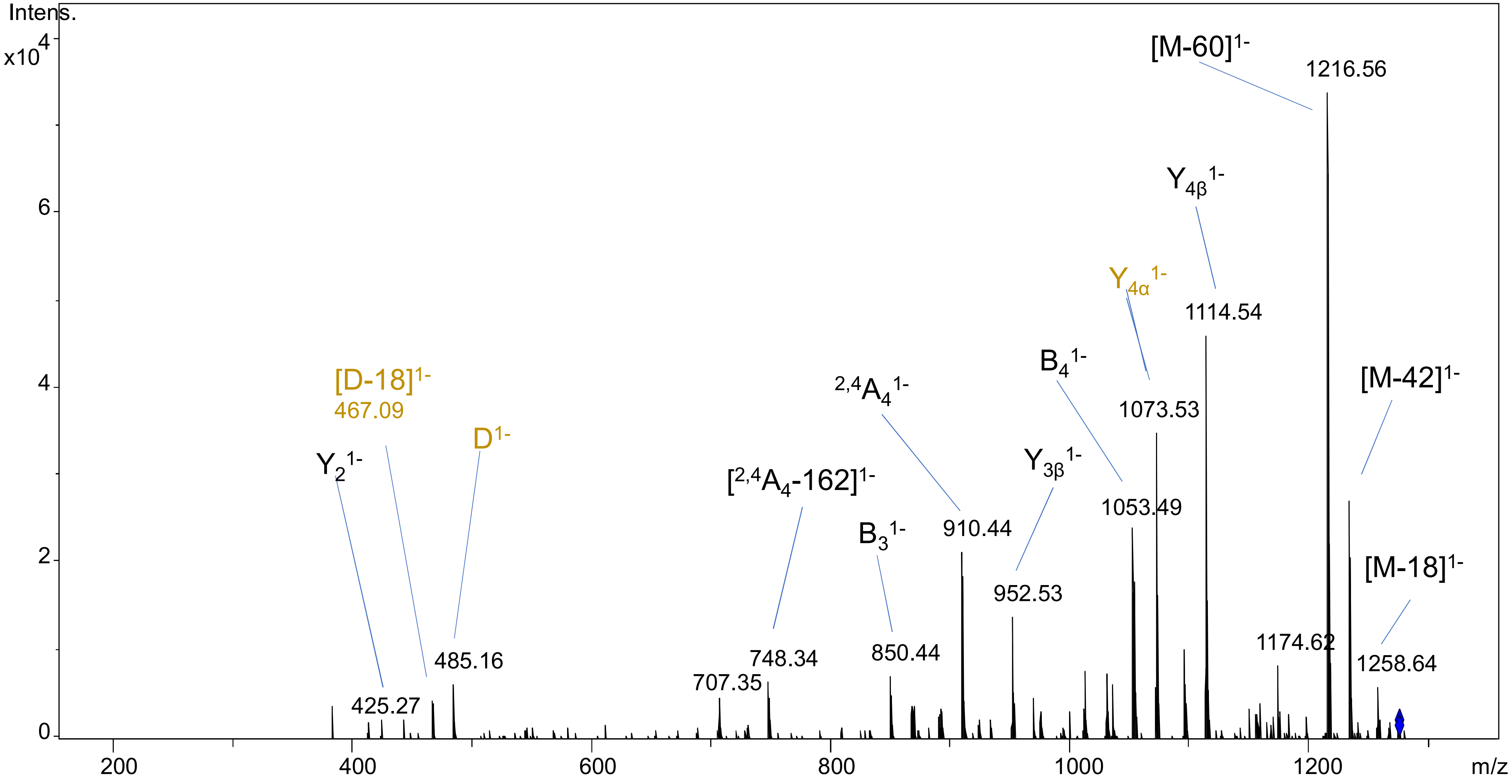

Glycan 38b

A1G1
(H4N3)

**Monoisotopic mass:**	**1277.47 Da**
**Charge observed:**	**1-**
**Theoretical ion:**	***m/z* 1276.47**
**Observed ion:**	***m/z* 1276.61**
**Mass deviation:**	***m/z* 0.14**
**Retention time:**	**50.4 min**

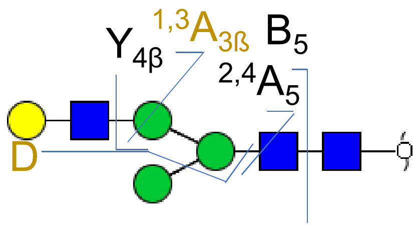

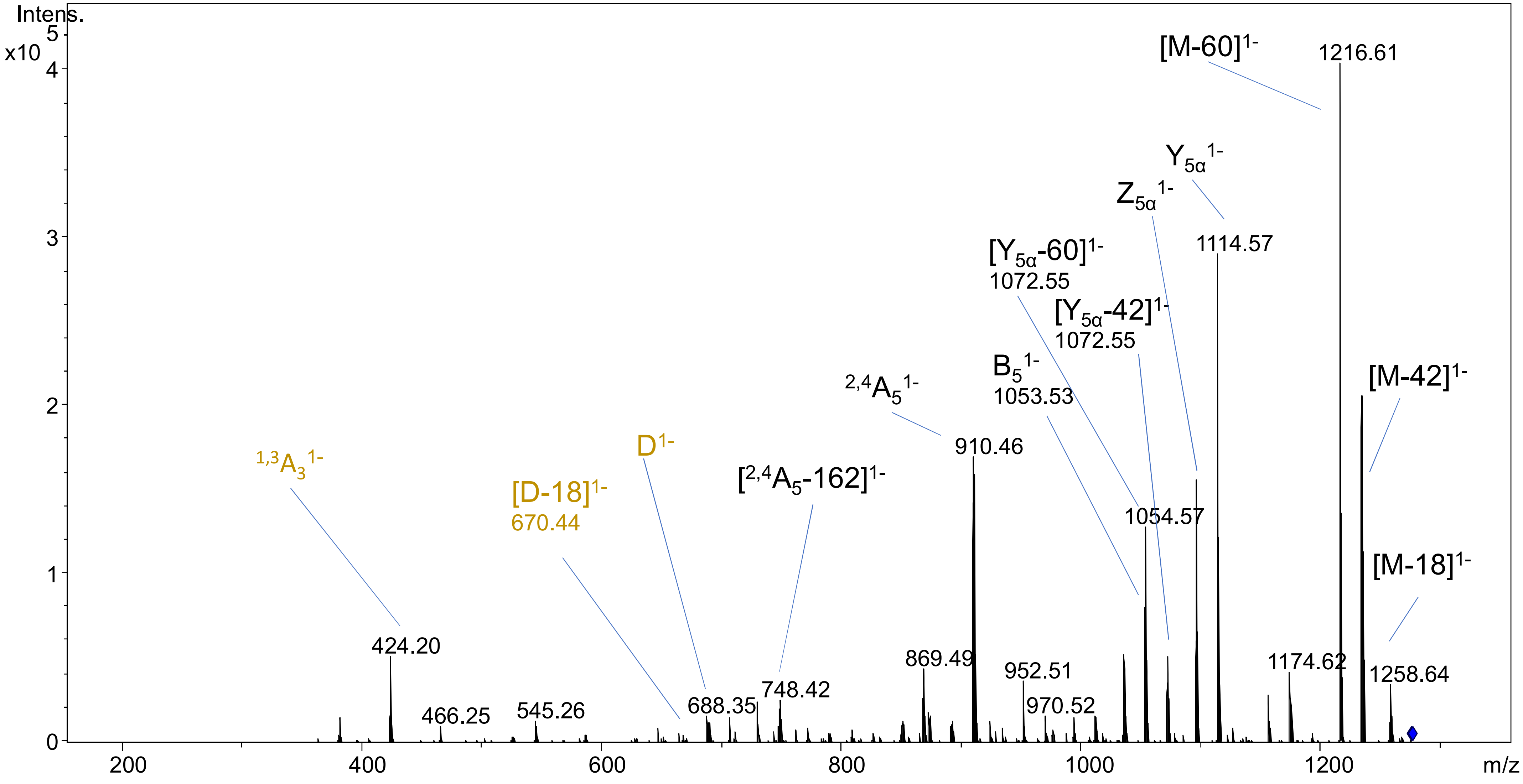

Glycan 39

A1S1
(H4N3S1)

**Monoisotopic mass:**	**1567.56 Da**
**Charge observed:**	**1-**
**Theoretical ion:**	***m/z* 783.28**
**Observed ion:**	***m/z* 783.39**
**Mass deviation:**	***m/z* 0.11**
**Retention time:**	**60.0 min**

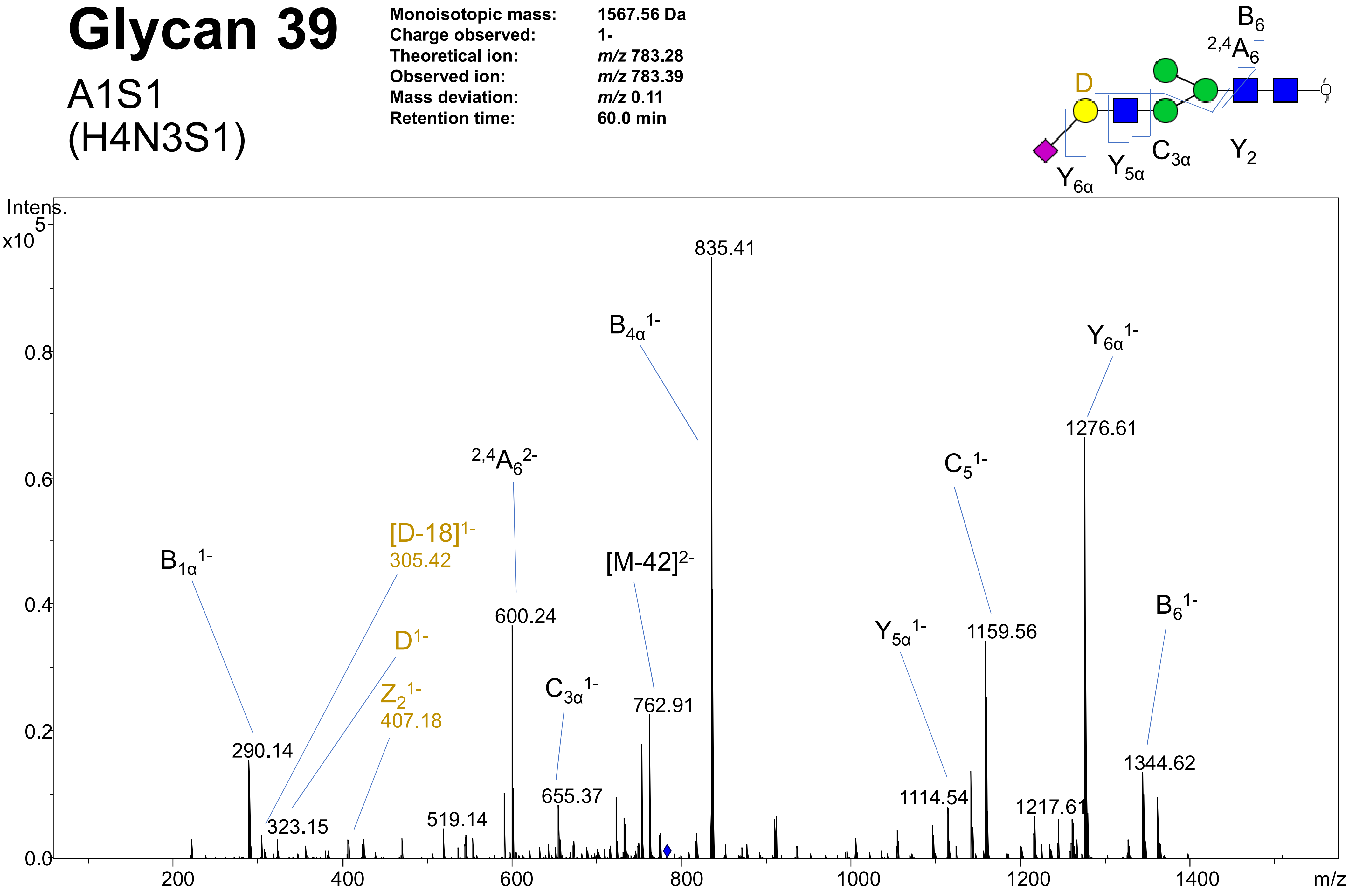

Glycan 40

A1G1-M5
(H6N3)

**Monoisotopic mass:**	**1601.59 Da**
**Charge observed:**	**2-**
**Theoretical ion:**	***m/z* 799.78**
**Observed ion:**	***m/z* 799.88**
**Mass deviation:**	***m/z* 0.10**
**Retention time:**	**52.8 min**

UniCarb-DB: #2482

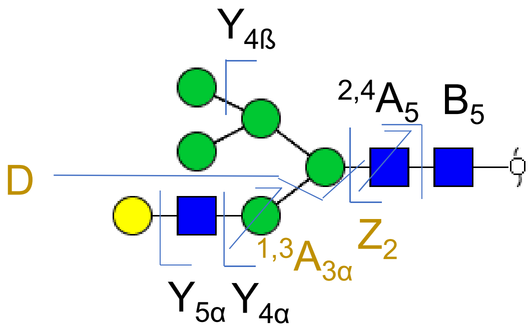

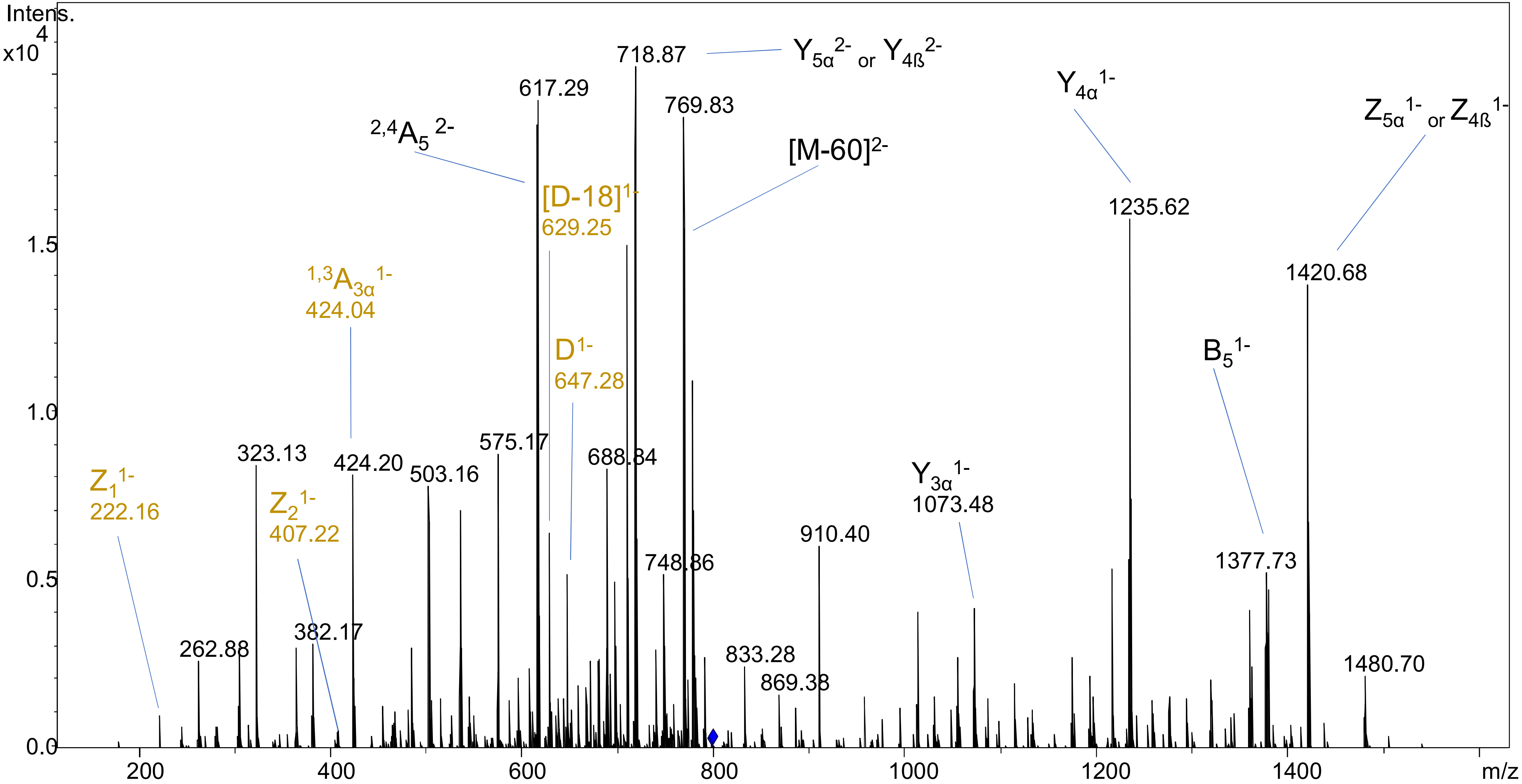

Glycan 41

A1G1-M5P
(H6N3P1)

Monoisotopic mass: **1681.55 Da**
Charge observed: **2-**
Theoretical ion: ***m/z* 839.77**
Observed ion: ***m/z* 839.84**
Mass deviation: ***m/z* 0.07**
Retention time: **39.0 min**
Note: Isomer to glycan 12.

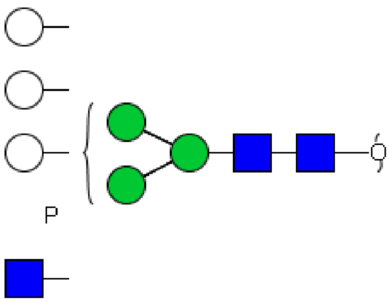

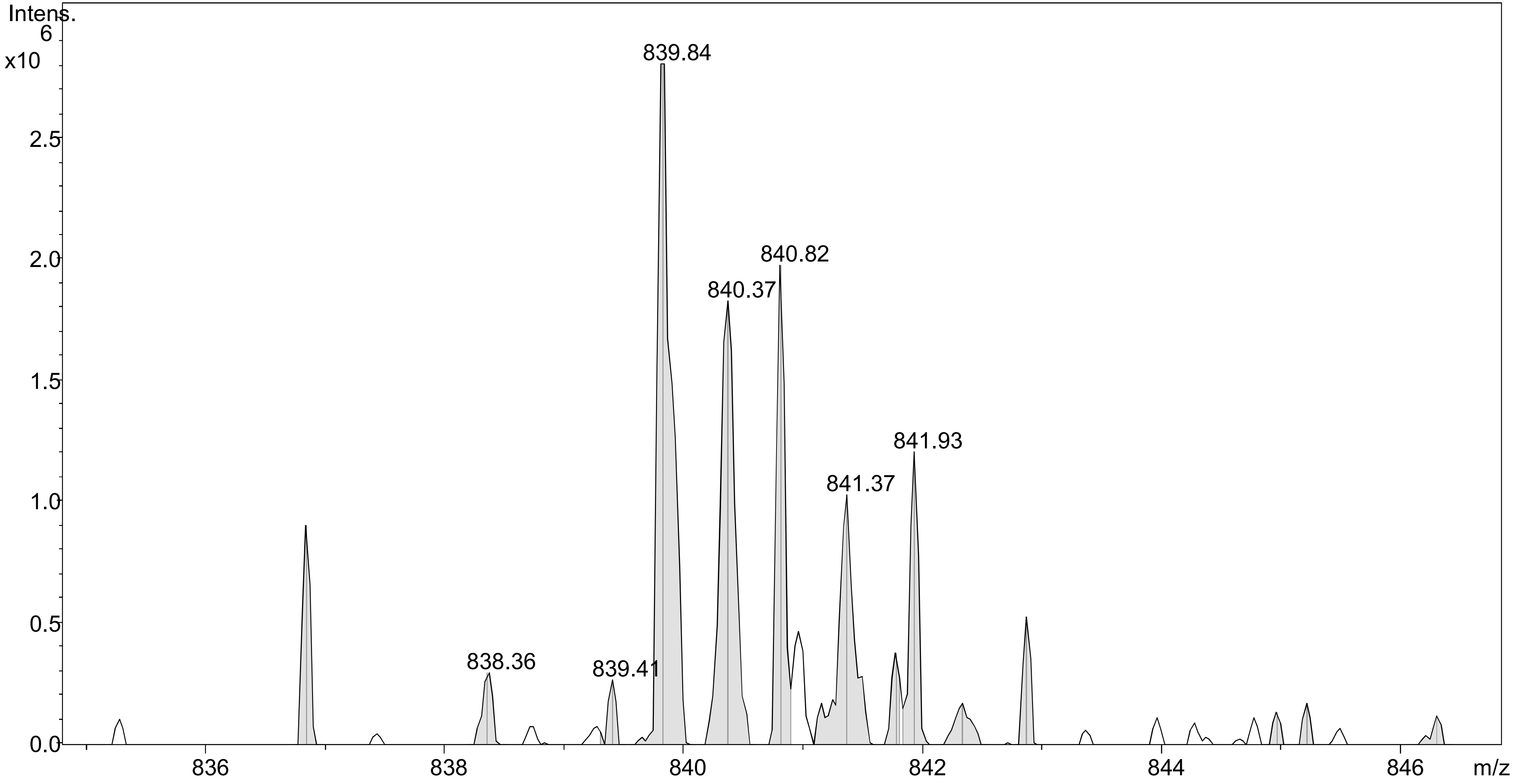

Glycan 42a

A1S1F
(H4N3F1S1)

**Monoisotopic mass:**	**1714.63 Da**
**Charge observed:**	**2-**
**Theoretical ion:**	***m/z* 856.30**
**Observed ion:**	***m/z* 856.43**
**Mass deviation:**	***m/z* 0.13**
**Retention time:**	**66.6 min**

UniCarb-DB: #2220

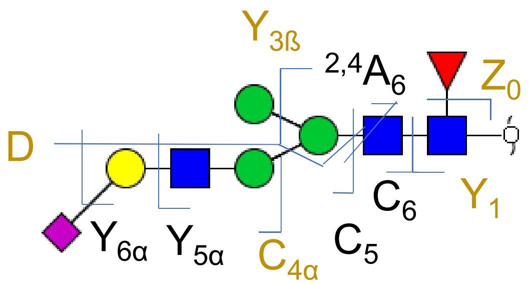

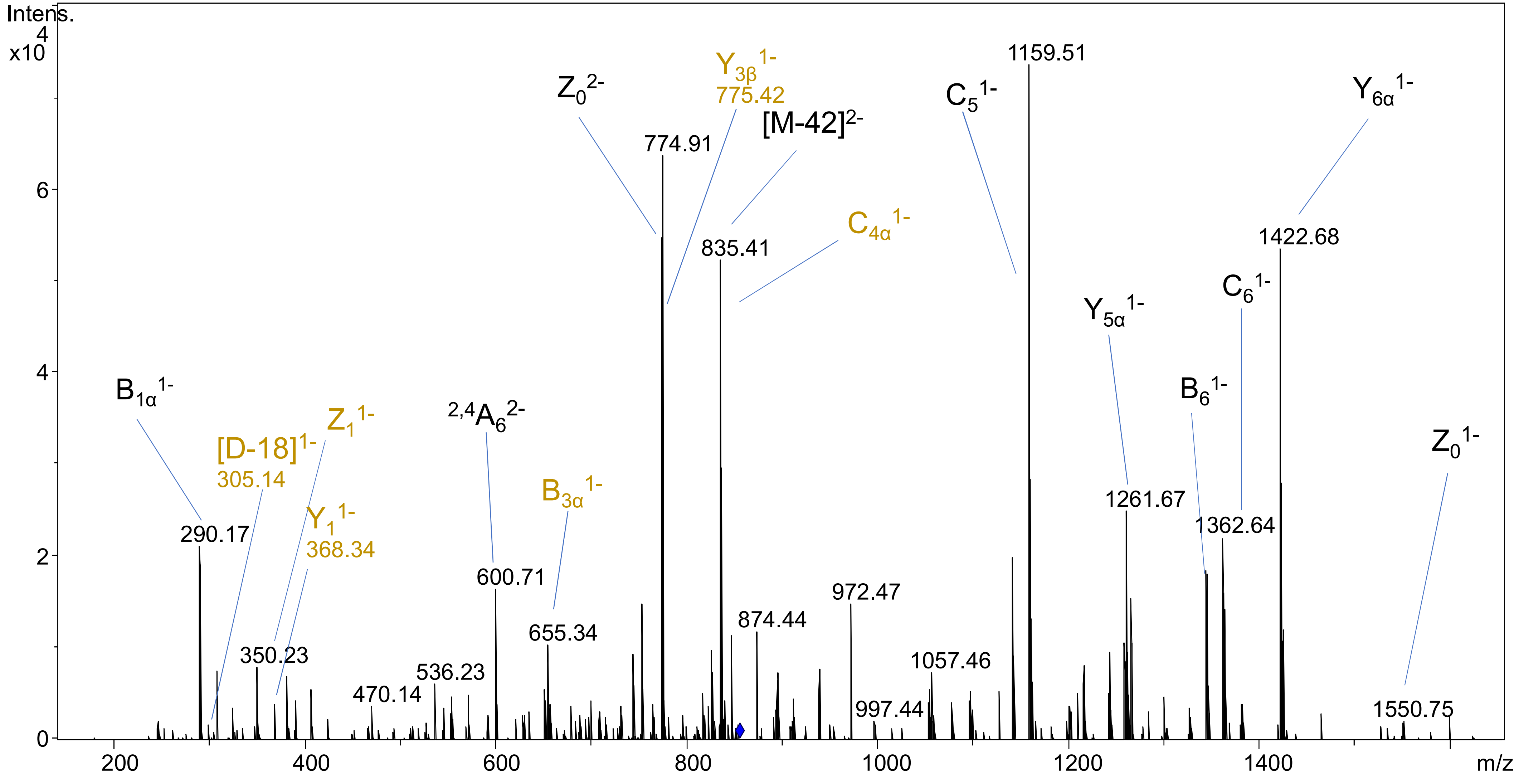

Glycan 42b

A1S1F

(H4N3F1S1)

**Monoisotopic mass:**	**1714.63 Da**
**Charge observed:**	**2-**
**Theoretical ion:**	***m/z* 856.30**
**Observed ion:**	***m/z* 856.46**
**Mass deviation:**	***m/z* 0.16**
**Retention time:**	**77.1 min**

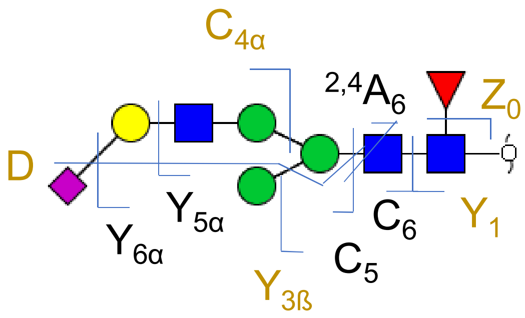

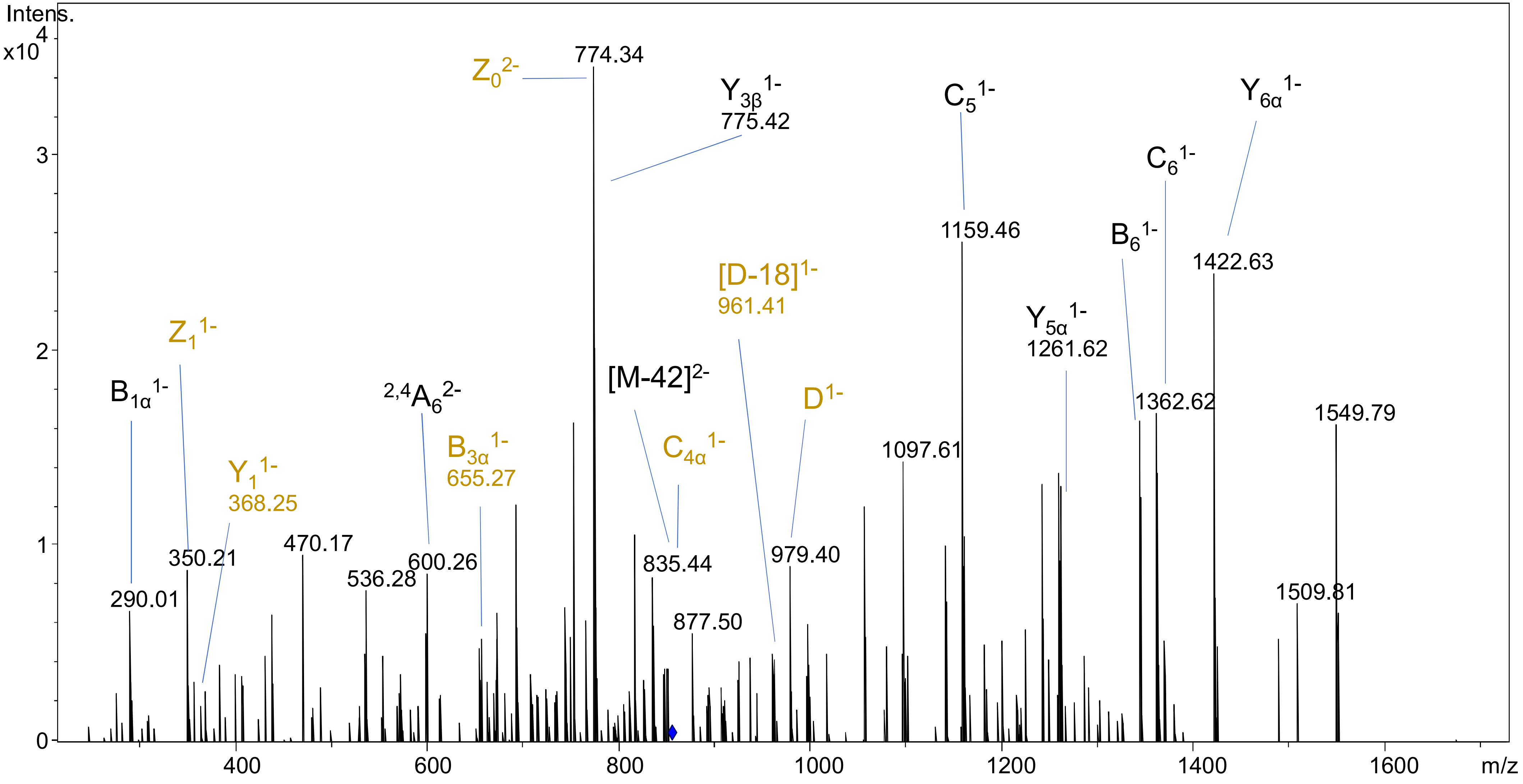

Glycan 43

A1S1-M4
(H5N3S1)

**Monoisotopic mass:**	**1730.62 Da**
**Charge observed:**	**2-**
**Theoretical ion:**	***m/z* 864.30**
**Observed ion:**	***m/z* 864.44**
**Mass deviation:**	***m/z* 0.14**
**Retention time:**	**58.6 min**

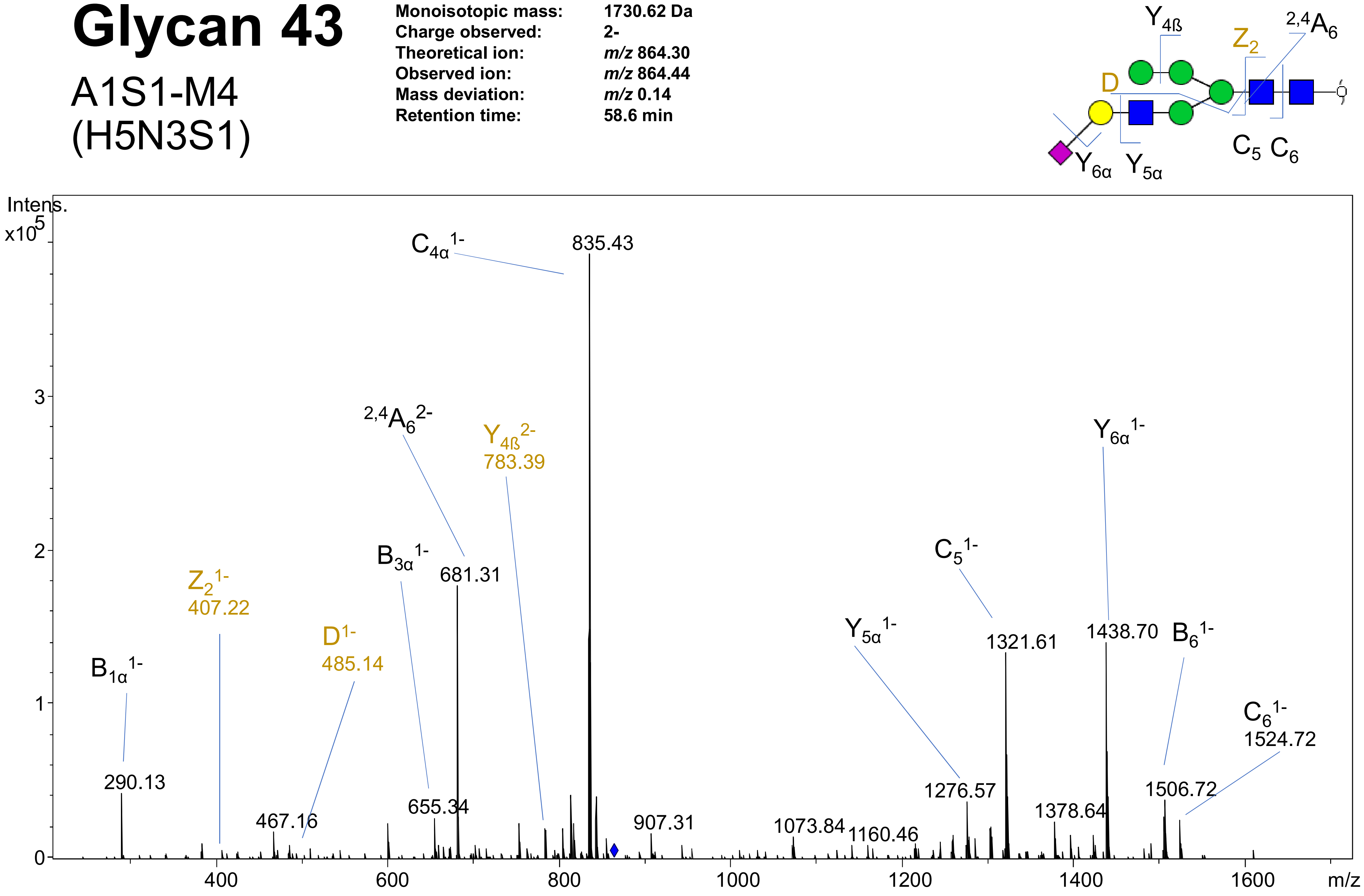

Glycan 44

A1G1F-M5P
(H6N3F1P1)

**Monoisotopic mass:**	**1827.60 Da**
**Charge observed:**	**2-**
**Theoretical ion:**	***m/z* 912.80**
**Observed ion:**	***m/z* 912.92**
**Mass deviation:**	***m/z* 0.12**
**Retention time:**	**47.0 min**

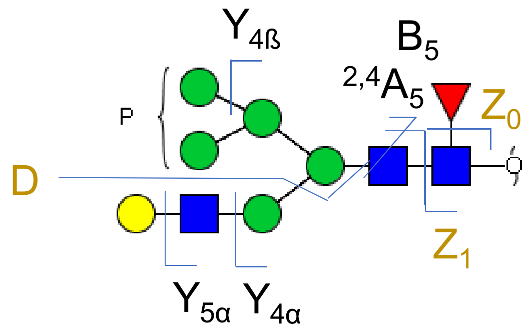

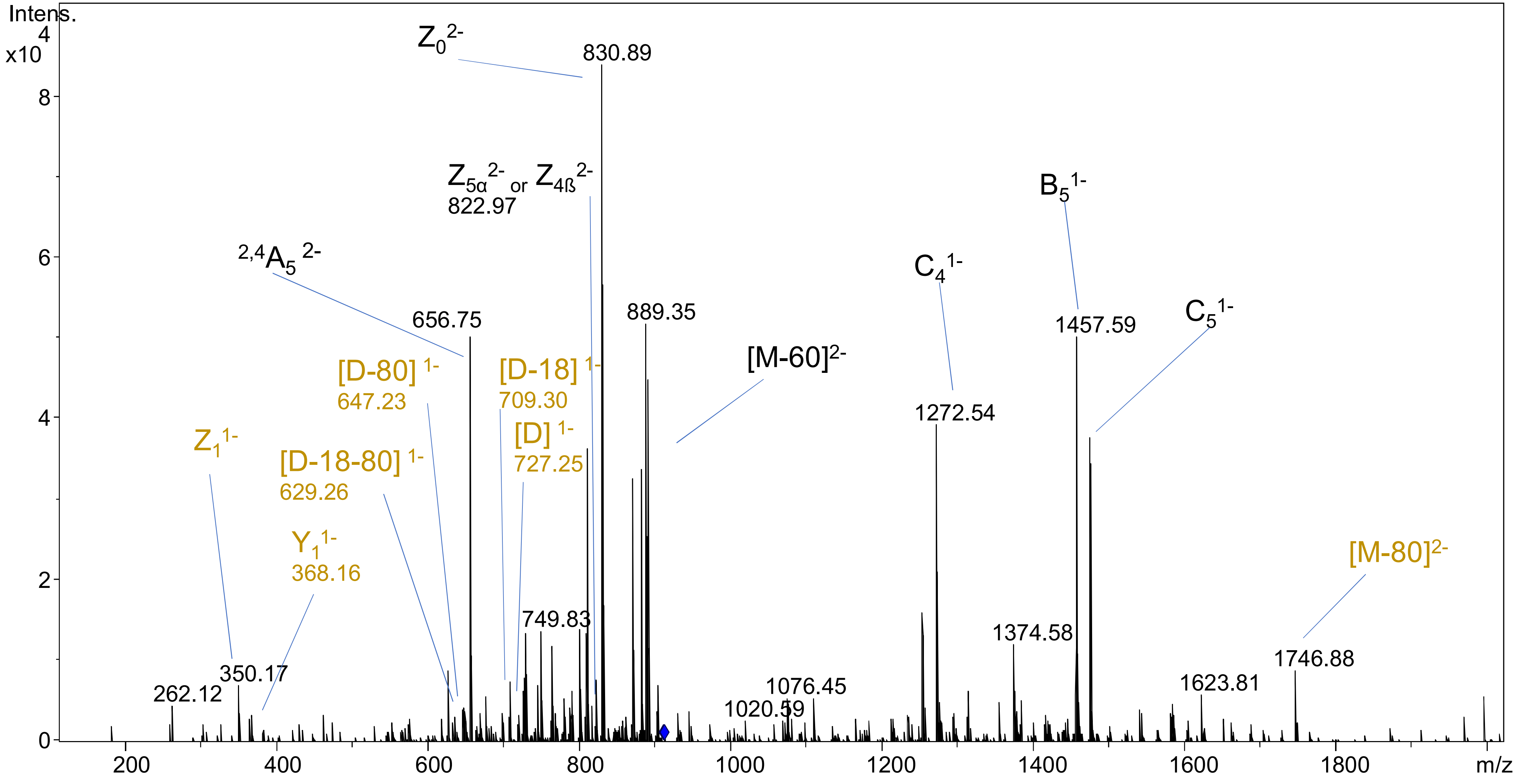

Glycan 45

A1G1-M6P
(H7N3P1)

Monoisotopic mass: 1843.60 Da
Charge observed: 2-
Theoretical ion: *m/z* 920.79
Observed ion: *m/z* 920.93
Mass deviation: *m/z* 0.14
Retention time: 41.4 min
Note: Isomer to glycan 15.

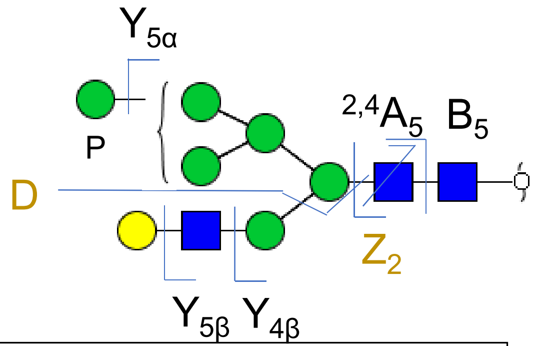

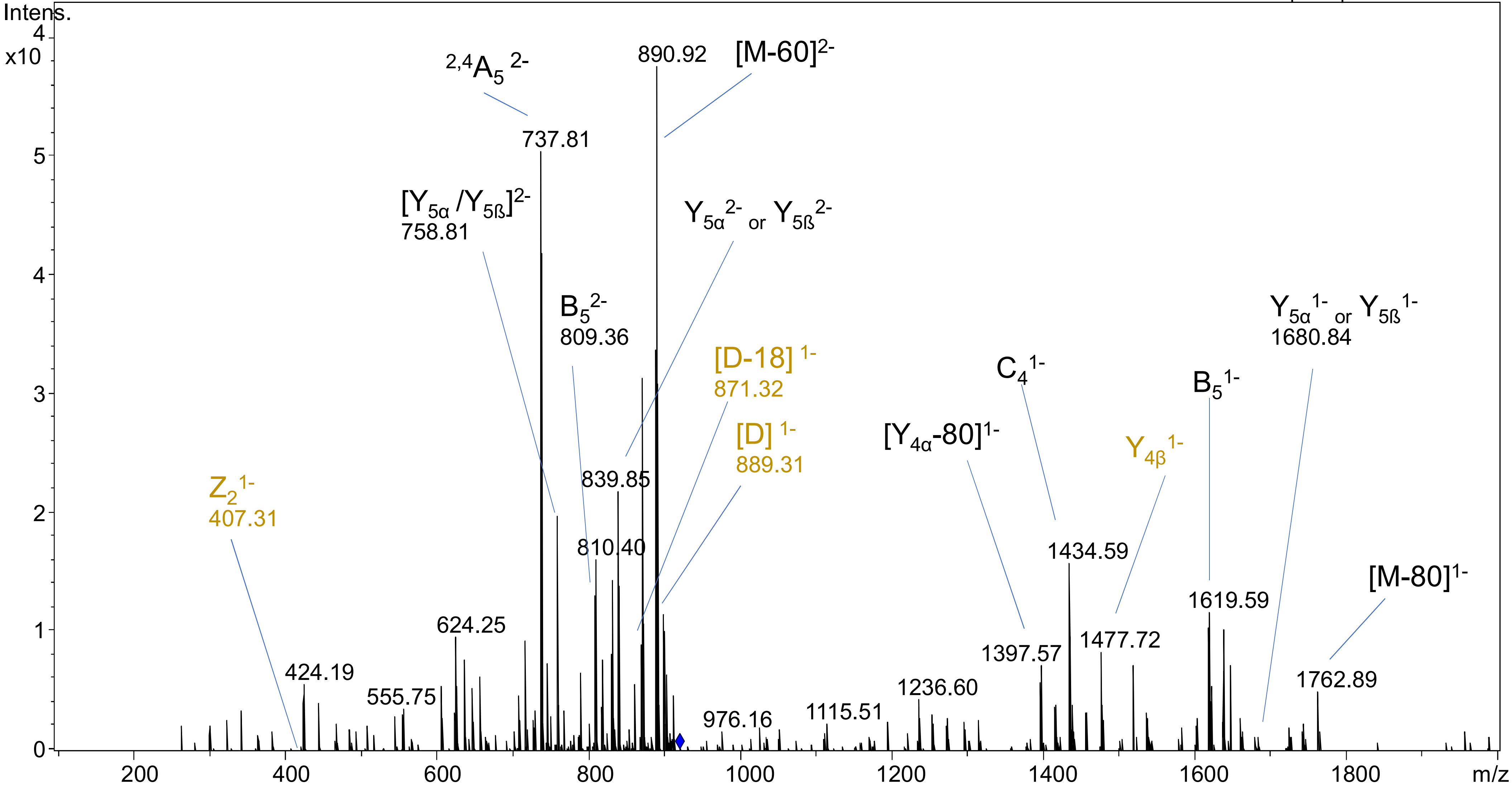

Glycan 46

A1S1-M5
(H6N3S1)

Monoisotopic mass: **1892.68 Da**
Charge observed: **2-**
Theoretical ion: ***m/z* 945.35**
Observed ion: ***m/z* 945.48**
Mass deviation: ***m/z* 0.01**
Retention time: **61.1 min**

UniCarb-DB: #2523

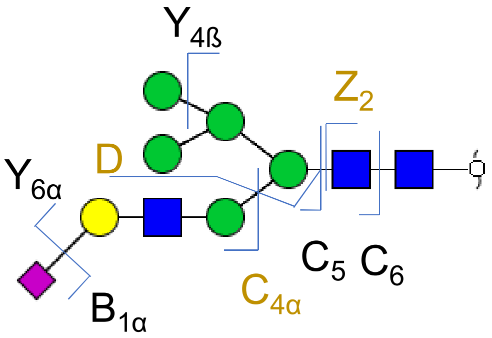

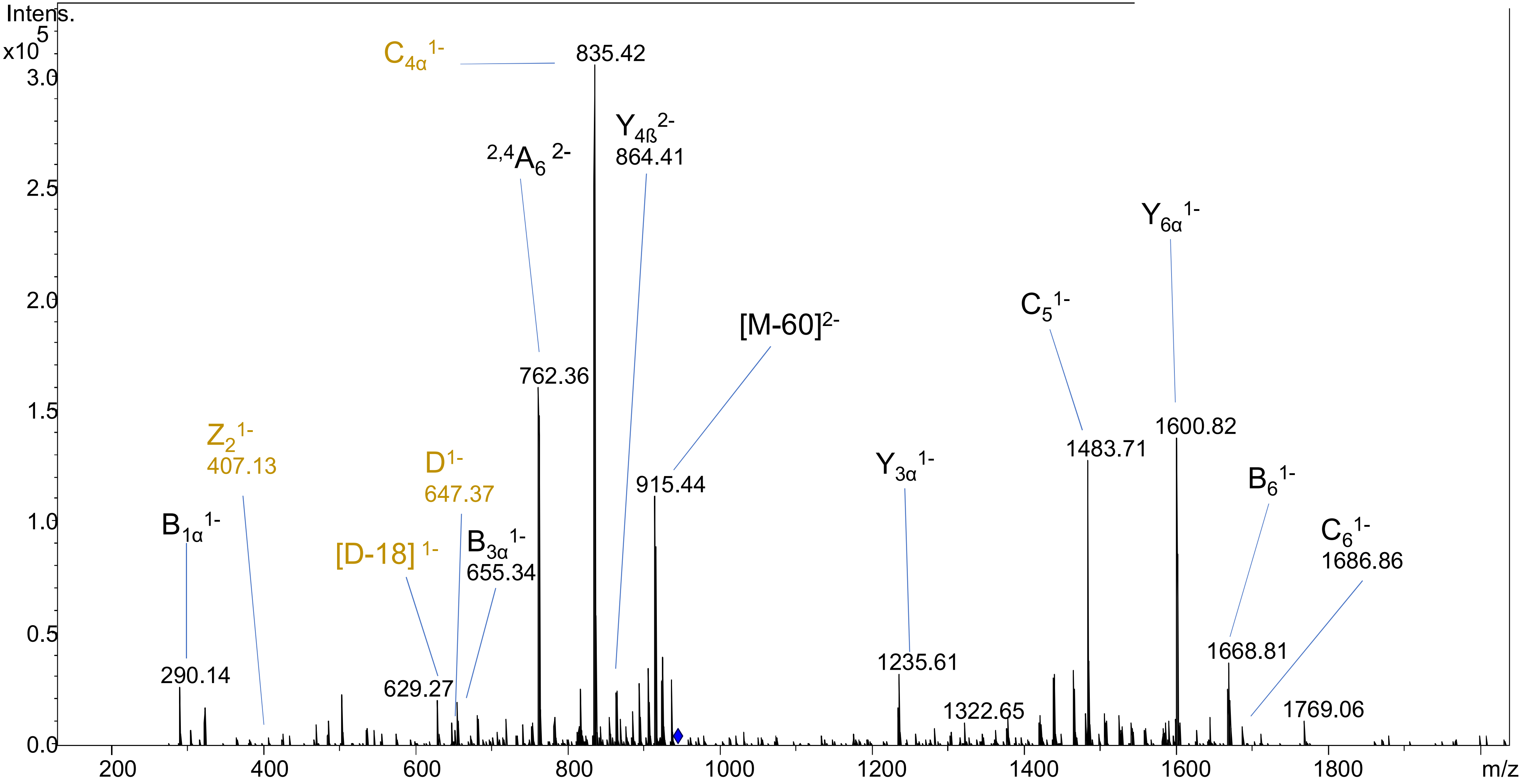

Glycan 47

A1G1F-M6P
(H7N3F1P1)

**Monoisotopic mass:**	**1989.65 Da**
**Charge observed:**	**2-**
**Theoretical ion:**	***m/z* 993.82**
**Observed ion:**	***m/z* 993.96**
**Mass deviation:**	***m/z* 0.14**
**Retention time:**	**48.7 min**

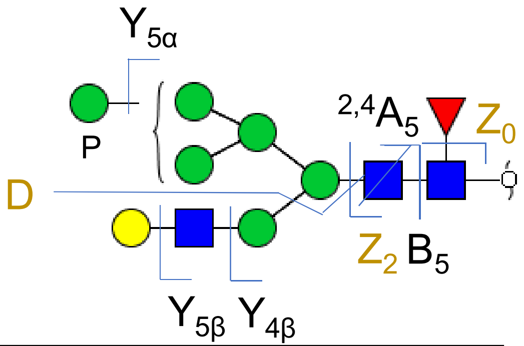

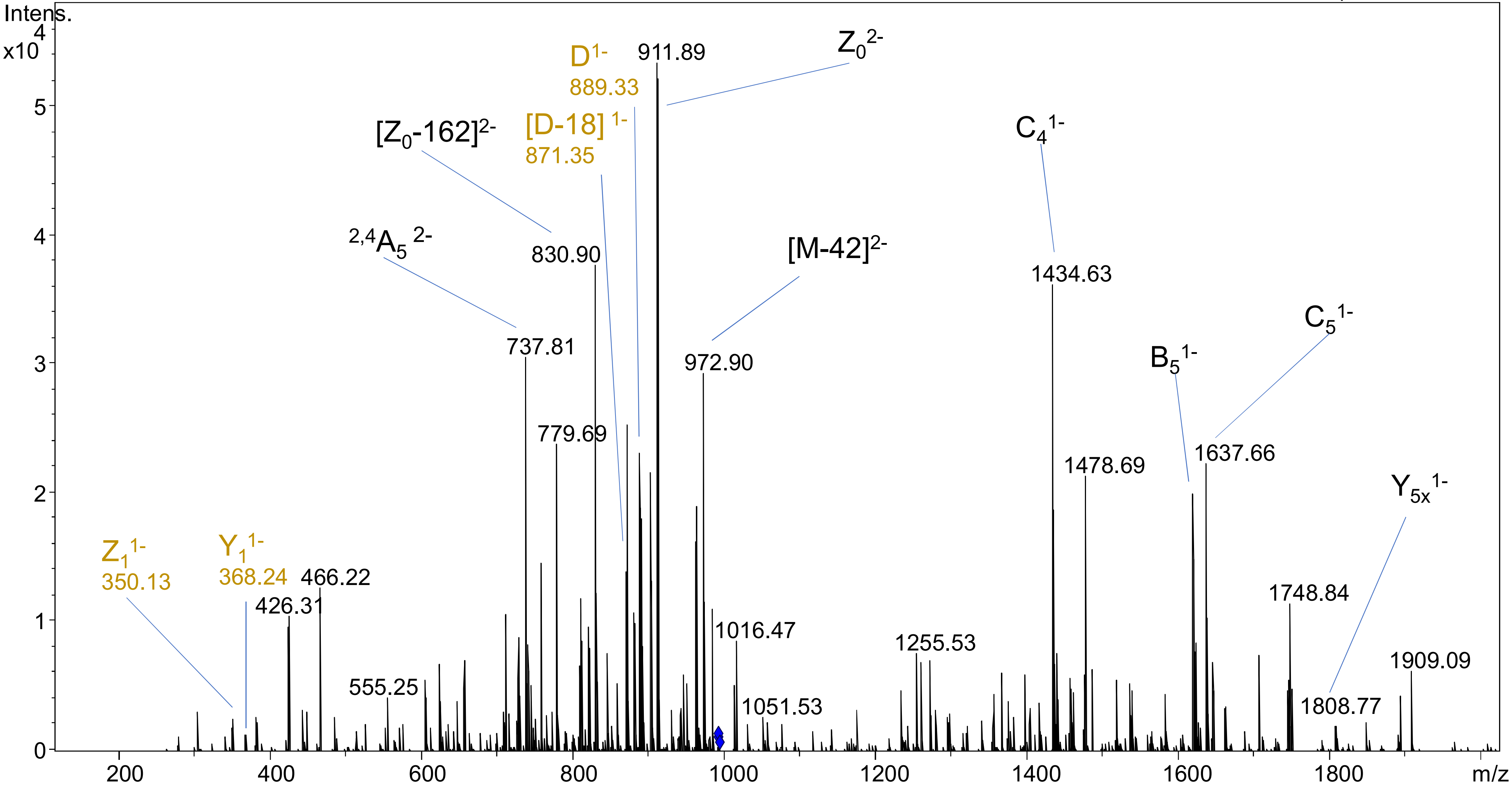

Glycan 48

A1S1-M6P
(H7N3S1P1)

**Monoisotopic mass:**	**2134.69 Da**
**Charge observed:**	**2-**
**Theoretical ion:**	***m/z* 1066.34**
**Observed ion:**	***m/z* 1066.48**
**Mass deviation:**	***m/z* 0.14**
**Retention time:**	**51.0 min**

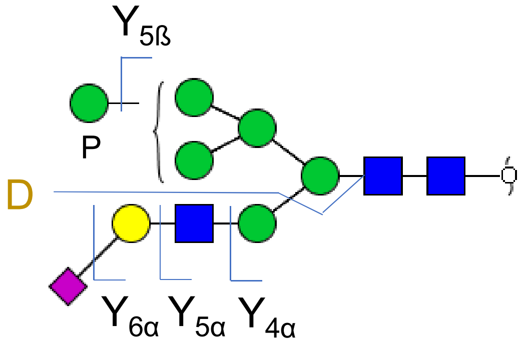

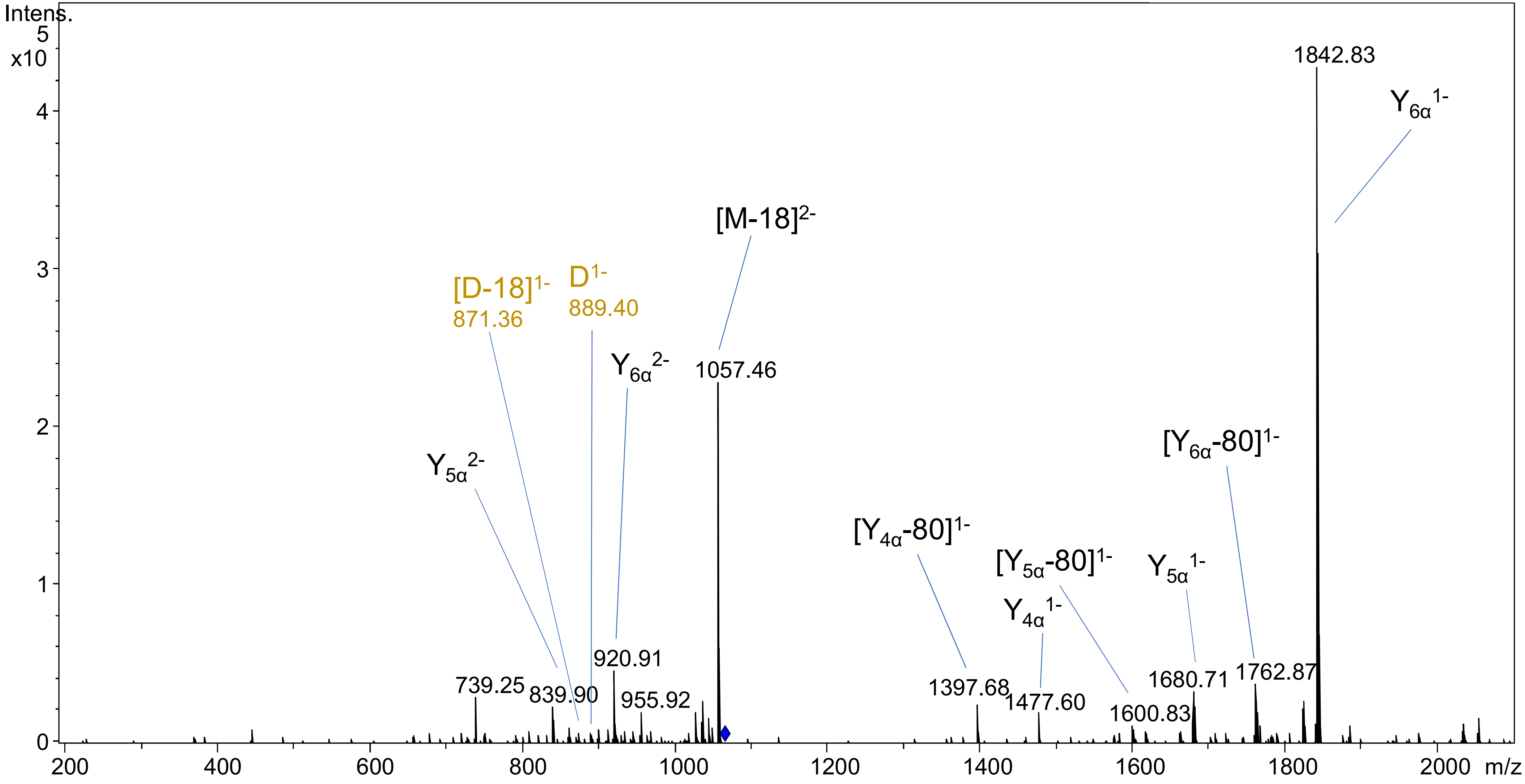

Glycan 49

A1S1F-M6P
(H7N3F1S1P1)

**Monoisotopic mass:**	**2280.75 Da**
**Charge observed:**	**2-**
**Theoretical ion:**	***m/z* 1139.37**
**Observed ion:**	***m/z* 1139.51**
**Mass deviation:**	***m/z* 0.13**
**Retention time:**	**56.5 min**

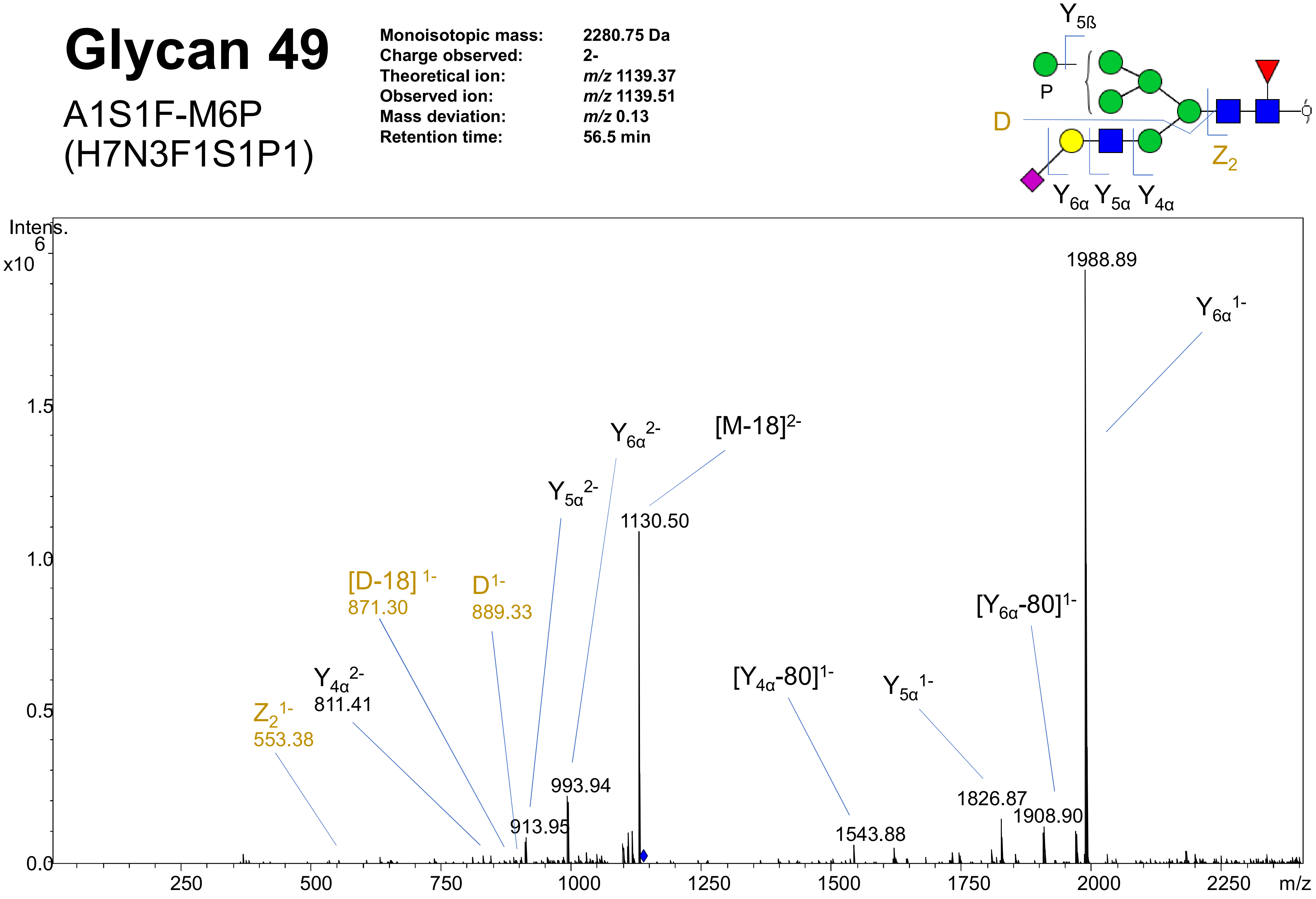

Glycan 50

A1S1-M6PGlcNAc
(H7N4S1P1)

**Monoisotopic mass:**	**2337.77 Da**
**Charge observed:**	**2-**
**Theoretical ion:**	***m/z* 1167.88**
**Observed ion:**	***m/z* 1168.00**
**Mass deviation:**	***m/z* 0.12**
**Retention time:**	**53.7 min**

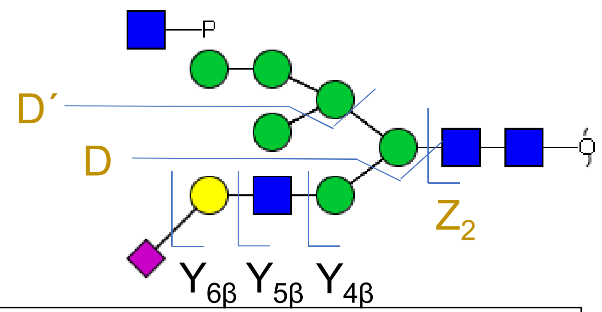

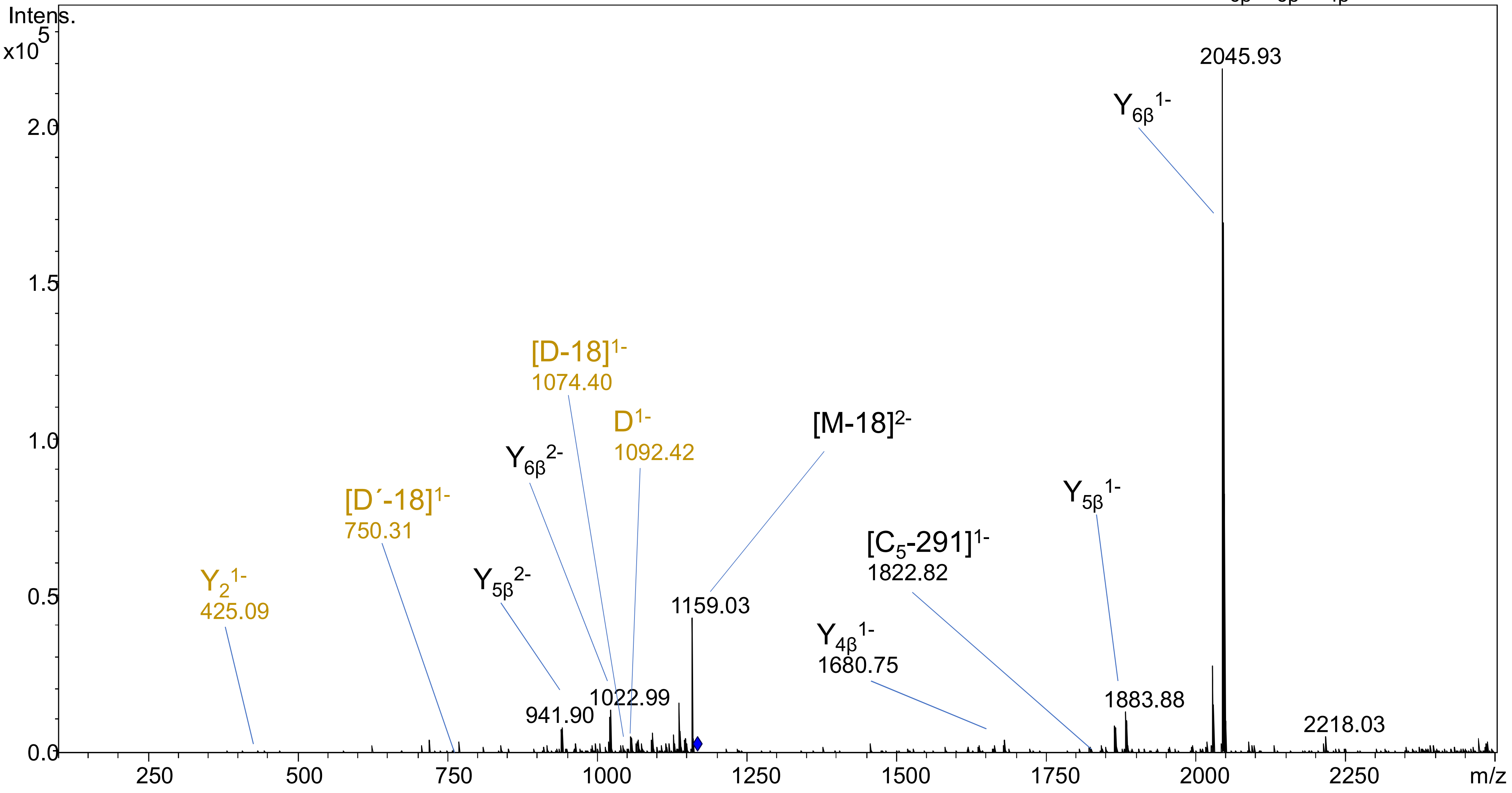

Glycan 51

A1S1F-M6PGlcNAc
(H7N4F1S1P1)

**Monoisotopic mass:**	**2483.83 Da**
**Charge observed:**	**2-**
**Theoretical ion:**	***m/z* 1240.90**
**Observed ion:**	***m/z* 1241.08**
**Mass deviation:**	***m/z* 0.18**
**Retention time:**	**58.7 min**

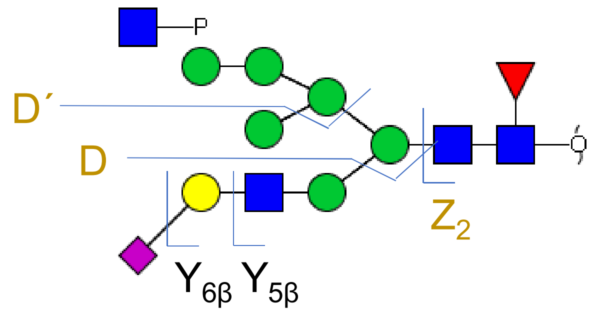

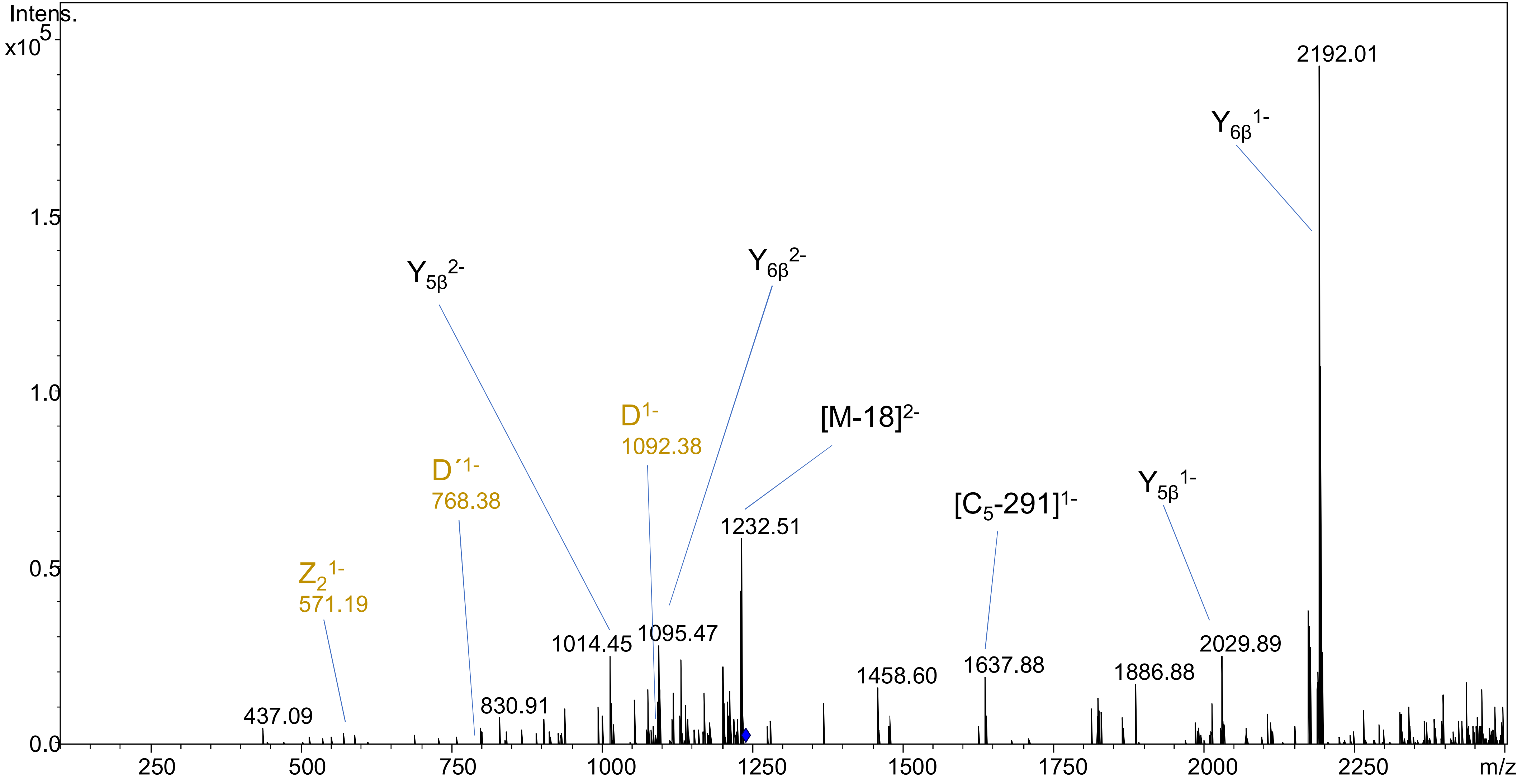

Supplement: 20221212_N-Glycan_Structure_elucidation [file mmc2.pdf]
